# MicroRNAs Are Involved in the Regulation of Ovary Development in the Pathogenic Blood Fluke *Schistosoma japonicum*

**DOI:** 10.1371/journal.ppat.1005423

**Published:** 2016-02-12

**Authors:** Lihui Zhu, Jiangping Zhao, Jianbin Wang, Chao Hu, Jinbiao Peng, Rong Luo, Chunjing Zhou, Juntao Liu, Jiaojiao Lin, Youxin Jin, Richard E. Davis, Guofeng Cheng

**Affiliations:** 1 Shanghai Veterinary Research Institute, Chinese Academy of Agricultural Sciences, Key Laboratory of Animal Parasitology, Ministry of Agriculture, Beijing, China; 2 Departments of Biochemistry and Molecular Genetics, University of Colorado School of Medicine, Aurora, Colorado, United States of America; 3 School of Life Sciences, Shanghai University, Shanghai, China; University of Texas Southwestern Medical Center at Dallas, UNITED STATES

## Abstract

Schistosomes, blood flukes, are an important global public health concern. Paired adult female schistosomes produce large numbers of eggs that are primarily responsible for the disease pathology and critical for dissemination. Consequently, understanding schistosome sexual maturation and egg production may open novel perspectives for intervening with these processes to prevent clinical symptoms and to interrupt the life-cycle of these blood-flukes. microRNAs (miRNAs) are key regulators of many biological processes including development, cell proliferation, metabolism, and signal transduction. Here, we report on the identification of *Schistosoma japonicum* miRNAs using small RNA deep sequencing in the key stages of male-female pairing, gametogenesis, and egg production. We identified 38 miRNAs, including 10 previously unknown miRNAs. Eighteen of the miRNAs were differentially expressed between male and female schistosomes and during different stages of sexual maturation. We identified 30 potential target genes for 16 of the *S*. *japonicum* miRNAs using antibody-based pull-down assays and bioinformatic analyses. We further validated some of these target genes using either *in vitro* luciferase assays or *in vivo* miRNA suppression experiments. Notably, suppression of the female enriched miRNAs bantam and miR-31 led to morphological alteration of ovaries in female schistosomes. These findings uncover key roles for specific miRNAs in schistosome sexual maturation and egg production.

## Author Summary

Schistosomes are parasitic worms that cause the neglected tropical disease schistosomiasis. Schistosomes infect > 200 million people and lead to considerable morbidity, which is primarily due to egg deposition and the ensuing host immune response. Pairing with a male is a prerequisite for female sexual development and subsequent egg production. Thus, understanding schistosome development and egg production is important to unravel biological processes contributing to the life cycle and to understand the basic processes leading to the pathogenicity of schistosomiasis. Here, we show that schistosome miRNAs play an important regulatory role in schistosome sexual maturation and ovary development. Suppression of female enriched miRNAs bantam and miR-31 results in morphological alternation of ovaries in female schistosomes. Our findings reveal key roles for miRNAs in schistosome reproductive biology.

## Introduction

Schistosomiasis is a human disease affecting over 200 million people worldwide and is caused by worms of the genus *Schistosoma* including *S*. *haematobium*, *S*. *mansoni*, and *S*. *japonicum* [1]. To date, no successful vaccine is available to prevent schistosomiasis [2]. The primary focus for control relies on chemotherapy using Praziquantel as the only widely applied drug [3,4]. This has raised serious concerns about the development of drug resistance, which would seriously compromise current treatment and control efforts [5]. This heavy reliance on a single drug and the risks it poses necessitates the identification of novel drug targets and/or the development of alternative strategies for schistosomiasis control.

Schistosomes are flatworms that are dioecious. Pairing of male and female worms is a prerequisite for female development and subsequent egg production [6–10]. The eggs are the major cause of pathogenesis of schistosomiasis and are essential for transmission of the disease [10–12]. Therefore, it is important to understand the molecular basis of schistosome sexual maturation and egg production.

Previous studies indicated that a continuous pairing contact is critical for female development [6–10,13]. Unmated female schistosomes are stunted in size and remain sexually immature. When paired female worms are separated from male worms, they cease egg laying and regress to an immature state. Re-introduction of males and their pairing with these immature females enables them to mature again [10,14–19]. Male-female pairing stimulates gamete development in females and leads to increased fertilization rates. Genomic [20], proteomic [10,12,21], and transcriptomic [21–25] studies have been used to interrogate the molecular basis of schistosome development and sexual maturation. Studies on male-female pairing [10,22,26–29] suggest that male schistosomes provide a key developmental signal that leads to female sexual maturation and egg production [13,15,30–37] *ex reproduction*.*the gs*. For example, transforming growth factor β (TGF-β) signaling is involved in the development of female vitelline cells and egg embryogenesis as a consequence of the direct interaction with male schistosome [38–42]. Furthermore, *S*. *mansoni* tyrosine kinases have been implicated in the regulation of schistosome gametogenesis [43]. Overall, these and other studies suggest that there are complex interactions within and between males and females that regulate female sexual maturation and egg production.

miRNAs, a class of small regulatory RNAs, are involved in the regulation of many biological processes primarily through the repression of messenger RNAs by typically binding to the 3’ untranslated region (3’UTR) of target mRNAs. miRNAs have been identified in several schistosome species including *S*. *japonicum* [44–47] and *S*. *mansoni* [48–51] and developmental stages including cercariae [45], lung-stage schistosomula [45], hepatic-stage schistosomula [44,45,52,53], adult males and females [50,54], and eggs [44]. Studies have identified miRNAs ranging in numbers from a few up to as many as two thousand [55,56]. However, only 79 mature *S*. *japonicum* miRNAs and 225 mature *S*. *mansoni* miRNAs are currently documented in miRBase (Version 21).

Several studies have described miRNAs that are differentially expressed between male and female schistosomes [45,50]. Marco et al found that 13 miRNAs are differentially expressed between males and females in *S*. *mansoni* [50]. miR-1b, miR-61 and miR-281 were highly expressed in males whereas miR-8447, miR-2f, mir-8437, miR-31, bantam, miR-2c, miR-2d, miR-71b, miR-36b and miR-755 were highly expressed in females. In *S*. *japonicum*, Cai et al demonstrated miR-7-5p, miR-61, miR-219-5p, miR-125a, miR-125b, miR-124-3p, and miR-1 were dominant in males, while bantam, miR-71b-5p, miR-3479-5p and miR-Novel-23-5p were predominantly found in the female parasites [45]. Considering the critical role that eggs play in the pathogenesis of schistosomiasis, miRNAs in schistosome eggs have also been analyzed and several miRNAs (sja-miR-71b-5p, sja-miR-71, sja-miR-1, sja-miR-36-3p, and sja-124-3p) were shown to be the most abundant in the egg stage [44]. In addition, the key molecules involved in miRNA biogenesis including Dicer [57–59], Argonaute proteins [57–60], and Drosha [58,59] have also been identified and shown to be differentially expressed in different stages and sexes of schistosomes. These studies suggest that miRNAs may act as important regulators in schistosome development, sexual maturation, and egg production. However, their functional and regulatory roles remain poorly characterized in schistosomes.

In this study, we used high-throughput small RNA sequencing to systemically identify *S*. *japonicum* miRNAs in the stages associated with male-female interaction, gametogenesis, and egg production. Using an Argonaute antibody-based pull-down assay and bioinformatic analyses, we identified the putative target genes for several of these miRNAs. Several of these targets were further validated by either *in vitro* luciferase assays or *in vivo* miRNA suppression experiments. In addition, suppression of female enriched miRNAs such as miR-31 and bantam led to morphological changes in the ovaries of female schistosomes. Overall, the results of our study indicate that miRNAs play a vital role in sexual maturation in *S*. *japonicum*. The development of a new strategy to target these miRNAs and thus reduce the egg production may help decrease parasite pathology and transmission in schistosomes.

## Results

### *S*. *japonicum* miRNAs

**Fig 1:**
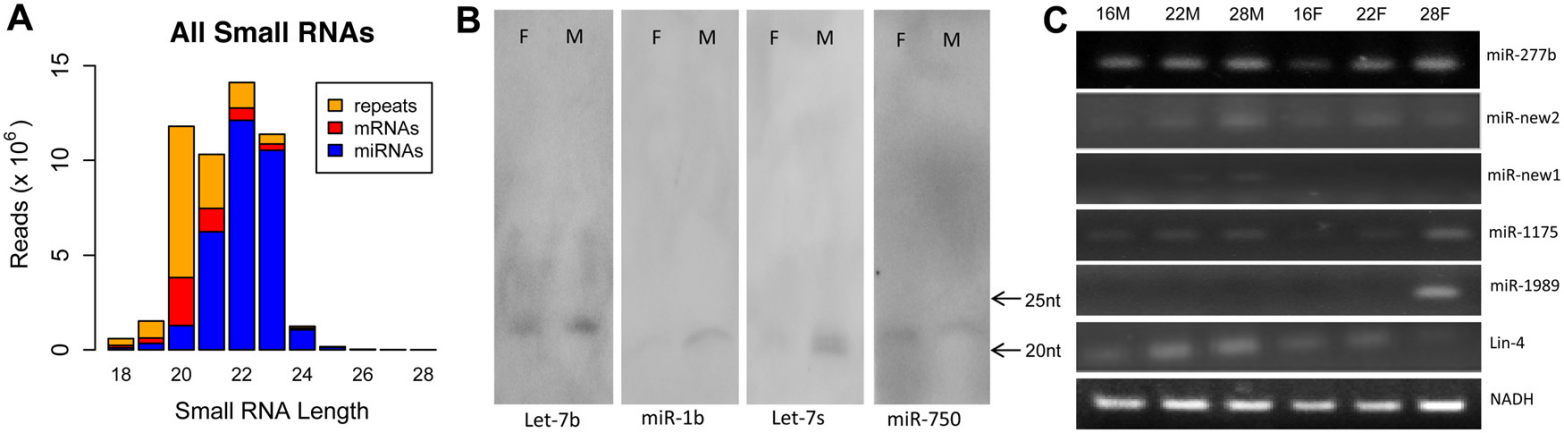
Classification of *S*. *japonicum* small RNAs and analysis of newly identified *S*. *japonicum* miRNAs. (A) Classification of *S*. *japonicum* small RNAs. Data represent the sum of small RNA reads from different stages and sexes for 8 *S*. *japonicum* libraries. Small RNAs that correspond to repetitive sequences (repeats, orange) and mRNA transcripts (red) are likely endo-siRNAs. (B) Northern blot analysis for *S*. *japonicum* miRNAs including let-7b, miR-1b, let-7s and miR-750. Total RNAs were isolated from 26-day old male and female *S*. *japonicum*. (C) Semi-quantitative RT-PCR analysis for the expression of the novel miRNAs in different stages and sexes of *S*. *japonicum*. 16M, 16-day old male; 22M, 22-day old male; 28M, 28-day old male; 16F, 16-day old female; 22F, 22-day old female; 28F, 28-day old female.

To identify *S*. *japonicum* miRNAs associated with male-female interaction, sexual development, and/or egg production, we isolated schistosomes from rabbits infected with *S*. *japonicum* cercariae at 16, 22, and 28 days post-infection, respectively. These stages represent the key stages of pairing initiation, gametogenesis, and egg production (S1 Fig). Paired males and females were separated, their RNA isolated, and small RNAs analyzed by deep sequencing. Eight small RNA libraries were prepared and analyzed including 16-day old females (16F), 16-day old males (16M), 22-day old females (22F), 22-day old males (22M), 28-day old females (28F), 28-day old males (28M), and mixed males (M) and mixed females (F). Bioinformatic analyses indicated that the majority of the small RNAs in these libraries were miRNAs and repeat associated siRNAs (S2 Fig). The majority of *S*. *japonicum* miRNAs are 21–23 nt in length, while repeat associated siRNAs are 20 nt in length (Fig 1A and S2B and S2C Fig).

The library sequences were subject to *de novo* analyses for miRNA identification (S1 Table) [61]. Using relatively stringent criteria for defining miRNAs (see S1 File for criteria used to identify miRNAs), we identified 38 miRNAs from these stages (S2 Table). Of these, 10 miRNAs were new to *S*. *japonicum* (lin-4, miR-1b, let-7b, let-7s, miR-277b, miR-750, miR-1175, miR-1989, miR-new1 and miR-new2) and 6 are new to schistosomes (let-7b, let-7s, miR-750, miR-1989, miR-new2, miR-277b) (Table 1). We used Northern blots to independently demonstrate the expression of four (let-7b, miR-1b, let-7s, and miR-750) of these newly identified *S*. *japonicum* miRNAs (Fig 1B and S3 Table). The other six miRNAs (lin-4, miR-1989, miR-277b, miR-1175, miR-new1 and miR-new2) were below the level of detection of our Northern blots. However, the expression of these *S*. *japonicum* miRNAs was verified using an RT-PCR method (Fig 1C and S4 Table).

**Table 1:** Novel miRNAs identified in *S*. *japonicum*.

Name	Sequences	Reads*	Hairpin locus
sja-let-7b	AGAGGUAGUGAUUCAUAUGACU	655,165	SJC_S000353: 80195–80367
sja-let-7s	GAGGUAGUUAGAUGUACGACU	111,993	SJC_S000383:436660–436832
sja-miR-750	CCAGAUCUGUCGCUUCCAACU	281,357	SJC_S000460:180604–180776
sja-miR-1175#	UGAGAUUCAAUUACUUCAACUG	20,657	SJC_S000460:180879–181001
sja-miR-1989	UCAGCUGUGUUCAUGUCUUCGA	2,812	SJC_S000128:160744–160623
sja-miR-new1#	GAGAGAGCACUUUUAUGACGGA	1,192	SJC_S004484:20079–20188
sja-miR-new2	AGCUAAAUAGGUUAGUUUGACUGUC	15,436	SJC_S016196:787–876
sja-lin-4#	UCCCUGAGACCUUAGAGUUGU	23,558	N/A
sja-miR-1b#	UGGAAUGUUGUGAAGUAUGUGC	1,412,190	N/A
sja-mir-277b	AAAAUGCAUCAUCUACCCUAGA	207,027	N/A

* The number is the sum of miRNA reads in all of the 8 libraries.

^#^ indicates that the miRNA has also been identified in *S*. *mansoni*.

### Validation of miRNA expression in *S*. *japonicum*

**Fig 2:**
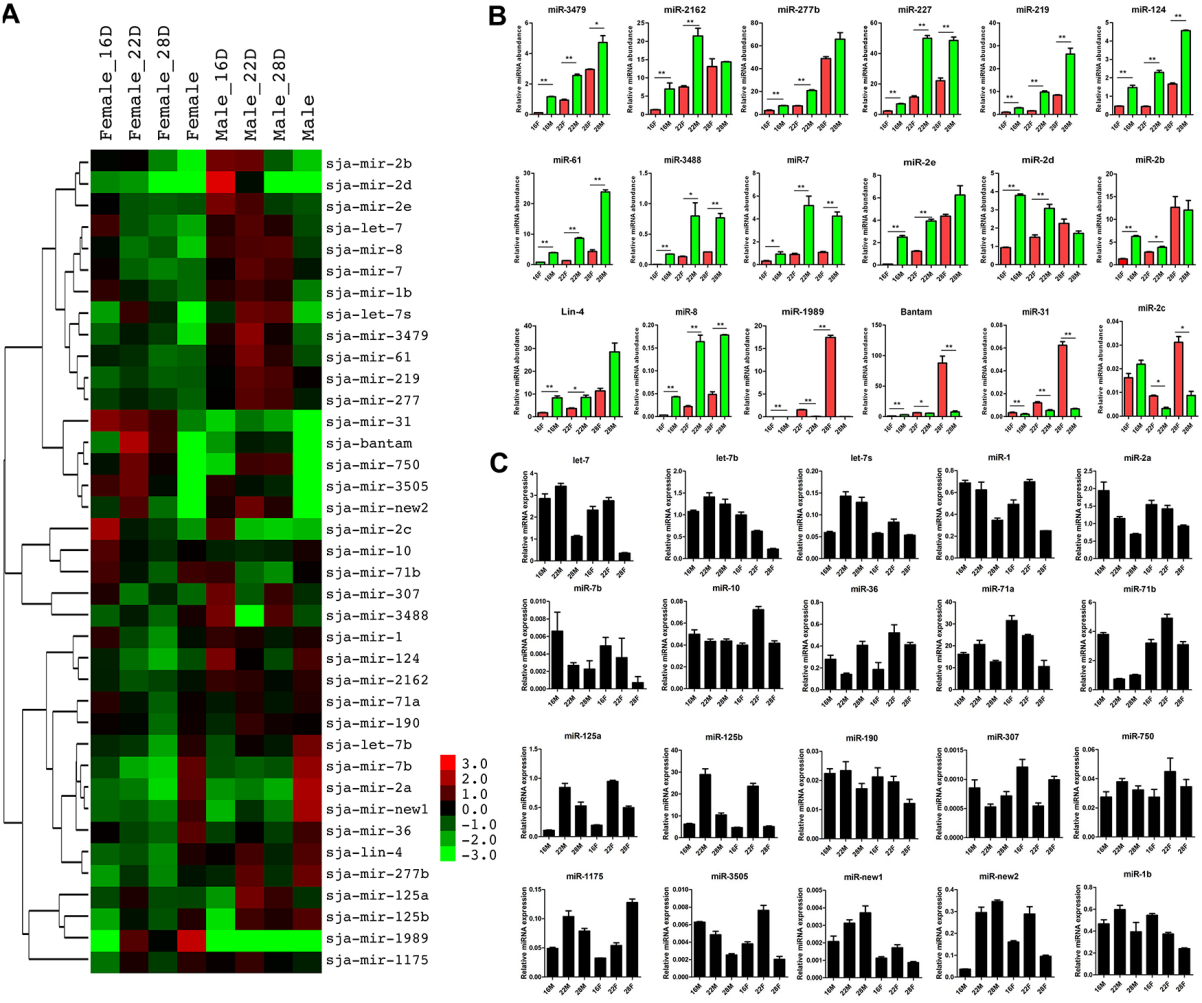
miRNA expression in *S*. *japonicum*. (A) Hierarchical clustering of the miRNAs in mature females (F) and males (M) and during sexual maturation. The heatmap was constructed based on the log2 fold change values of normalized miRNAs across these stages, with 0 (black) being the average miRNA level (see S2 Table for the normalized expression values). (B) Sex-enriched miRNA expression was determined using qRT-PCR and total RNA isolated from 16F, 16M, 22F, 22M, 28F and 28M. For graphical representation, the ΔCt method was used to evaluate the relative expression of transcripts of miRNAs between males and females [62].* means P ≤ 0.05 and ** means P ≤ 0.01 (student’s t test, females vs males). Data illustrate representative findings and show the mean and standard errors derived from triplicate experiments. (C) Analyses of miRNA expression in different stages of *S*. *japonicum*. The expressions of 20 miRNAs that were differently expressed but do not show sex-biased expressions in *S*. *japonicum* were validated using stem-loop based qRT-PCR. Total RNAs were isolated from 16F, 16M, 22F, 22M, 28F and 28M. Data illustrate representative results with the mean and standard error derived from quadruplicate experiments.

Analysis of miRNA expression using normalized reads (reads per million genome-matched reads [RPM]) demonstrated their differential expression in different stages of development and gender in *S*. *japonicum* (Fig 2A and S2 Table). Of the 38 *S*. *japonicum* miRNAs, 14 miRNAs were enriched in male worms whereas 4 miRNAs were predominantly found in females (Fig 2A and S2 Table). We further verified the differential expression of these sex-enriched miRNAs by qRT-PCR using independently prepared total RNA (Fig 2B). Our results demonstrate that these miRNAs were differentially expressed between male and female schistosomes (Fig 2B). We noted that the remaining 20 *S*. *japonicum* miRNAs were differentially expressed during schistosome development but showed no significant differential expression between males and females (Fig 2A). We used stem-loop based qRT-PCR to further validate the expression of these miRNAs during schistosome development (Fig 2C and S4 Table). The qRT-PCR data demonstrated these miRNAs were differentially expressed during schistosome development and that the expression data from qRT-PCR and deep sequencing in general correlated well (Fig 2C).

### Identification of miRNA targets

We predicted potential target mRNAs for these miRNAs using RNAhybrid software. To corroborate these predictions, we used antibodies against *S*. *japonicum* Argonaute proteins [60,63,64] (see Materials and Methods) to pull-down Argonaute miRNA/mRNAs complexes from paired male and female schistosome extracts and examined the accuracy of the mRNA target predictions (S3 Fig). RNA was isolated from Ago pull-downs, reverse transcribed, amplified by PCR, the PCR products cloned, recombinant clones randomly selected, and then sequenced (S3, S4A and S4B and S5 Figs and S5 Table) to determine if the predicted mRNA targets were enriched in the Argonaute pull-downs. The Argonaute enriched mRNA sequences were first used to identify the full-length mRNAs in NCBI database using BLAST, and then analyzed using RNAhybrid to predict miRNA:mRNA pairs. The overlapping mRNAs between the two methods were considered to be probable target mRNAs (Table 2 and S6 Table). Overall, this approach led to the corroboration of 30 potential target mRNAs for 16 of the described miRNAs.

**Table 2:** Validated target genes for *S*. *japonicum* miRNAs.

miRNAs	Target gene IDs	Name of target genes	MiRNA:mRNA duplex*	Mfe (kcal/mol)	Validated^#^
Bantam	AY223092.1	Serine-arginine repressor	Target 5`U CUUU A U 3`	-22.5	By miRNA suppression
			GGCU AUC CGAUCUCG		
			UCGA UAG GCUAGAGU		
			miRNA 3`AAU C 5`		
	FN323394.1	FUS-interacting serine-arginine-rich protein 1	Target 5`G UU A U 3`	-22.6	By luciferase assay/ miRNA suppression
			GGUUUU AUC CGAUCUCG		
			UCGAAA UAG GCUAGAGU		
			miRNA 3`U C 5`		
	AY815078.1	Smad1	Target 5`U UG C 3`	-19.9	By miRNA suppression
			GGC UUGAUCGUG CUUA		
			UCG AAUUAGCGC GAGU		
			miRNA 3`A UA 5`		
Let-7	FN314191.1	Ribosomal protein S6 kinase 2	Target 5`A C U A 3`	-28.6	By luciferase assay
			CAU CAAC GAACUACCUC		
			GUG GUUG CUUGAUGGAG		
			miRNA 3`UG U G5`		
miR-2a	FN321618.1	Plasminogen activator inhibitor 1	Target 5`A AG CUGAAU C3`	-24.7	By luciferase assay
			GUUCA GA UUGGCUGUG		
			CAAGU UU GACCGACAC		
			miRNA 3`G AG AU U5`		
miR-31	EU370927	Frizz7	Target 5`G G G 3`	-22.0	By luciferase assay
			UCGUCGUGGUU UUGU		
			AGCGGCAUUAG AACG		
			miRNA 3`UCGA GU5`		
	FN319623.1	O-glycosyltransferase	Target 5`U C 3`	-28	By miRNA suppression
			GCUUU UUGUAAUCUUGCC		
			CGAAG GGCAUUAGAACGG		
			miRNA 3`U C U5`		
miR-1989	FN317226	Asparagine-rich protein	Target 5`A U AUA UA U 3`	-26.4	By luciferase assay
			C AAGA GUGA ACACAGUUGA		
			G UUCU UACU UGUGUCGACU		
			miRNA 3`A C G		
miR-8	FN313640	Integral membrane protein GPR177	Target 5`U AC UUAUUU G3`	-22.3	By luciferase assay
			GCAUUUU UACCUA AUAGUA		
			CGUAGAA AUGGAU UGUCAU		
			miRNA 3`C AAU5`		
	DQ643829.2	Wnt	Target 5`C A 3`	-20.8	By luciferase assay
			GGCAUC ACC GC GUAUUA		
			CCGUAG UGG UG CAUAAU		
			miRNA 3`AAA AU U 5`		
miR-3479	FJ753578.1	Transforming growth factor receptor II	Target 5`A U ACUUA U3`	-20.3	By luciferase assay
			CGA GC UAAGUGCAAUA		
			GUU CG AUUCACGUUAU		
			miRNA 3`C CUUCC 5`		

*miRNA:mRNA pair analysis was performed using RNAhybrid (http://bibiserv.techfak.uni-bielefeld.de/rnahybrid/)

^#^luciferase assay = miRNA mimics down-regulate target mRNA sequences in mammalian cells; miRNA suppression = transfection of antisense miRNA sequences into schistosomes leads to increases in target mRNAs

### Validation of miRNA targets

**Fig 3:**
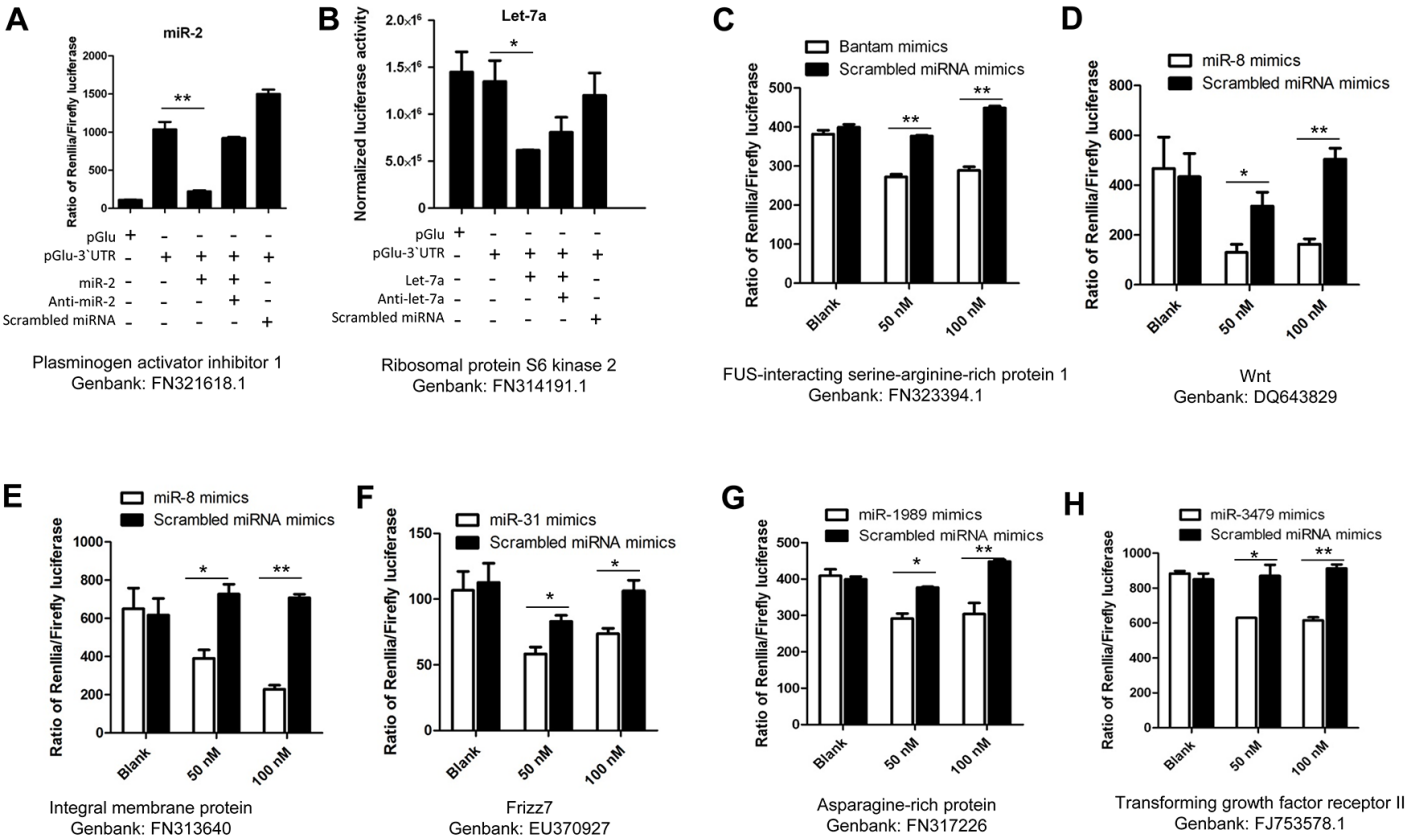
Verification of predicted schistosome miRNA targets in mammalian cells. Transfection of miR-2 (A), Let-7a (B), bantam (C), miR-8 (D and E), miR-31 (F), miR-1989 (G), and miR-3479 mimics (H) into HEK293T or Hela cells led to a significant reduction of luciferase activity from co-transfected plasmids containing their corresponding target regions. Hela or 293T cells were transfected with a recombinant Gaussia luciferase plasmid (pGLU-CMV) containing the corresponding *S*. *japonicum* cDNA fragment of a predicted mRNA targeted by a miRNA. These cells were simultaneously co-transfected with a control firefly luciferase plasmid. At 24 h post-transfection, a control miRNA or miRNA mimics were transfected into these cells. At 24–48 h post-transfection, luciferase activity was analyzed using a dual-luciferase reporter assay system with normalization to either firefly luciferase levels or protein concentration. For A and B, all the samples within the assay are illustrated, whereas in C-H only the effects of the miRNA mimics and scrambled miRNA mimics are shown. Similar results as illustrated in A and B were observed for the experiments in C-H. Blank represents no miRNA applied. Each experiment shows representative results and illustrates the mean and standard errors derived from triplicate experiments. * means P ≤ 0.05 and ** means P ≤ 0.01 (student’s t test, miRNA mimics treatment vs scrambled miRNA mimics treatment).

To validate the potential miRNA targets identified above, we determined whether miRNA mimics could repress luciferase mRNAs containing the target schistosome mRNA regions in mammalian cells. The target regions of potential miRNA binding sites for 9 predicted target mRNAs were selected and cloned into the 3’ UTR of a luciferase reporter vector (pGLU-CMV) (Fig 3 and S7 Table). Hela or HEK293T cells were transfected with these recombinant plasmids, control plasmids (pGL3), and the corresponding 2`-O-methyl and phosphorothioate miRNA mimics representing bantam, miR-8, miR-31, miR-1989, miR-3479, or control mimics (scrambled or mismatched seeding region of these miRNA) (S8 Table). As shown in Fig 3, transfection of bantam and miR-31 miRNA mimics resulted in a reduction of luciferase activity compared to transfection with scrambled miRNA mimics, indicating that these miRNAs can down-regulate the expression of the corresponding schistosome mRNA target regions in a heterologous system. Similar results were also observed in cells transfected with miR-8, miR-3479 and miR-1989 mimics and the corresponding recombinant plasmids expressing schistosome mRNA target sites (Fig 3). Furthermore, co-transfection in these experiments with antisense miR-mimics (miR-2 and let-7a) led to de-repression of luciferase (Fig 3A and 3B). We verified 8 target mRNAs for 7 miRNAs. Only one target mRNA for miR-277 failed to be validated in the luciferase assays. Overall, these data demonstrate the accuracy of our target mRNA predictions, indicate that schistosome miRNA sequences can effectively repress specific mRNA targets in mammalian cells, and that these miRNAs are likely to target these mRNAs in schistosomes.

**Fig 4:**
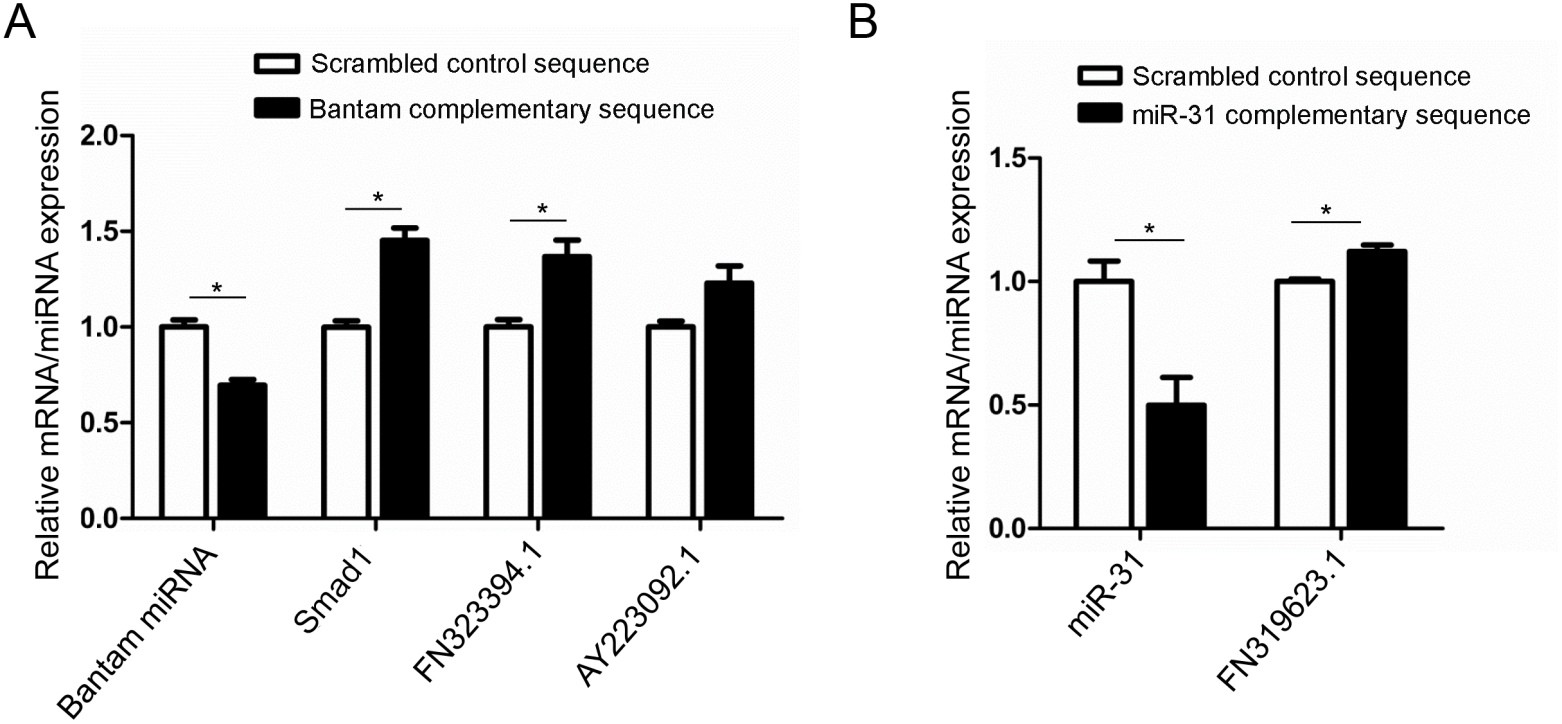
*In vivo* de-repression of *S*. *japonicum* target mRNAs with antisense miRNAs. (A) Effect of bantam suppression on the expression of its putative target genes. (B) Effect of miR-31 suppression on the expression of its putative target gene. Adult schistosomes were collected from *S*. *japonicum* infected mice at 26–28 days post-infection. The female schistosomes were electroporated with anti-miRNAs or scrambled anti-miRNAs and their effects on the levels of endogenous miRNA and predicted target mRNAs determined by qRT-PCR at 4 days post-electroporation. Data illustrate representative results with the mean and standard error derived from triplicate experiments. * means P ≤ 0.05 and ** means P ≤ 0.01 (student’s t test, miRNA inhibitor treatment vs scrambled miRNA inhibitor treatment).

To determine whether these miRNAs could repress mRNA targets *in vivo* in schistosomes, we introduced bantam and miR-31 antisense miRNAs by electroporation into *in vitro* cultured adult female schistosomes and determined if their target mRNA levels changed using qRT-PCR. As shown in Fig 4A, *in vivo* suppression of bantam resulted in an increase in all three putative target mRNAs (GenBank accession numbers: AY815078, AY223092.1, and FN323394.1) including a target that was independently verified in our mammalian cell assays (S9 Table). Similar results were also observed in the schistosomes treated with antisense miR-31 (Fig 4B). Although another important evaluation for these *in vivo* assays would be to measure proteins translated from the target mRNAs, antibodies to these proteins are currently not available. Overall, we verified 11 mRNA targets for 7 of the miRNAs using either luciferase assay or *in vivo* miRNA suppression experiments (Table 2). Our *in vitro* luciferase assay in mammalian cells and *in vivo* schistosome data demonstrate that these schistosome miRNAs can regulate the expression of the predicted mRNA targets.

### Effect of miRNA suppression on schistosome ovary architecture

**Fig 5:**
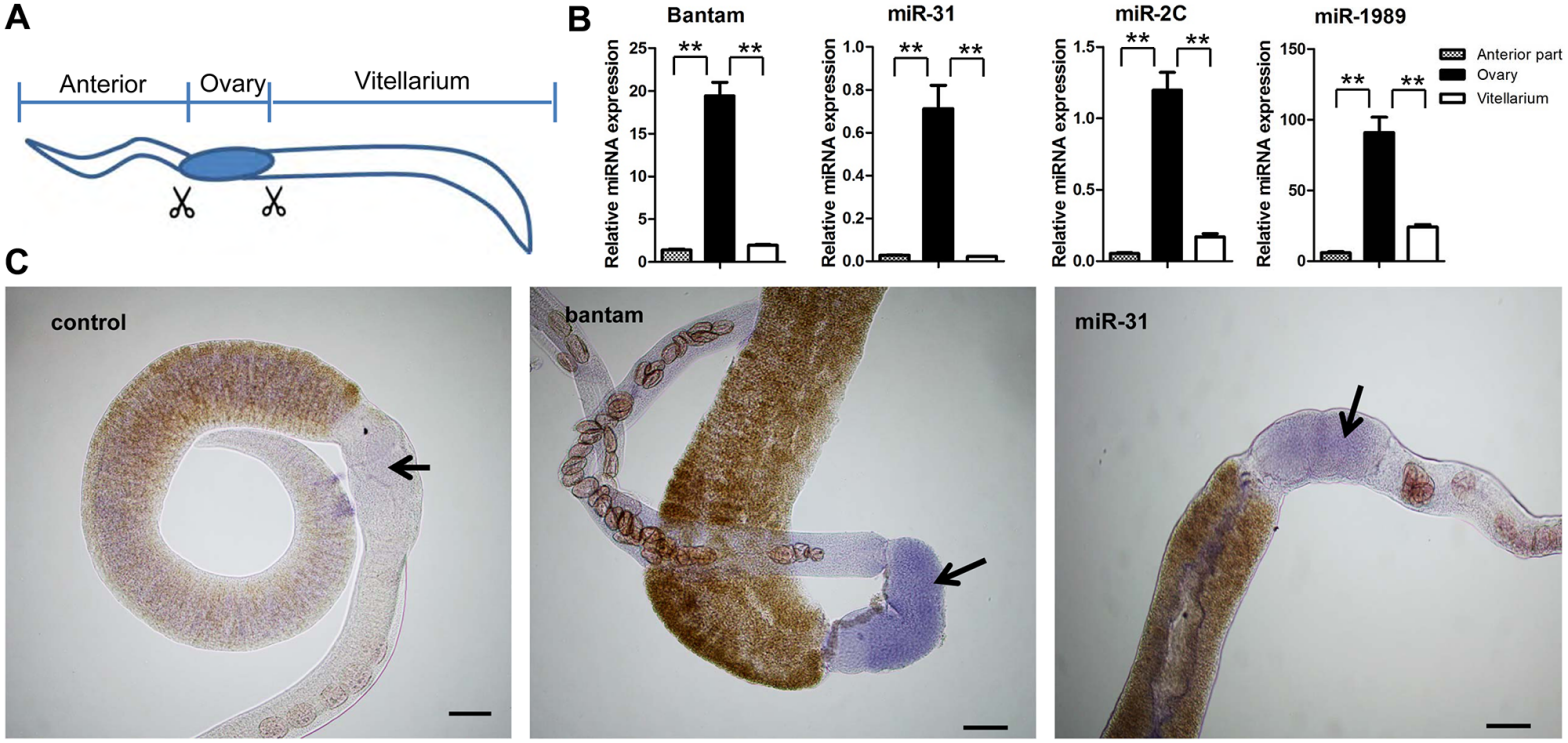
Female enriched *S*. *japonicum* miRNAs are predominantly expressed in the ovary. (A) Schematic diagram of the dissection of female schistosomes. (B) qRT-PCR analyses for the expression of four female enriched miRNAs (miR-31, bantam, miR-1989, miR-2c). * means P ≤ 0.05 and ** means P ≤ 0.01 (student’s t test, ovary vs. anterior and ovary vs. vitellarium). Data illustrate representative findings and show the mean and standard errors derived from triplicate experiments. (C) *In situ* hybridization experiments were carried out using a labeled locked nucleic acid (LNA) complementary to miR-31 or bantam miRNA. A labeled scrambled LNA was used as a control. Bars indicate 100 μm.

We reasoned that the female-enriched miRNAs might play an important role in *S*. *japonicum* sexual maturation and egg production. We dissected females into three parts (anterior, ovary, and vitellarium) and used qRT-PCR to examine the expression of the miRNAs in the ovary. Four female enriched miRNAs (miR-31, bantam, miR-1989 and miR-2c) were shown to be predominantly expressed in the region of ovary (Fig 5A and 5B). To further corroborate these results, we selected two of the female enriched miRNAs, bantam and miR-31, and used *in situ* hybridization to demonstrate that they predominantly localize to the ovary (Fig 5C and S10 Table). We next demonstrated that the target mRNAs of bantam (Smad1) or miR-31 (Frizz7) are also primarily localized in ovary of female schistosomes (S6 Fig and S10 Table). The co-localization of the miRNAs and their target mRNAs, at least for bantam miRNA and miR-31, suggest their coordinated involvement in the regulation of ovarian development.

**Fig 6:**
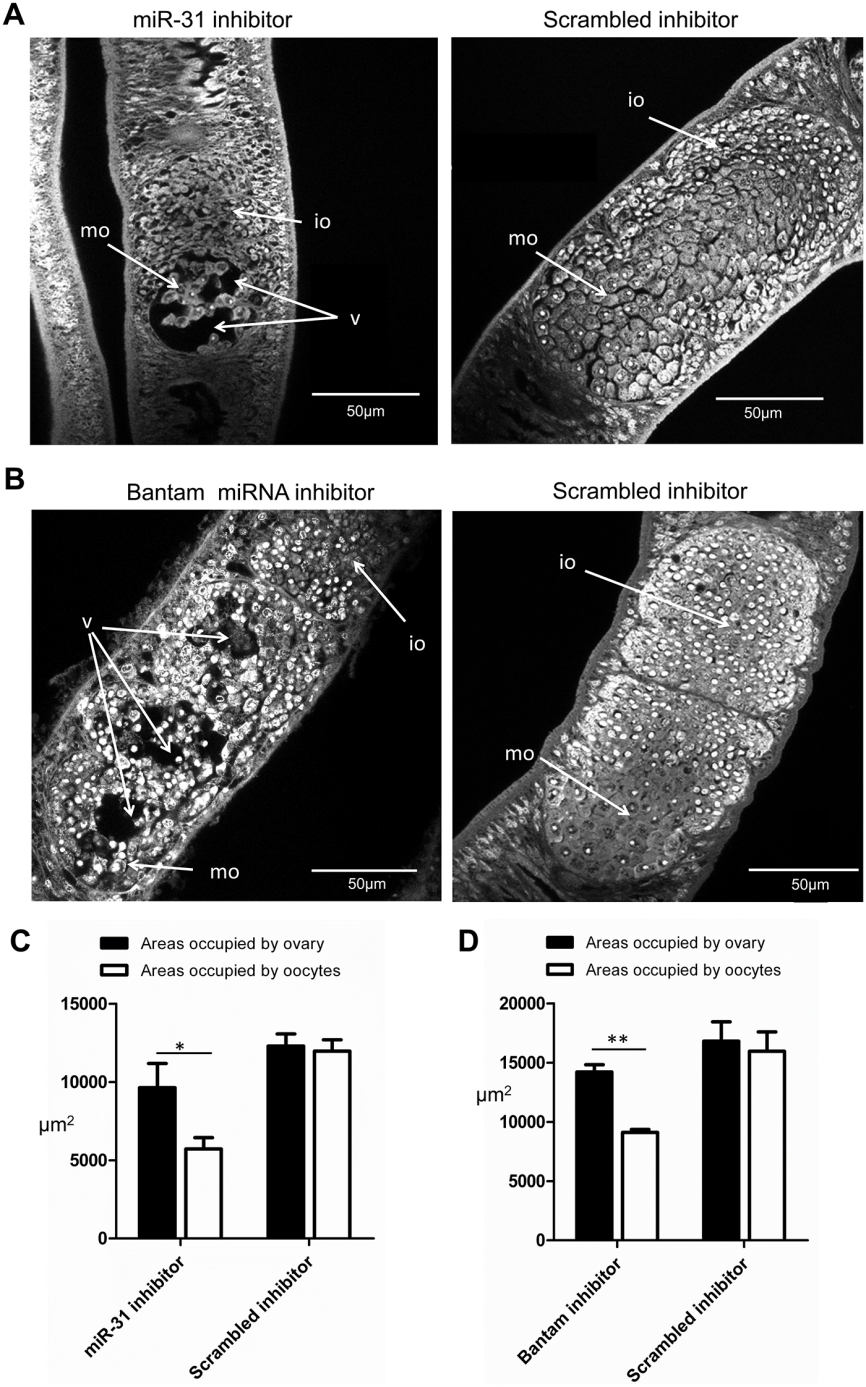
**Effect of miR-31 (A) or bantam (B) suppression on female ovary architecture and morphology.** Female schistosomes were electroporated either with miRNA inhibitors (antisense miRNA) (3 μg) or scrambled miRNA inhibitors (3 μg) and then incubated for 4 days as described in Materials and Methods. *S*. *japonicum* worms were stained with carmine red and whole-mount preparations of worms were imaged by confocal microscopy. Io, immature oocyte; mo, mature oocyte; v, vacuole. Quantitation of ovary changes due to miR-31 (C) and bantam miRNA suppression (D). The ovary defects were analyzed using ImageJ as described in Materials and Methods based on a comparison of the area occupied by the ovary and oocytes and the appearance of oocytes in the ovaries. Data show the mean and standard errors derived from four randomly selected female worms. * means P ≤ 0.05 and ** means P ≤ 0.01 (student’s t test, ovary areas vs areas of appearance of oocytes in ovary).

To test whether miR-31 and bantam miRNA suppression would lead to any morphological changes in female schistosomes, 24–28 day-old female worms were cultured *in vitro* for four days following electroporation of antisense miR-31 or bantam into worms. As controls, scrambled antisense miRNA were introduced into female worms. Upon transfection of 8–10 females with each miRNA inhibitor in a cuvette, we observed that at least 70% of the worms had significant defects in the ovarian architecture by confocal microscopy. Within the *S*. *japonicum* female ovary, immature oocytes mature into well-differentiated primary oocytes along an anterior-to-posterior gradient. We observed that there were gross-morphological defects for ovarian architecture in most of the treated females that included the formation of numerous vacuoles and the appearance of damaged oocytes (Figs 6A and 6B and S7–S10 and S1–S4 Movies). By examining the ratio of the total area of ovary to the area occupied by oocytes, miR-31/bantam inhibitor resulted in a significant reduction of parenchymal cells and oocytes in the ovary of female schistosomes (Fig 6C and 6D). Morphological changes in other female tissues were not observed, and no alterations were found within the ovaries of females treated with control antisense miRNA. These results suggest that bantam and miR-31 and their target mRNAs play important roles in the development of the structure and architecture of the ovary and in oocyte differentiation. We did not observe significant changes on other physiological parameters such as behavior, viability or mortality rates of target/antisense RNA-treated females compared with controls females treated with scrambled antisense miRNA (S11 Fig). Furthermore, RNA levels of miR-31 and bantam determined by qRT-PCR were also significantly reduced in target/antisense RNA-treated worms compared to control worms (S12 Fig).

## Discussion

A number of studies have investigated the molecular basis for schistosome egg production by determining and examining genes associated with reproductive development [8–10,12,13,21,65–71]. Although schistosome miRNAs have been identified in several different stages of *S*. *mansoni* [48–51,72] and *S*. *japonicum* [52–54] (including cercariae [45], lung-stage schistosomula [45], hepatic-stage schistosomula [45,46,52,53], adult males and females [54], and eggs [44]), the potential regulatory roles of small RNAs in schistosomes, and particularly in sexual maturation and egg production have not been previously investigated. Our study represents the first attempt to systemically characterize *S*. *japonicum* miRNAs associated with sexual maturation and egg production, to predict potential mRNA targets, to test these predictions *in vitro* and *in vivo*, and to determine whether repression of miRNAs has a consequence on the female reproductive system.

We identified 28 known miRNAs and 10 novel miRNAs associated with sexual maturation and egg production in *S*. *japonicum*. The 28 known miRNAs were previously reported to be expressed in other schistosome stages including eggs isolated from the host’s liver, cercariae and lung schistosomula [44,45], suggesting that these miRNAs likely play diverse roles in schistosomes. Among the 10 novel miRNAs, three of the miRNAs (let-7b, let-7s, and miR-750) have long hairpin structures (>200 nt), suggesting that schistosomes miRNA biogenesis has adapted to process these long hairpin precursor miRNAs. Three other novel miRNAs (lin-4, miR-1b and miR-277b) are significantly expressed (>20,000 reads in the libraries) and conserved in other metazoan although the current draft *S*. *japonicum* genome data lacks the predicted hairpin structures. Our Northern blot and Semi-quantitative RT-PCR results clearly demonstrated that the miRNA are expressed in *S*. *japonicum*.

Male-female pairing is essential for female sexual maturation. It has been suggested that the male schistosomes ensure physical transport, correct tissue localization, aid feeding, and provide maturation factors for female development and egg production [6–9,12,13]. We identified 14 male-enriched miRNAs and 4 female-enriched miRNAs using high-throughput sequencing, and their expression was independently verified by qRT-PCR. Several sex-enriched miRNAs were also previously identified as differentially expressed including sja-miR-7, sja-miR-61, and sja-miR-219 in male worms and sja-bantam in female worms [45,50]. We identified several target genes of miR-219 and bantam miRNA using an Argonaute antibody-based pull-down assay in combination with bioinformatic analyses. Furthermore, using antisense RNA to inhibit miRNAs, we demonstrated that bantam miRNA plays a regulatory role in ovary development and oocyte maturation. We further identified 30 potential target mRNAs for 16 *S*. *japonicum* miRNAs. Eleven of the targeted mRNAs for 7 of the miRNAs were further validated using an *in vitro* dual-luciferase reporter assay or *in vivo* miRNA suppression experiments (Figs 3 and 4 and Table 2). These data provide the first experimental demonstration of target prediction and verification both *in vitro* and *in vivo* in schistosomes.

Among the validated miRNA targets, we found that male enriched miR-8 and miR-3479 regulate the molecules likely involved in the Wnt (GenBank Accession Nos: FN313640 and DQ643829.2) and TGF-β (Accession No. FJ753578.1) signaling pathways. These pathways have been shown to be involved in the regulation of schistosome development [20,73] and embryogenesis [66,67,74]. Consequently, our results suggest that miRNAs may contribute to the regulation of Wnt and TGF-βpathways influencing the physiological processes associated with their activities [41,42].

We demonstrated that female-enriched miR-31 can regulate the mRNA for Frizz 7 (Accession No: EU370927, a receptor of WNT signaling pathway) and an O-glycosyltransferase (Accession No: FN319623.1) whereas the female-enriched miR-1989 likely interacts with asparagine-rich protein mRNA (Accession No. FN317226). As a member of the frizzled family of G protein-coupled receptors, Frizz 7 is a transmembrane receptor involved in multiple signal transduction pathways, particularly in modulating the activity of the Wnt proteins, which play a fundamental role in the early development of metazoa [75]. Asparagine-rich proteins are involved in the transcriptional regulation of key eukaryotic developmental processes [76] and O-glycosyltransferase is a catalytic enzyme in glucose metabolism involved in the transfer of activated carbohydrate moieties from donor molecules (e.g. UDP-galactose) to an acceptor molecule associated with the regulation of carbohydrate metabolism and/or other functions [77,78]. We also observed that bantam miRNA suppression leads to a significant increase in mRNA levels of three other identified targets, including a serine-arginine repressor (Accession No. AY223092.1), FUS-interacting serine-arginine-rich protein 1 (Accession No. FN323394.1), and Smad1. Serine-arginine repressor and FUS-interacting serine-arginine-rich protein 1 are members of the serine-arginine family of proteins involved in constitutive and regulated RNA splicing [79,80]. Moreover, studies in Drosophila [81] indicated that bantam was an effector of several signaling pathways such as Hippo [82,83], Notch [84], Dpp [85], and epidermal growth factor receptor (EGFR) [86] during fly development. We demonstrated in a previous study that *S*. *japonicum* bantam can be detected in the circulation of the definitive host [87], suggesting that bantam might be a secretory miRNA that has the potential to act as a signaling molecule for other schistosomes or even host cells [56]. Collectively, these results suggest that female enriched miRNAs may be involved in a variety of processes including signal transduction for schistosome sexual maturation and egg production.

miR-31 and bantam are predominantly present in the ovaries of female schistosomes (Fig 5), suggesting that these miRNAs may play an important role in ovary development. Suppression of these miRNAs led to significant defects in the ovaries. These findings strongly imply that female enriched miRNAs such as bantam and miR-31 may be key regulators in *S*. *japonicum* ovarian development. We also demonstrated that schistosome smad1 (a target gene of bantam) and Frizz7 (a target gene of miR-31) are predominantly localized in the ovary of *S*. *japonicum*, consistent with their localization in *S*. *mansoni* [88–90]. From these data, we conclude that bantam and miR-31 are involved in the regulation of target genes that are instrumental in ovary development and oocyte maturation. Overall, our data identify female enriched miRNAs and their mRNA targets that play important roles in sexual maturation in *S*. *japonicum*.

Taken together, our study represents the first attempt to systemically characterize *S*. *japonicum* miRNAs associated with sexual maturation and egg production by predicting miRNA targets and then using both *in vitro* and *in vivo* assays to functionally evaluate the predictions and examine the roles of the miRNAs *in vivo*. Importantly, our findings provide the first functional evidence for the role of miRNAs acting as important regulators in ovary development and sexual maturation in *S*. *japonicum*. Since schistosome eggs are the major cause of schistosome pathology and play a key role in the epidemiology of human infection, our studies have important implications for human schistosomiasis.

## Materials and Methods

### Ethics statement

All experiments involving mice and rabbits were carried out in strict accordance with the recommendations in the Guide for the Care and Use of Laboratory Animals of the Ministry of Science and Technology of the People's Republic of China, and all efforts were made to minimize suffering. All animal procedures were approved by the Institutional Animal Care and Use Committee (IACUC) of the Shanghai Veterinary Research Institute, Chinese Academy of Agricultural Sciences (Permit Number: SHVRIAU-10-0101).

### Animals and parasites

The life cycle of *S*. *japonicum* (Anhui isolate) was maintained in New Zealand rabbits and BALB/c mice using *Oncomelania hupensis* as the snail host. Unless indicated otherwise, New Zealand rabbits and BALB/c mice were infected with approximately 1,000 and 100 cercariae, respectively, by inoculating the shaved abdominal skin surface with a moist cercarial paste. To collect schistosomes at different developmental stages, the parasites were perfused from BALB/c mice every day starting at 14 days post infection through 35 days post-infection. Males and females were manually separated and pooled. Schistosomes at the developmental stages of pairing, gametogenesis, and egg production were collected from infected rabbits at 16, 22, or 28 days post-infection, respectively. Males and females were manually separated. The sexes of worms were confirmed by microscopic analysis. The parasites were snap-frozen and stored in liquid nitrogen until use for total RNA isolation.

### RNA isolation

Total RNA was extracted from *S*. *japonicum* samples using TRIzol reagent (Invitrogen) according to the manufacturer's protocol. Samples included 16-day-old males (16M), 16-day-old females (16F), 22-day-old males (22M), 22-day-old females (22F), 28-day-old males (28M), 28-day-old females (28F), a male mixture (M) (pooled from 14–35 days), and a female mixture (F) (pooled from 14–35 days). To maximize precipitation of small RNAs, RNA precipitation with isopropanol was carried out overnight at -80°C. RNA was quantified using a Nanodrop ND-1000 spectrophotometer (Nanodrop Technologies, Wilmington, DE), and RNA quality was evaluated using an Agilent 2100 Bioanalyzer (Agilent Technologies).

### Small RNA libraries, sequencing, and bioinformatics

Small RNA libraries, sequencing, and bioinformatics are described in Supplemental materials and methods.

### Northern blot and real time RT-PCR analyses of schistosome miRNAs

DNA oligonucleotides complementary to miRNA sequences were end-labeled with DIG (Invitrogen, Shanghai) at their 5’ termini and used as probes (S3 Table). Northern blot analyses were performed according to a previously described method [91] using 30 μg of total RNA isolated from adult male and female schistosomes isolated from BALB/c mice at 26 days post-infection.

For real time RT-PCR analyses of miRNA expression, schistosomes were collected from rabbits infected after 16, 22, and 28 days post-infection. Male and female worms were manually separated. Total schistosome RNA was isolated TRIzol (Invitrogen) from different developmental stages, sexes, and regions of female worms. Real-time RT-PCR was performed as described previously [87] (see S1 File). To examine miRNA expression in different regions of females, 28 day females were dissected into three parts including an anterior, ovary, and vitellarium. The miScript primers were Qiagen's proprietary primers. For semi-quantitative RT-PCR or stem-loop based qRT-PCR, a stem-loop based RT-PCR method was used to analyze miRNA expression (see S1 File). A stem-loop RT primer was used to reverse-transcribe mature miRNAs to cDNAs. Then, PCR was performed for miRNA abundance analysis. For semi-quantitative RT-PCR, the PCR products were analyzed on 2% agarose gels and images were captured under a UV light using a digital camera. The primers used for stem-loop based qRT-PCR and semi-quantitative RT-PCR are listed in S4 Table.

### Argonaute antibody-based pull-down assay

Adult paired schistosomes (~150 mg, 28 day post-infection) were lysed in 500 μL lysis buffer containing 25 mM Tris (pH 7.4), 150 mM KCl, 0.5% NP-40, 2 mM EDTA, 1 M NaF, 0.5 M DTT and protease inhibitors (Roche) and were centrifuged at 10,000×g for 10 min at 4°C. The pull down assay was performed using a Dynabeads Protein G immunoprecipitation Kit (Invitrogen) according to the manufacturer’s instructions. We used three previously developed polyclonal antibodies to the three *S*. *japonicum* Ago proteins [60] and combined them for the pull-down assay. Briefly, approximate 25 mL of each anti-Ago serum was coupled to 1.5 mg Dynabeads. Then, 200 μL of worm lysate was incubated with the antiserum-coupled beads for 20 min at room temperature, and the Dynabeads were washed three times in 200 μL wash buffer provided by the kit. Finally, target antigens were eluted with the kit elution buffer. The elution was analyzed by SDS-PAGE electrophoresis and sliver staining as described in our previous study [10]. Western blot analyses were used to confirm the immunoprecipitation (IP) results.

### Isolation of co-precipitated RNA and PCR amplification

RNA was isolated from the Argonuate IPs using TRIzol LS (Invitrogen) as described above, and the isolated RNA was reverse transcribed using Superscript II (Invitrogen) and the primer 5’ GCT GTC AAC GAT ACG CTA CGT AAC GGC ATG ACA GTG TTT TTT TTT TTT TTT TTT TTNN 3’, where N represents the random nucleotides G/C/A. The cDNAs were amplified using a specific forward primer designed for each miRNA seed sequence and 0.5 μM universal reverse primer (S5 Table), the PCR products were resolved on a 1.5% agarose gel, purified using an AxyPrep 96 PCR Clean Kit (Axygen), and cloned into pMD19-T vector (Takara). Over twenty recombinant clones for each miRNA were randomly selected for sequencing. To determine the enrichment of mRNA targets in the Argonaute pull-downs, five selected targets (GenBank accession numbers: AY233092.1, FN319623.1, FN32339.4, EU370927, AY815078.1) were evaluated by comparing the abundance of the RNA in Argonaute pulled down in the IPs to an IP control, total RNAs isolated from protein lysates, and the supernatant.

### Bioinformatic analyses for miRNA targets

*In silico* prediction of miRNA targets was performed using RNAhybrid software (-b 1 -m 100000 -v 3 -u 3 -s 3utr_worm -e -19 -t gene.fa -q miRNA.fa). The sequences of the PCR products from the Argonaute IP analyses were characterized by using BLAST against *S*. *japonicum* nucleotide sequences in the NCBI GenBank collection (http://www.ncbi.nlm.nih.gov) and an E-value cutoff < 1e-20. Only the sequences annotated as protein coding were further characterized by miRNA:mRNA pairwise analyses using the online RNAhybrid program (http://bibiserv.techfak.uni-bielefeld.de/rnahybrid/). A miRNA:mRNA duplex with a minimum free energy (MFE) < = -19 kcal/mole was considered as a probable miRNA/mRNA target pair.

### Vector construction

cDNA fragments corresponding to regions complementary to miRNAs (target genes) were PCR-amplified from *S*. *japonicum* cDNA and the PCR products were cloned into pCMV-Glu vector (Targeting Systems, USA) using standard molecular cloning methods. The primer pairs used for PCR amplification and the restriction enzyme sites are listed in S7 Table. The recombinant plasmids were confirmed by sequence analysis.

### Cell transfection

HEK293T cells or Hela cells were cultured in 48 well plates containing Dulbecco’s modified Eagle’s medium (Invitrogen) with 10% fetal bovine serum (Hyclone), 100 units/mL penicillin, and 100 μg/mL streptomycin (Invitrogen) and incubated at 37°C in 5% CO_2_. Recombinant and control plasmids were transfected at 0.5 ng to 1.5 ng per well into cultured cells using Lipofectamine 2000 (Invitrogen) along with pGL3 (40 ng) for normalization. At 24h post transfection, the cells were further transfected with a miRNA mimic, miRNA inhibitor, or irrelevant miRNA (S8 Table) at the indicated concentrations using Lipofectamine 2000 (Invitrogen). The miRNA mimics, ant-sense miRNAs or scrambled miRNAs used were modified by 2`-O-methyl or 2`-O-methyl and phosphorothioate. The transfected cells were incubated for the indicated time prior to collection for dual luciferase assay as described below. Transfections were carried out in triplicate at least six times using two independent plasmid preparations.

### Luciferase reporter assay

At 24–48 h post-transfection, luciferase activity was analyzed using a dual-luciferase reporter assay system (Promega) according to the manufacturer’s instructions. Relative reporter activity for transfected cells was obtained by normalization to either co-transfected firefly luciferase activity (pGl-3) or protein concentration using the Pierce BCA protein assay kit and Compat-Able protein assay preparation reagent set (Pierce).

### *In situ* hybridization

In situ hybridization for determining miRNA and mRNA localization in *S*. *japonicum* was performed according to the method for *S*. *mansoni* WISH analysis with modifications [92]. The method is further described in S1 File and the LNA probes and the primers for probe preparation are listed in S10 Table.

### Schistosome culture and electroporation

*S*. *japonicum* were collected from mice 24–28 days post-infection, females were separated and cultured in a 12-well flat bottom plate containing 2 mL complete RPMI-1640 media supplemented with 2 g/L glucose, 0.3 g/L l-glutamine, 2.0 g/L NaHCO3, 15% fetal bovine serum (heat inactivated), and 5% pen/strep (10,000 units penicillin and 10 mg/streptomycin in 0.9% NaCl) in a humidified 5% CO_2_ chamber at 37°C. miRNA inhibitors (antisense miRNA) and scrambled miRNA inhibitors (3 μg per experiment, chemically synthesized in Shanghai GenePharma, China) (S8 Table) were electroporated (125 V, 20 ms, 1 pulse in 200 μL RPMI 1640 media) into cultured schistosomes. Schistosomes were then transferred into a 12-well cell culture plates containing 2 mL fresh media. Worm mobility and survival were observed under an inverted microscope (Olympus, Japan) at the indicated times. The parasites were collected at 96 hr post electroporation for qRT-PCR analysis and confocal microscopy as described below.

### qRT-PCR analysis of target gene transcripts in electroporated schistosomes

At 96 h post electroporation, RNA was isolated from worms using RNAiso plus (Takara) according to the manufacturer’s protocol except for an extended overnight precipitation in isopropanol at -80°C. First strand cDNA was produced from 100 ng RNA using a PrimeScript 1st Strand cDNA Synthesis Kit (Takara) with either an miRNA specific primer (2 μM, 0.5 μL) or random primer combined with Oligo d(T) as described above. PCR was then carried out using primers described in S9 Table. *S*. *japonicum NADH* (forward primer: CGA GGA CCT AAC AGC AGA GG; reverse primer: TCC GAA CGA ACT TTG AAT CC) was used as an internal control. The 2^-ΔCt^ method was used to calculate relative expression [62].

### Confocal microscopy of schistosome morphology

At 96 h post electroporation, the worms were preserved, stained, and mounted as described previously [93] and subjected to morphological examination using confocal microscopy (Nikon, Japan). Briefly, the worms were fixed in formalin (10%), alcohol (48%) and glacial acetic acid (2%), stained with carmine red, cleared in 0.5% hydrochloric alcohol solution, and preserved as whole mounts. Images were obtained with a Nikon CLSI laser confocal microscope (Nikon, Japan), using a 488 nm He/Ne laser. Z-Stacks were transformed into movies. Images in the Z-stacks was selected for pixel value analysis using ImageJ [94]. At least four female schistosomes were randomly selected for ImageJ analyses.

## Supporting Information

S1 Fig Schistosome development and sexual maturation. Pairing of *S*. *japonicum* males and females usually occurs 15–18 days post-infection in the mammalian host, gametogenesis begins at 19–21 days post-infection, and male and female schistosomes begin to produce mature gametes at 22 days post-infection.
(PDF)

S2 Fig Classification and identification of *S*. *japonicum* small RNAs. (A) Classification of small RNAs in 16-day old females (16F), 16-day old males (16M), 22-day old females (22F), 22-day old males (22M), 28-day old females (28F), 28-day old males (28M), and mixed males (M) and mixed females (F). (B) Size distribution of small RNAs in different *S*. *japonicum* stages. (C) Classification and percentage of *S*. *japonicum* small RNAs from different stages and sexes. Unannotated = small RNAs that map to the genome, but the genome regions are not annotated.
(PDF)

S3 Fig Argonaute antibody-based pull down assay for identifying *S*. *japonicum* miRNA targets. (A) Analysis of pull down products based on SDS-PAGE and silver staining. (B) Western blot analysis of the pull-downs.
(PDF)

S4 Fig RT-PCR analysis of the Argonaute antibody-based pull-down assay. (PDF)

S5 Fig qPCR validation of mRNA targets enriched in the pull-down assay. (PDF)

S6 Fig *In situ* hybridization analyses of the localization of Smad1 and Frizz7 in *S*. *japonicum* females. Arrows indicate ovary in *S*. *japonicum*. Bars indicate 100 μm.
(PDF)

S7 Fig Z-Stack of optical sections from *S*. *japonicum* ovary treated with the miR-31 inhibitor. (PDF)

S8 Fig Z-Stack of optical sections from *S*. *japonicum* ovary treated with a scrambled miR-31 inhibitor. (PDF)

S9 Fig Z-Stack of optical sections from *S*. *japonicum* ovary treated with bantam miRNA inhibitor. (PDF)

S10 Fig Z-Stack of optical sections from *S*. *japonicum* ovary treated with scrambled bantam miRNA inhibitor. (PDF)

S11 Fig Effect of miRNA suppression on worm mortality in female schistosomes treated with antisense miRNAs. (A) Effect of miR-31 suppression on worm mortality in female schistosomes. (B) Effect of bantam suppression on worm mortality in female schistosomes. Data illustrate the mean and standard error derived from triplicate experiments including at least 30 female schistosomes.
(PDF)

S12 Fig qRT-PCR analyses of the expression of miR-31 (A) or bantam miRNA (B) in female schistosomes treated with miRNA inhibitor. The female schistosomes were electroporated with anti-miRNAs or scrambled anti-miRNAs and their effects on the levels of endogenous miRNA was determined by qRT-PCR at 4 days of post-electroporation. Data illustrate the mean and standard error derived from triplicate experiments. * means P ≤ 0.05 (student’s t test, miRNA inhibitor treatment vs scrambled inhibitor treatment).
(PDF)

S13 Fig Alignment of small RNAs to miRNA hairpins. The length and read number of the small RNA are indicated at the end of each small RNA sequence. The reads numbers are the sum of small RNA reads in all of the 8 libraries.
(PDF)

S1 Table Schistosome small RNA sequences. (PDF)

S2 Table miRNAs identified in *Schistosoma japonicum* and their normalized levels in different stages and sexes. (XLSX)

S3 Table Oligonucleotides used as probes for Northern blots. (PDF)

S4 Table Primers for stem-loop RT-PCR analyses. (PDF)

S5 Table Primers used for miRNA target identification. (PDF)

S6 Table Potential target genes for *S*. *japonicum* miRNAs. (PDF)

S7 Table Primer pairs used to generate mRNA miRNA target regions for pGLU-CMV luciferase vector constructs. (PDF)

S8 Table miRNA mimics used for cell transfection and worm electroporation. (PDF)

S9 Table Primers used for miRNA suppression studies on *in vivo* cultured schistosomes. (PDF)

S10 Table LNAs used for miRNA *in situ* hybridization and the primers for probe preparation. (PDF)

S11 Table Putative miRNAs identified in the sequencing that are not supported by our additional miRNA criteria. (PDF)

S1 File Supplemental materials and methods. (PDF)

S1 Movie Movie showing a Z-stack series of the ovary treated with a miR-31 inhibitor. (AVI)

S2 Movie Movie showing a Z-stack series of the ovary treated with a scrambled miR-31 inhibitor. (AVI)

S3 Movie Movie showing a Z-stack series of the ovary treated with a bantam miRNA inhibitor. (AVI)

S4 Movie Movie showing a Z-stack series of the ovary treated with a scrambled bantam miRNA inhibitor. (AVI)
