# Correction: MicroRNAs Are Involved in the Regulation of Ovary Development in the Pathogenic Blood Fluke *Schistosoma japonicum*

**DOI:** 10.1371/journal.ppat.1005582

**Published:** 2016-04-19

**Authors:** The PLOS Pathogens Staff

In the “miRNA:mRNA duplex” column of Table 2, the spaces between RNA sequences were removed during typesetting. The publisher apologizes for the error. Table 2 is presented here as Fig 1 for improved readability and to preserve the intended formatting. The original source file for Table 2 is also provided here as S1 File.

**Fig 1:**
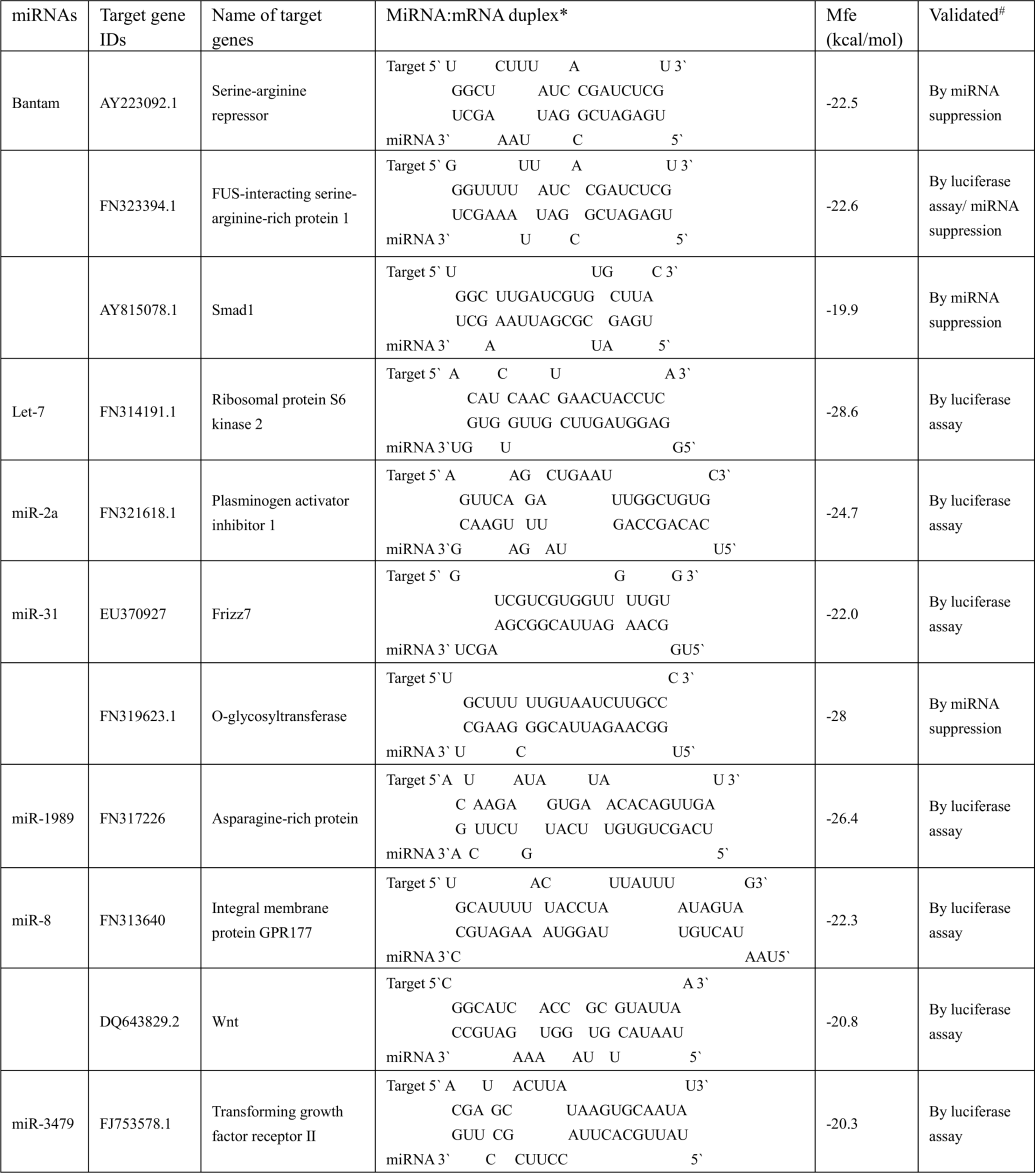
Validated target genes for *S*. *japonicum* miRNAs. *miRNA:mRNA pair analysis was performed using RNAhybrid (http://bibiserv.techfak.uni-bielefeld.de/rnahybrid/) ^#^luciferase assay = miRNA mimics down-regulate target mRNA sequences in mammalian cells; miRNA suppression = transfection of antisense miRNA sequences into schistosomes leads to increases in target mRNAs

## Supporting Information

S1 File Original Table 2. (DOCX)
