# Supplementary figures and images for: MicroRNAs Are Involved in the Regulation of Ovary Development in the Pathogenic Blood Fluke Schistosoma japonicum
Source: PLoS Pathog. 2016 Feb 12;12(2):e1005423. doi: 10.1371/journal.ppat.1005423 (PMC4752461; doi:10.1371/journal.ppat.1005423)

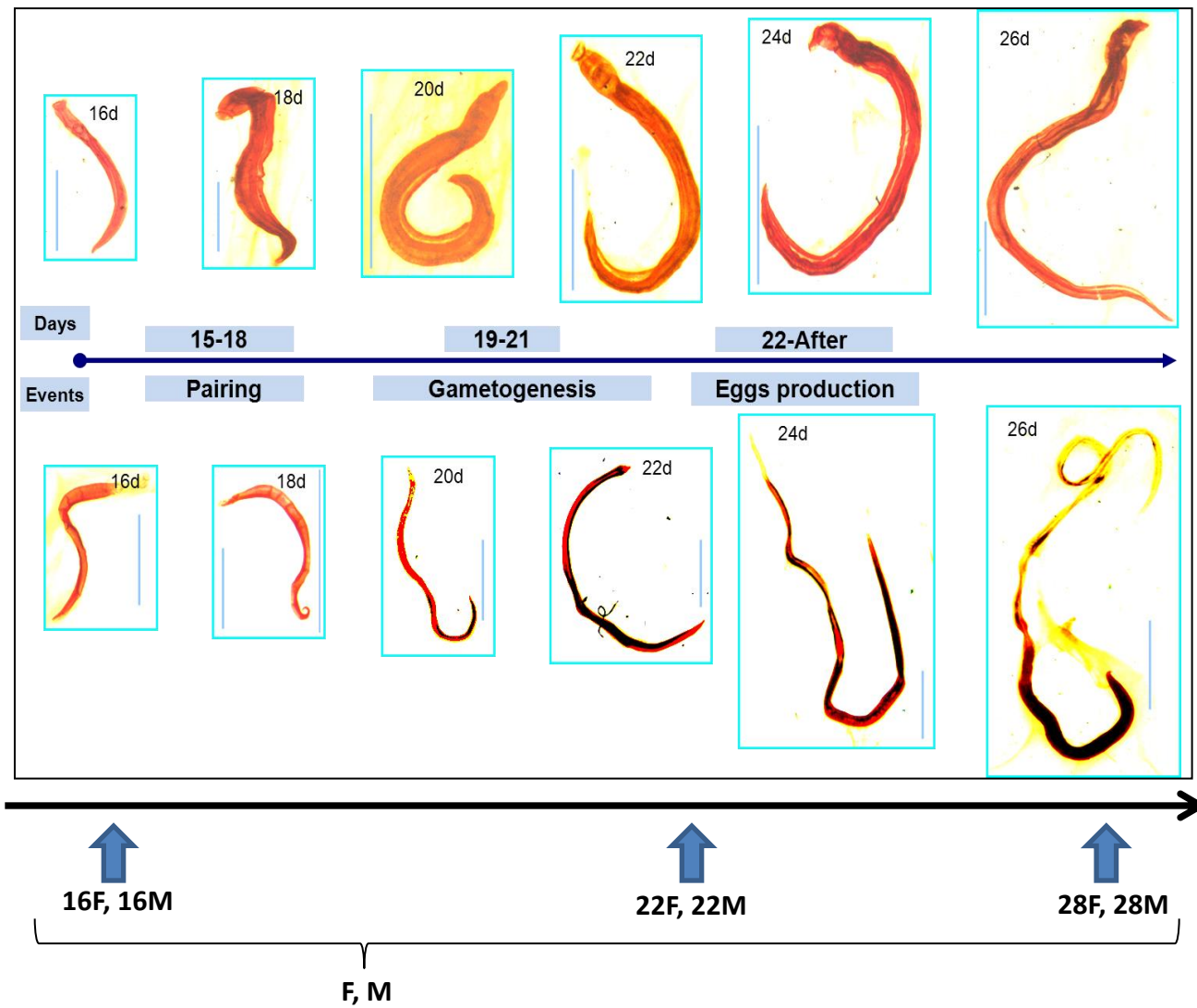

Supplementary Fig. 1

Supplement: S1 Fig — Pairing of S. japonicum males and females usually occurs 15–18 days post-infection in the mammalian host, gametogenesis begins at 19–21 days post-infection, and male and female schistosomes begin to produce mature gametes at 22 days post-infection. (PDF) [file ppat.1005423.s001.pdf]

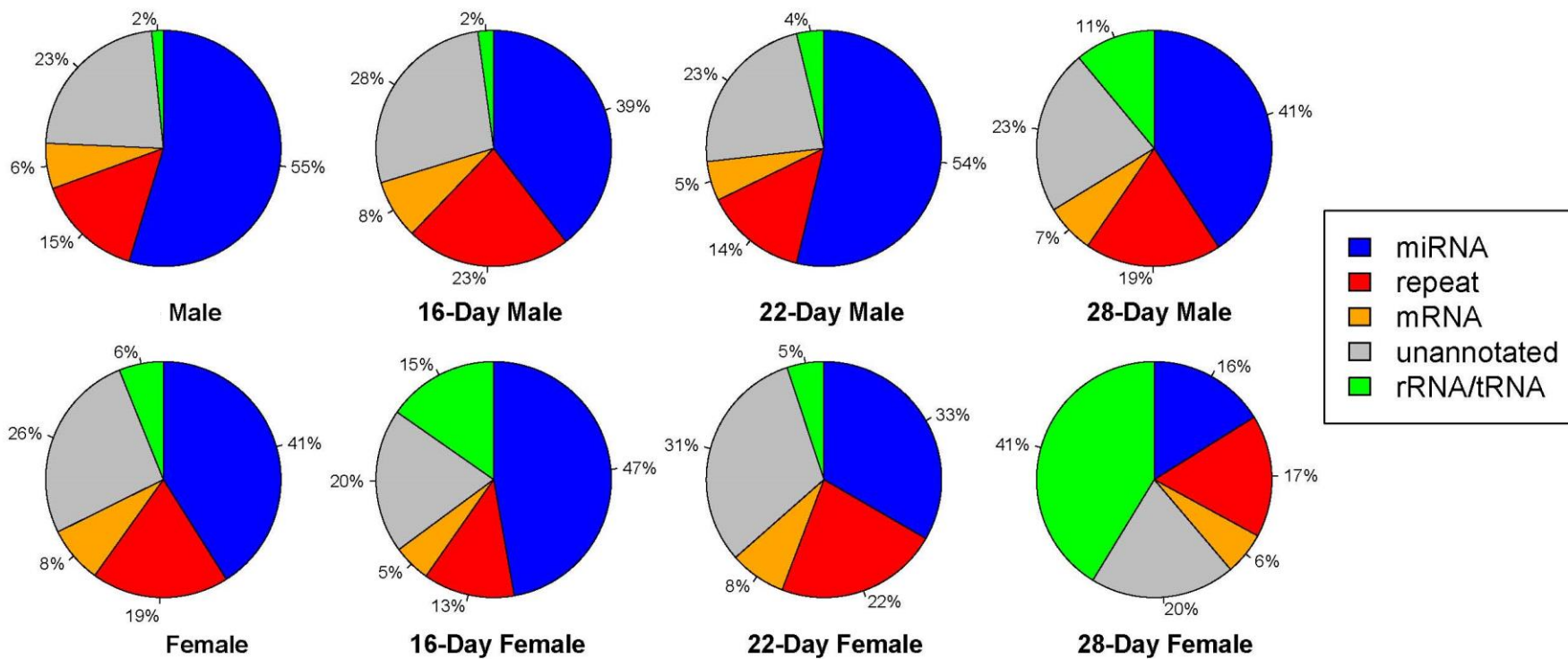

Supplementary Fig. 2A

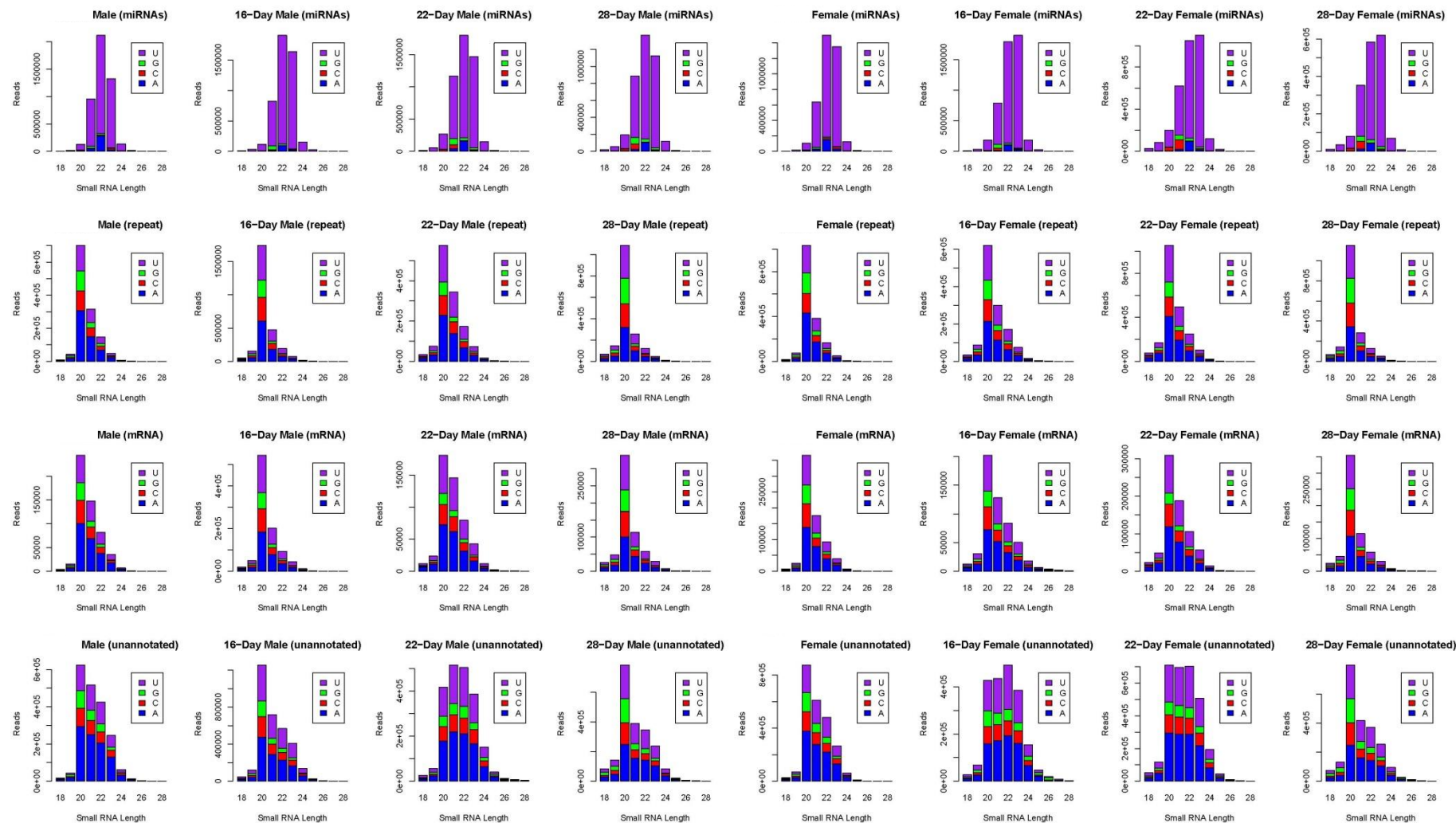

Supplementary Fig. 2B

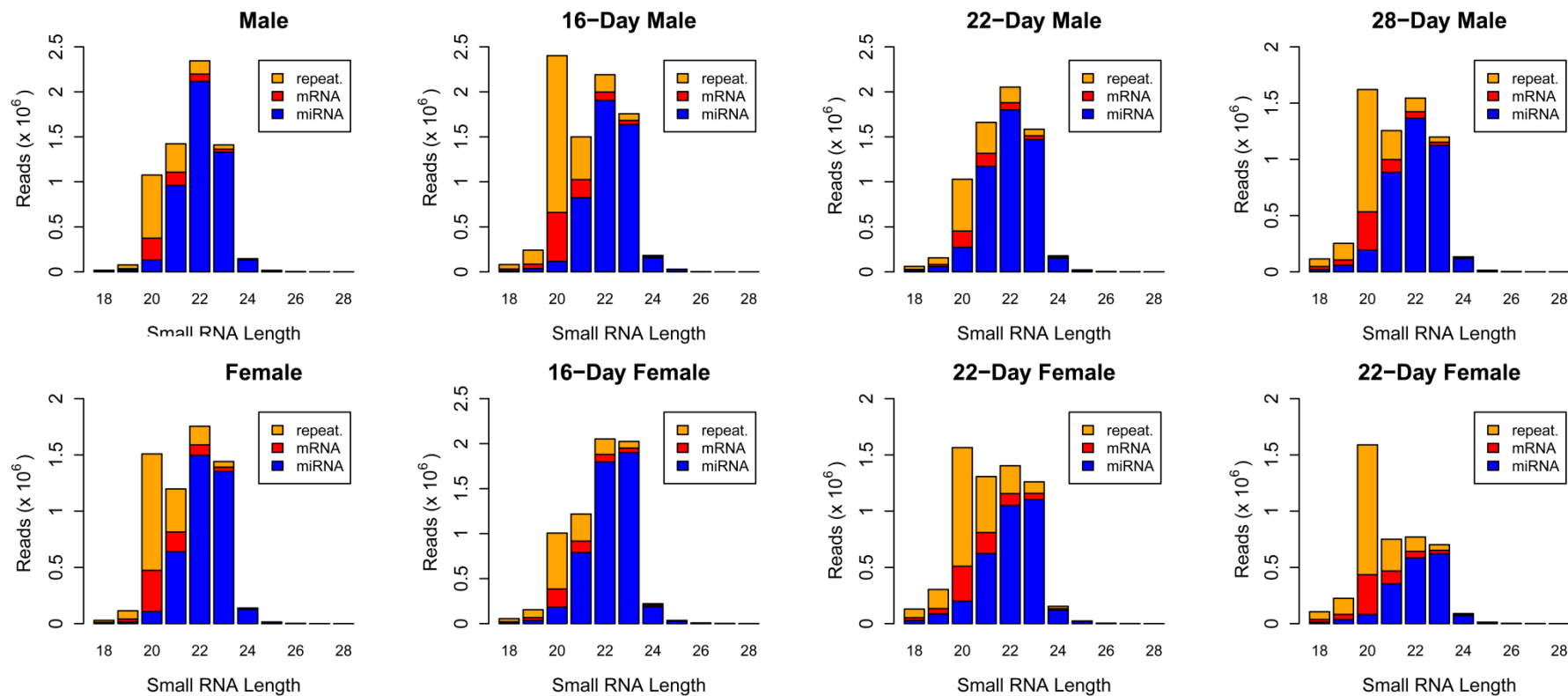

Supplementary Fig. 2C

Supplement: S2 Fig — (A) Classification of small RNAs in 16-day old females (16F), 16-day old males (16M), 22-day old females (22F), 22-day old males (22M), 28-day old females (28F), 28-day old males (28M), and mixed males (M) and mixed females (F). (B) Size distribution of small RNAs in different S. japonicum stages. (C) Classification and percentage of S. japonicum small RNAs from different stages and sexes. Unannotated = small RNAs that map to the genome, but the genome regions are not annotated. (PDF) [file ppat.1005423.s002.pdf]

A

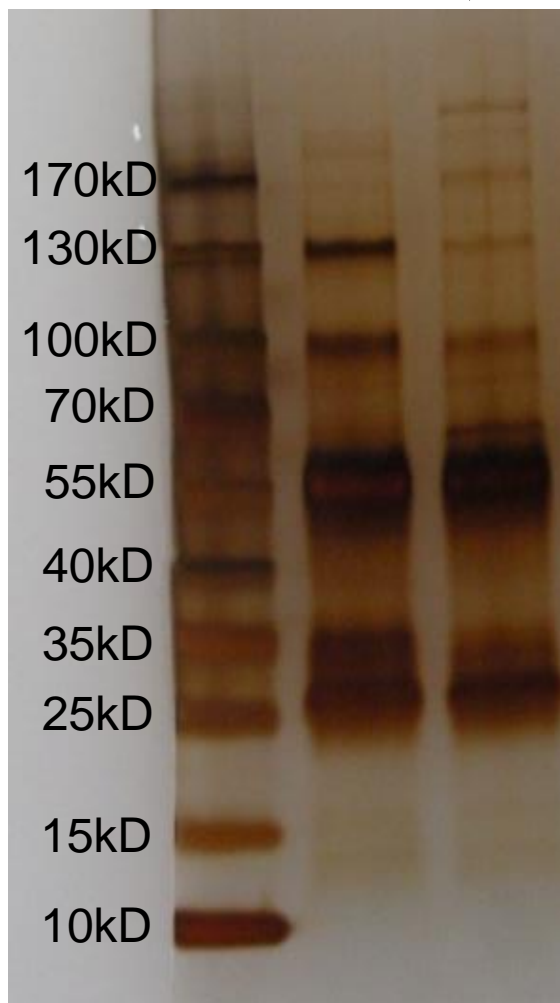

B

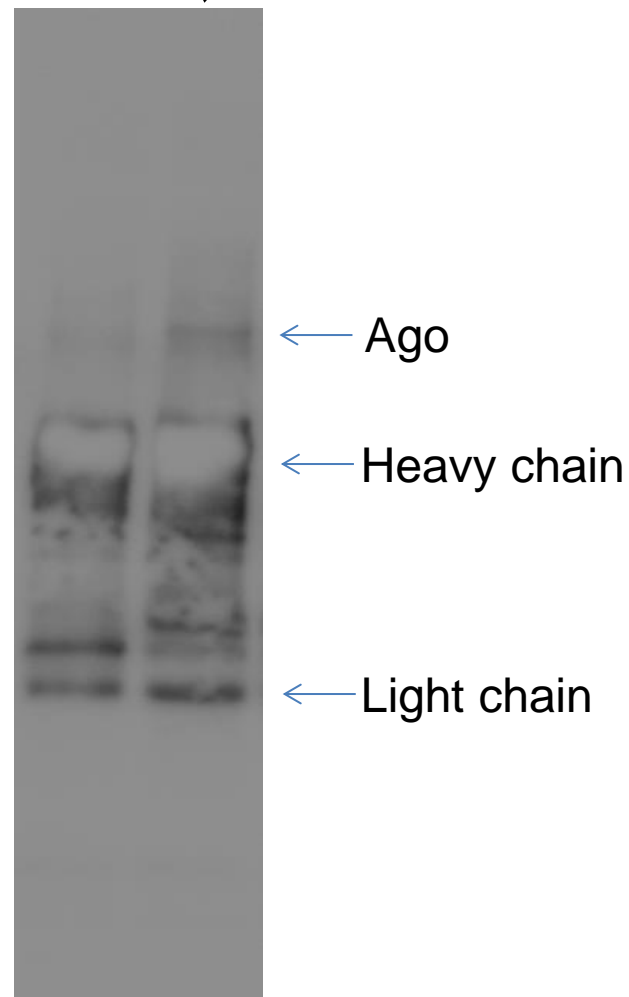

Supplementary Fig. 3

Supplement: S3 Fig — (A) Analysis of pull down products based on SDS-PAGE and silver staining. (B) Western blot analysis of the pull-downs. (PDF) [file ppat.1005423.s003.pdf]

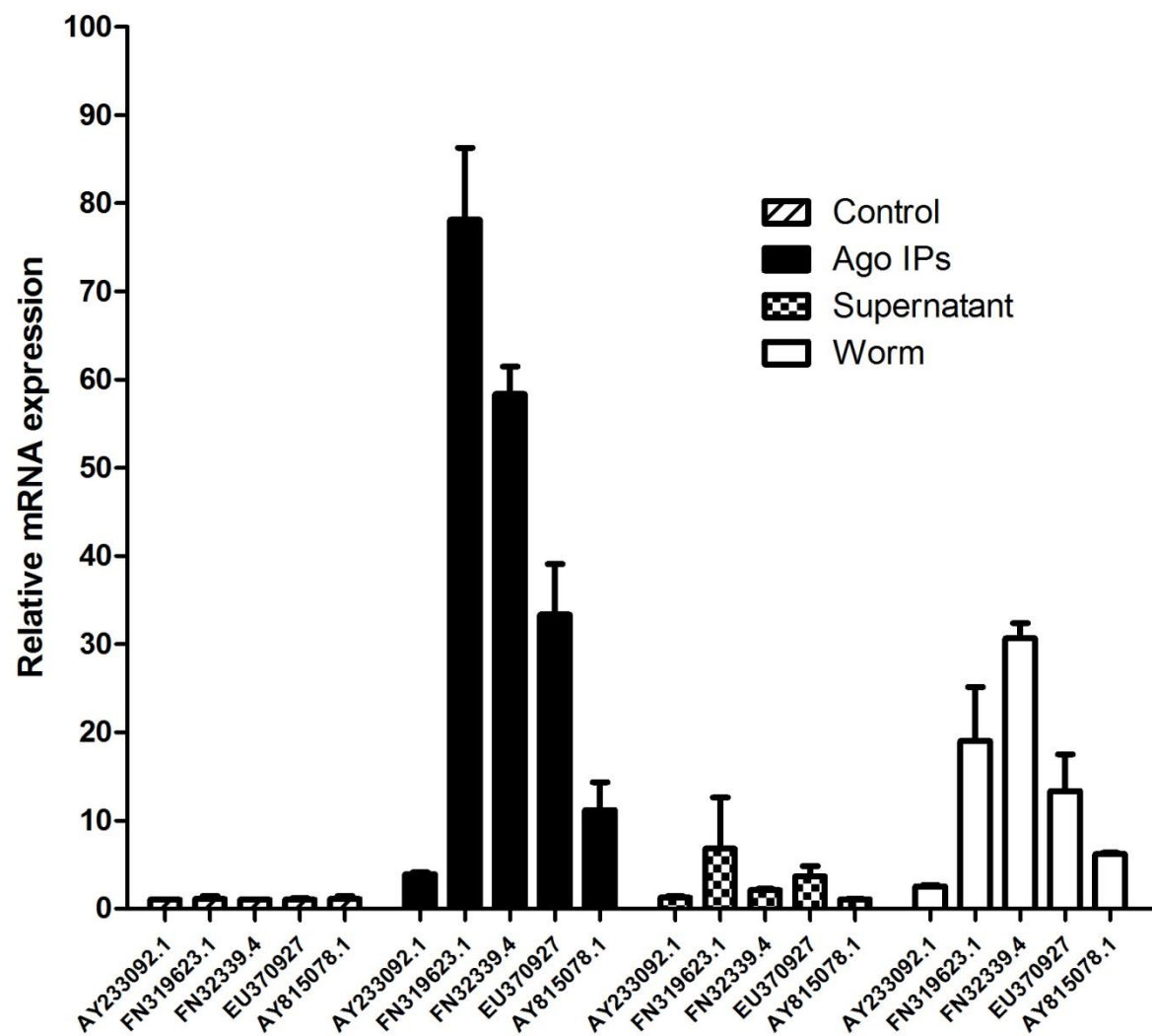

Supplementary Fig. 5

Supplement: S5 Fig — (PDF) [file ppat.1005423.s005.pdf]

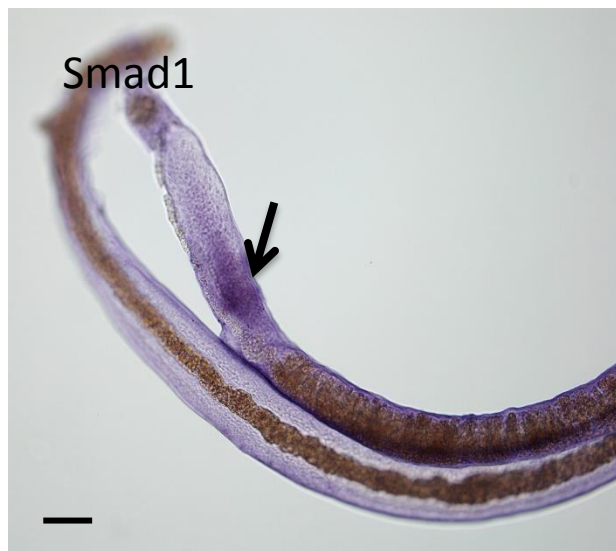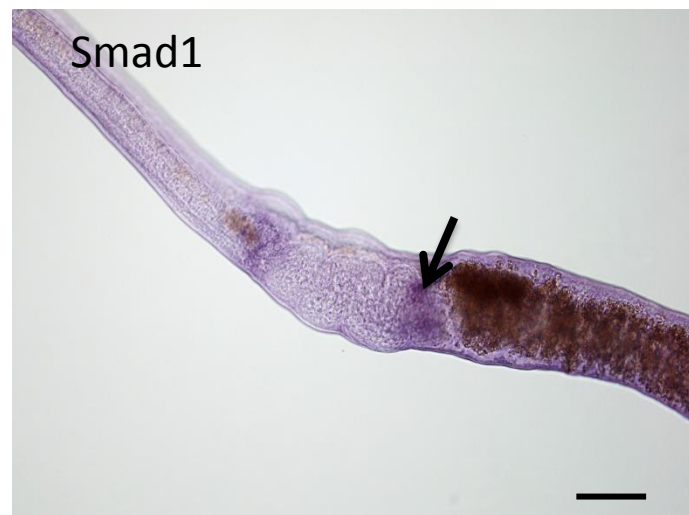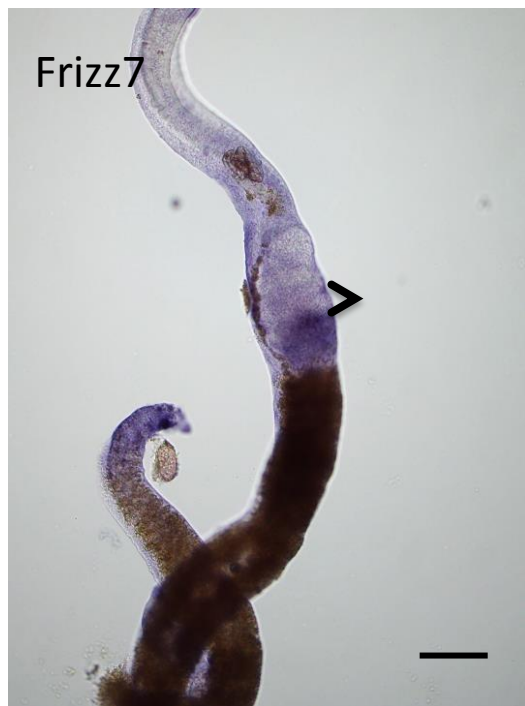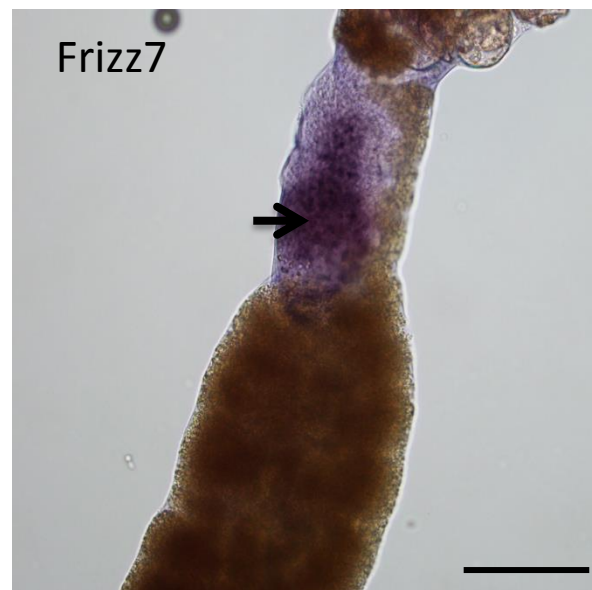

Supplementary Fig. 6

Supplement: S6 Fig — Arrows indicate ovary in S. japonicum. Bars indicate 100 μm. (PDF) [file ppat.1005423.s006.pdf]

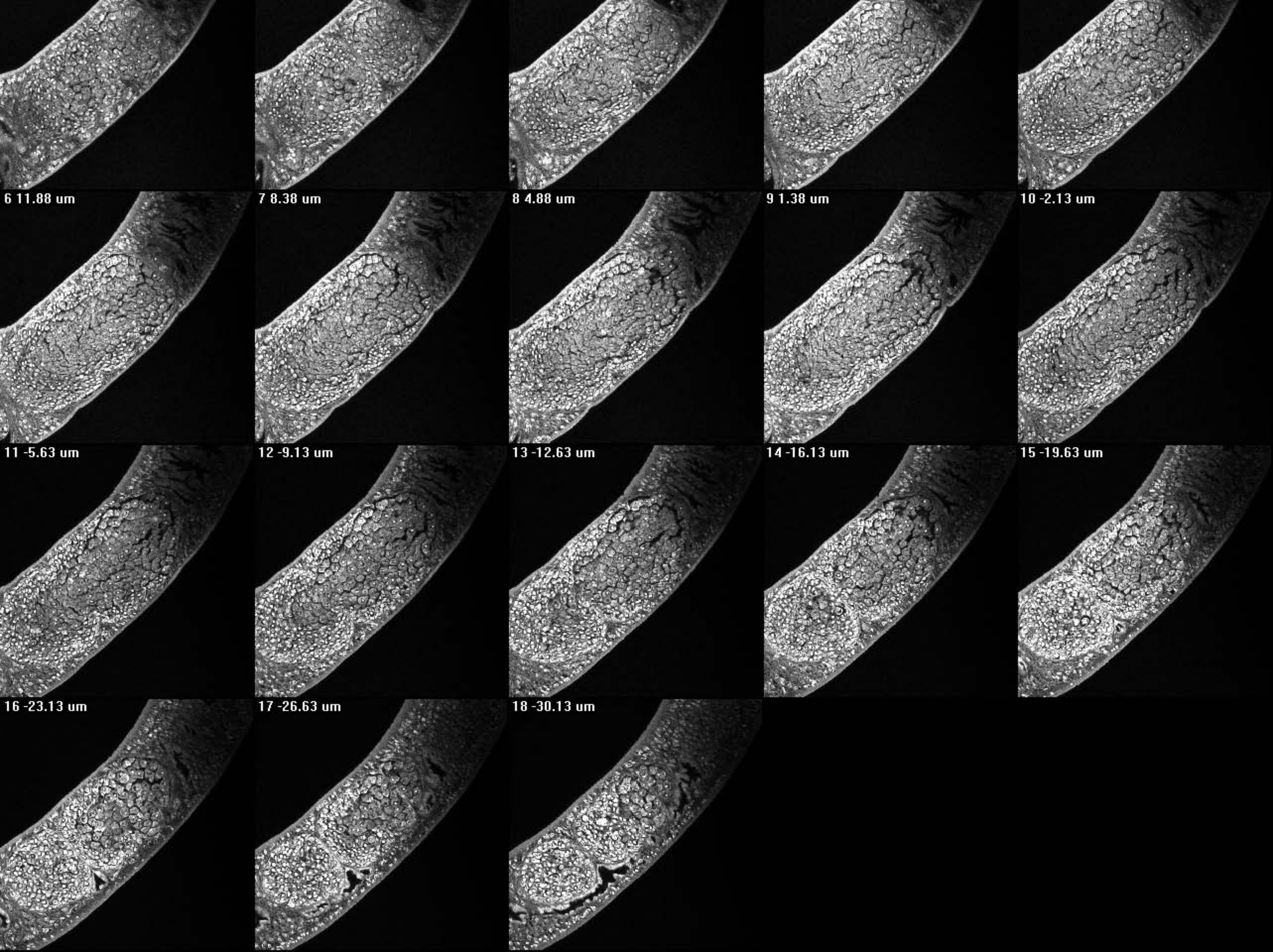

6 11.88 um

7 8.38 um

8 4.88 um

9 1.38 um

10 -2.13 um

11 -5.63 um

12 -9.13 um

13 -12.63 um

14 -16.13 um

15 -19.63 um

16 -23.13 um

17 -26.63 um

18 -30.13 um

Supplement: S8 Fig — (PDF) [file ppat.1005423.s008.pdf]

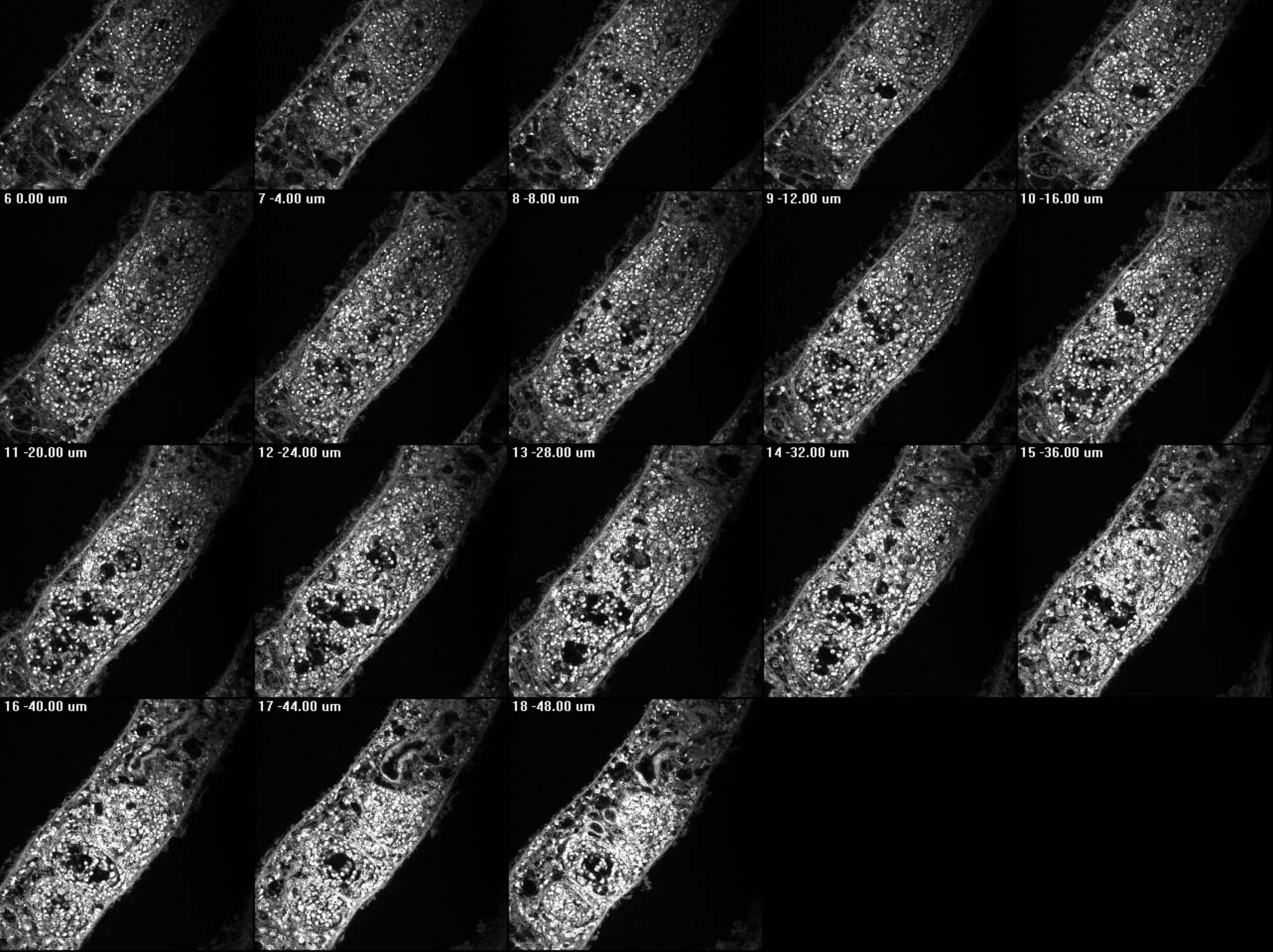

Supplement: S9 Fig — (PDF) [file ppat.1005423.s009.pdf]

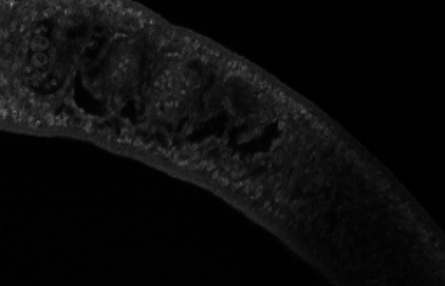

6 -22.05 um

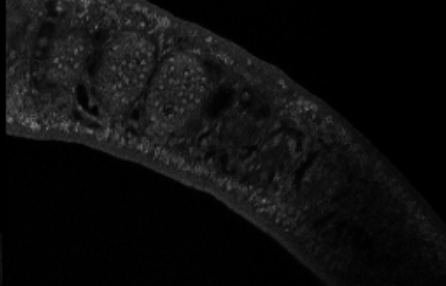

7 -26.05 um

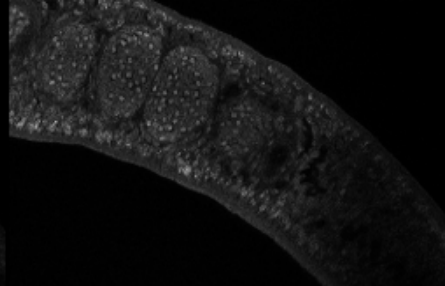

8 -30.05 um

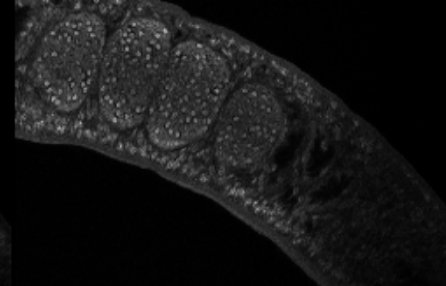

9 -34.05 um

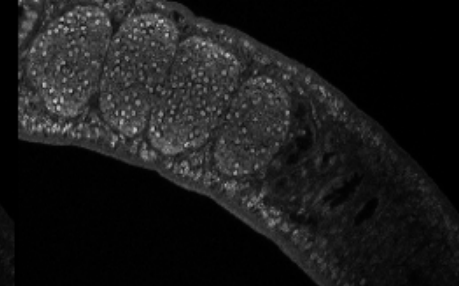

10 -38.05 um

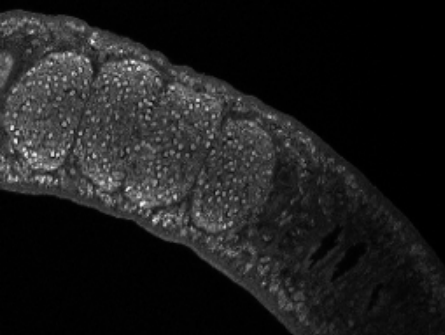

11 -42.05 um

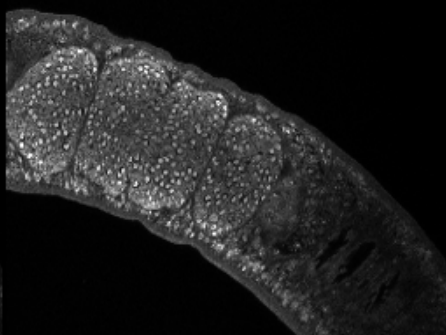

12 -46.05 um

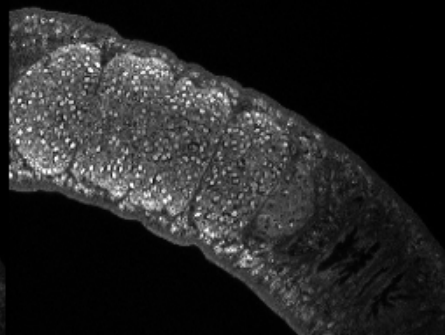

13 -50.05 um

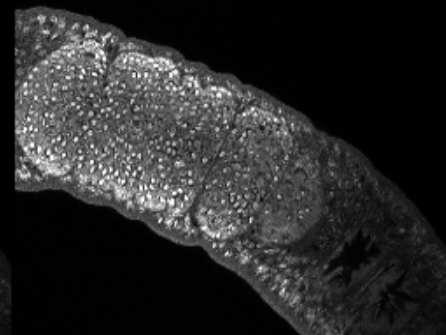

14 -54.05 um

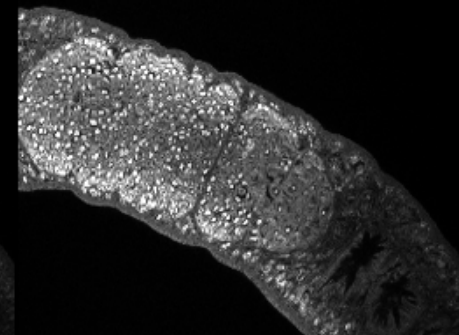

15 -58.05 um

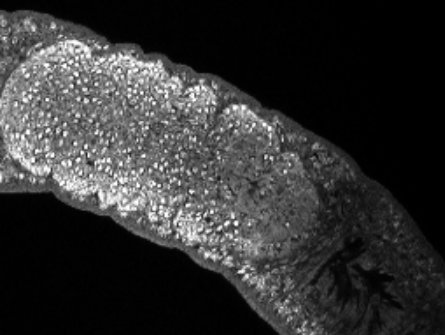

16 -62.05 um

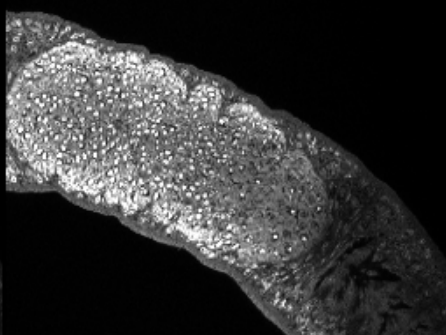

17 -66.05 um

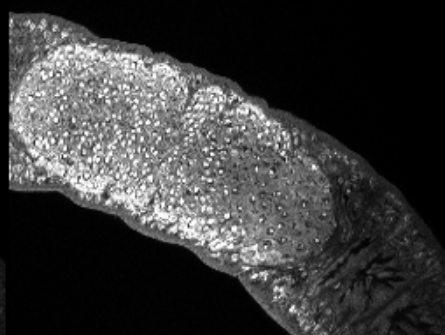

18 -70.05 um

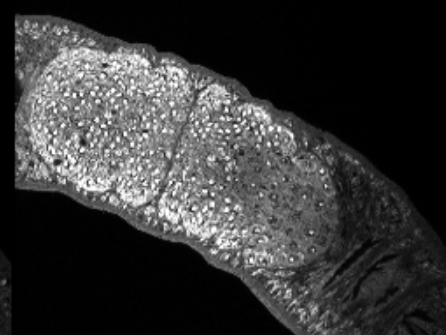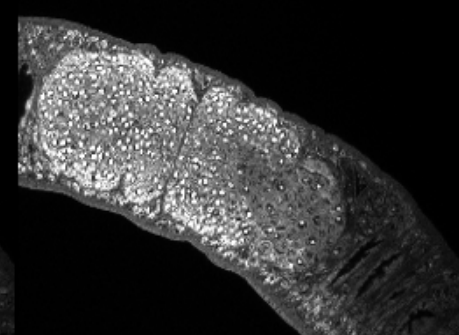

Supplement: S10 Fig — (PDF) [file ppat.1005423.s010.pdf]

A

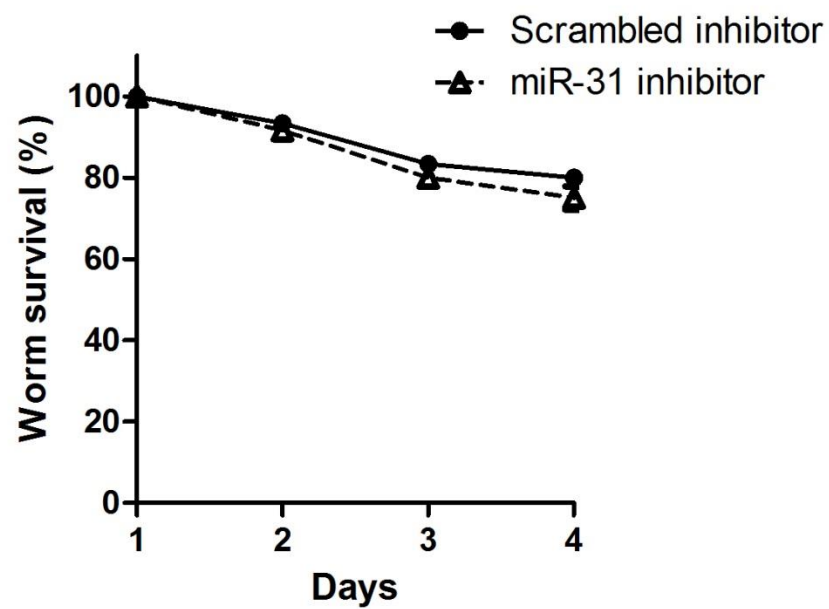

B

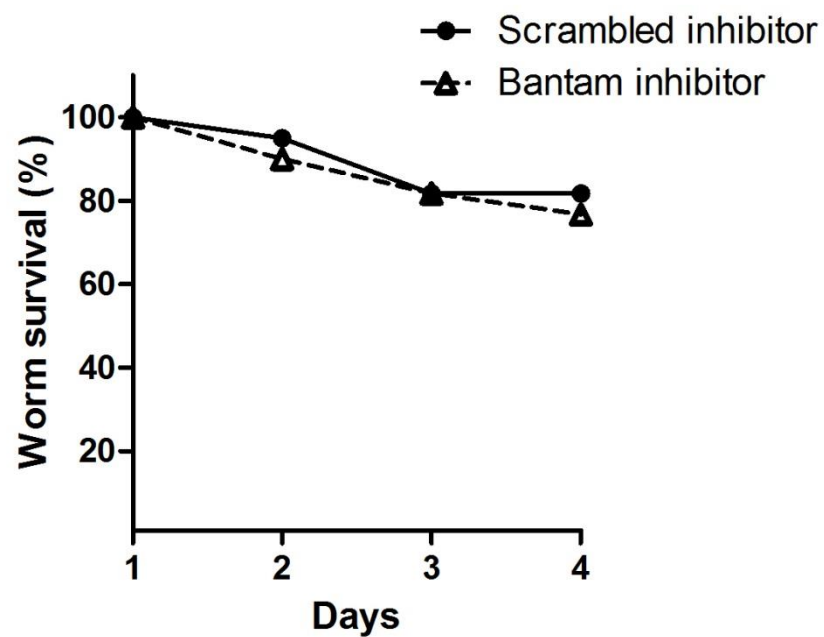

Supplement: S11 Fig — (A) Effect of miR-31 suppression on worm mortality in female schistosomes. (B) Effect of bantam suppression on worm mortality in female schistosomes. Data illustrate the mean and standard error derived from triplicate experiments including at least 30 female schistosomes. (PDF) [file ppat.1005423.s011.pdf]

A

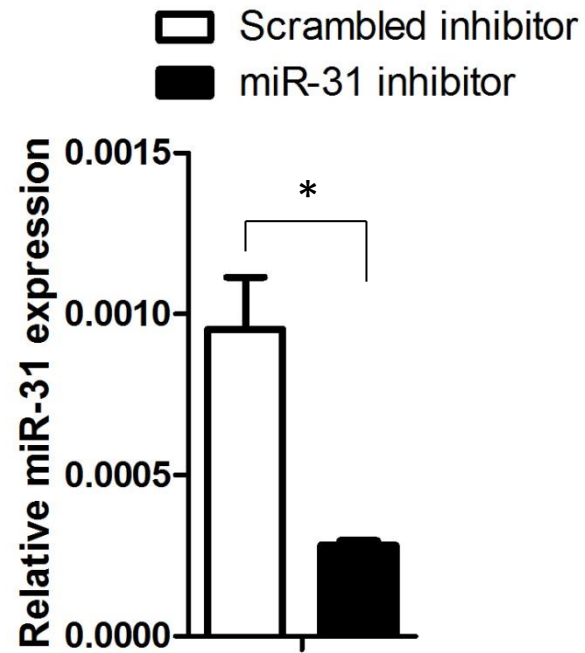

B

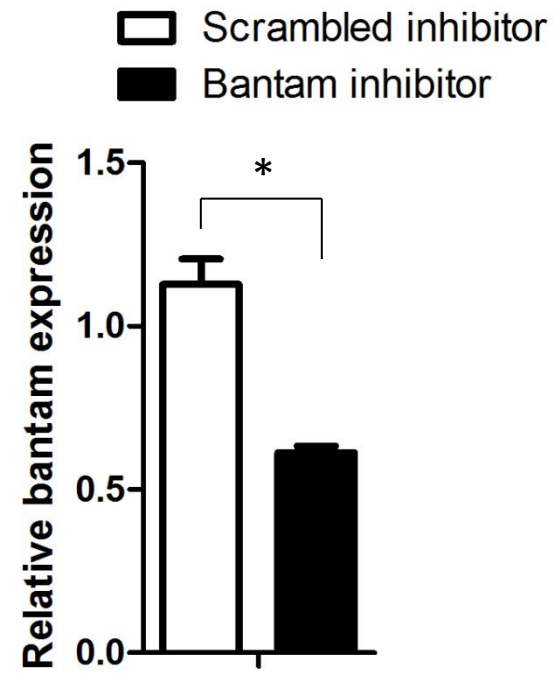

Supplement: S12 Fig — The female schistosomes were electroporated with anti-miRNAs or scrambled anti-miRNAs and their effects on the levels of endogenous miRNA was determined by qRT-PCR at 4 days of post-electroporation. Data illustrate the mean and standard error derived from triplicate experiments. * means P ≤ 0.05 (student’s t test, miRNA inhibitor treatment vs scrambled inhibitor treatment). (PDF) [file ppat.1005423.s012.pdf]
