# Supplementary material for: MicroRNAs Are Involved in the Regulation of Ovary Development in the Pathogenic Blood Fluke Schistosoma japonicum
Source: PLoS Pathog. 2016 Feb 12;12(2):e1005423. doi: 10.1371/journal.ppat.1005423 (PMC4752461; doi:10.1371/journal.ppat.1005423)
Supplement: S7 Fig — (PDF) [file ppat.1005423.s007.pdf]

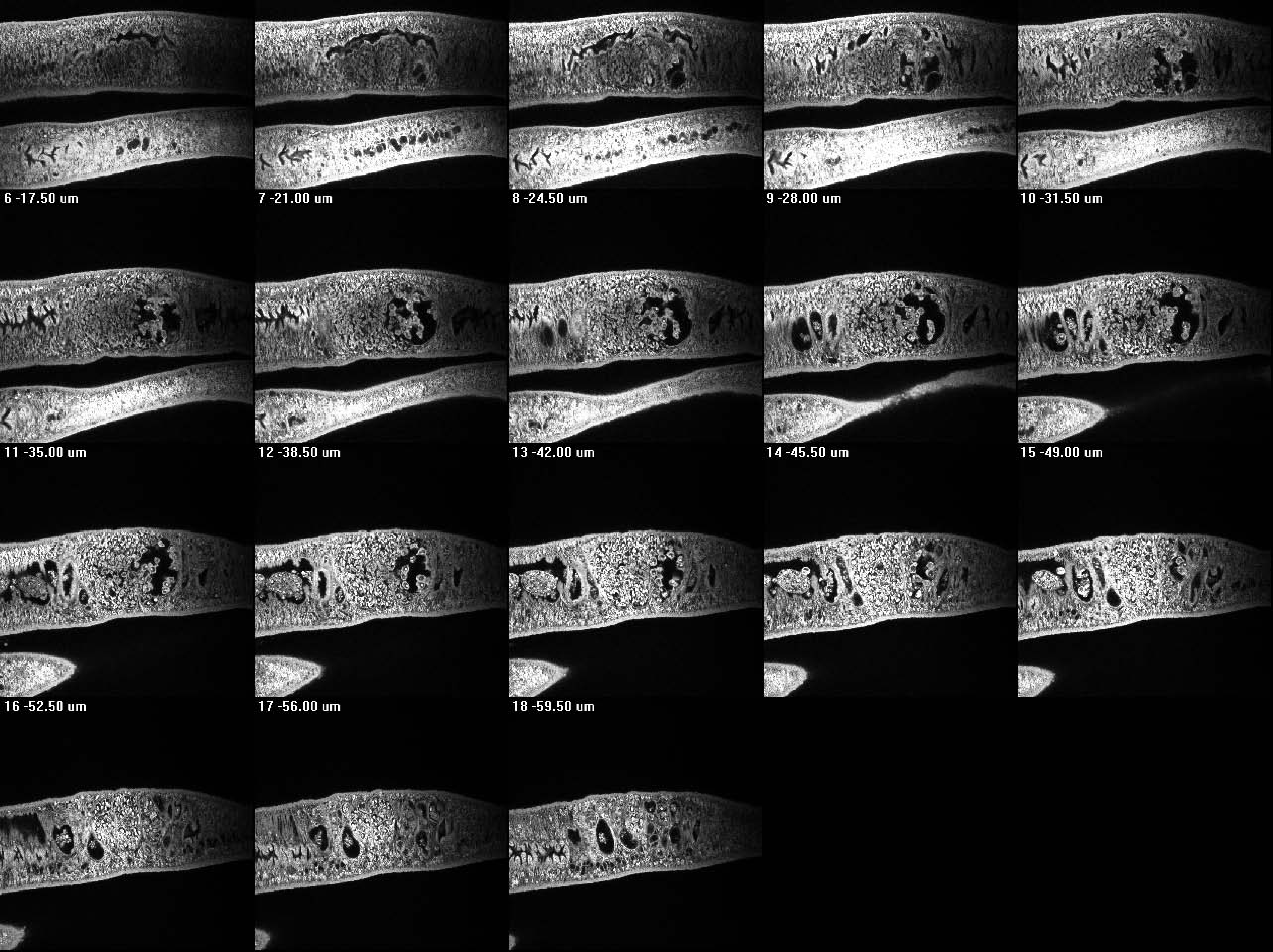

6 -17.50 um
7 -21.00 um
8 -24.50 um
9 -28.00 um
10 -31.50 um
11 -35.00 um
12 -38.50 um
13 -42.00 um
14 -45.50 um
15 -49.00 um
16 -52.50 um
17 -56.00 um
18 -59.50 um
