# Supplementary material for: MicroRNAs Are Involved in the Regulation of Ovary Development in the Pathogenic Blood Fluke Schistosoma japonicum
Source: PLoS Pathog. 2016 Feb 12;12(2):e1005423. doi: 10.1371/journal.ppat.1005423 (PMC4752461; doi:10.1371/journal.ppat.1005423)
Supplement: S13 Fig — The length and read number of the small RNA are indicated at the end of each small RNA sequence. The reads numbers are the sum of small RNA reads in all of the 8 libraries. (PDF) [file ppat.1005423.s013.pdf]

```
#sja-bantam
AAGTCGGCTTTTATTGCGCTCTGAGAAAAACCAATTCATTGCTTTTTTTATTTATCTGAGATCGCGATTAAAGCTGGTTT
Total sRNA: 218038        shared 5' end with miRNA: 129751   shared 3' end with miRNA: 19
miRNA
........................................................UGAGAUCGCGAUUAAAGCU.....      19       88059
Shared 5' Terminus
........................................................UGAGAUCGCGAUUAAAGC......      18       34968
........................................................UGAGAUCGCGAUUAAAGCUG....      20       24052
........................................................UGAGAUCGCGAUUAAAGCUGG...      21       15318
........................................................UGAGAUCGCGAUUAAAGCUGGU..      22       53818
........................................................UGAGAUCGCGAUUAAAGCUGGUU.      23       984
........................................................UGAGAUCGCGAUUAAAGCUGGUUU      24       611
Shared 3' Terminus
.........................................................GAGAUCGCGAUUAAAGCU.....      18       15
Subsequence
Others
....CGGCUUUUAUUGCGCUCUGA........................................................      20       5
....CGGCUUUUAUUGCGCUCUGAGA......................................................      22       46
..........................AAAACCAAUUCAUUGCUUUUU.................................      21       9
..........................AAAACCAAUUCAUUGCUUUUUU................................      22       5
.........................................................GAGAUCGCGAUUAAAGCUG....      19       13
.........................................................GAGAUCGCGAUUAAAGCUGG...      20       8
.........................................................GAGAUCGCGAUUAAAGCUGGU..      21       87
.........................................................GAGAUCGCGAUUAAAGCUGGUU.      22       5
antisense match


#sja-let-7
TGGGAGGTAGTTCGTTGTGTGGTTTGCTTATTAAAATGATCTTAAGAAGACCATACAACCGACTGGCTTTCCCA
Total sRNA: 317022        shared 5' end with miRNA: 39924   shared 3' end with miRNA: 714
miRNA
..GGAGGUAGUUCGUUGUGUGGU...................................................   21       258334
Shared 5' Terminus
..GGAGGUAGUUCGUUGUGU......................................................   18       1101
..GGAGGUAGUUCGUUGUGUG.....................................................   19       1402
..GGAGGUAGUUCGUUGUGUGG....................................................   20       7735
..GGAGGUAGUUCGUUGUGUGGUU..................................................   22       25526
..GGAGGUAGUUCGUUGUGUGGUUU.................................................   23       4154
..GGAGGUAGUUCGUUGUGUGGUUUG................................................   24       5
Shared 3' Terminus
.....GGUAGUUCGUUGUGUGGU...................................................   18       43
....AGGUAGUUCGUUGUGUGGU...................................................   19       84
...GAGGUAGUUCGUUGUGUGGU...................................................   20       580
.GGGAGGUAGUUCGUUGUGUGGU...................................................   22       6
Subsequence
...GAGGUAGUUCGUUGUGUG.....................................................   18       327
...GAGGUAGUUCGUUGUGUGG....................................................   19       72
....AGGUAGUUCGUUGUGUGG....................................................   18       19
Others
...GAGGUAGUUCGUUGUGUGGUU..................................................   21       169
...GAGGUAGUUCGUUGUGUGGUUU.................................................   22       160
....AGGUAGUUCGUUGUGUGGUU..................................................   20       26
....AGGUAGUUCGUUGUGUGGUUU.................................................   21       21
.....GGUAGUUCGUUGUGUGGUU..................................................   19       5
.......UAGUUCGUUGUGUGGUUUGC...............................................   20       7
.......UAGUUCGUUGUGUGGUUUGCU..............................................   21       14
.......UAGUUCGUUGUGUGGUUUGCUU.............................................   22       40
.......................UUGCUUAUUAAAAUGAUCUU...............................   20       7
.......................UUGCUUAUUAAAAUGAUCUUA..............................   21       16
.......................UUGCUUAUUAAAAUGAUCUUAA.............................   22       8
.......................UUGCUUAUUAAAAUGAUCUUAAGAAGAC.......................   28       9
.........................GCUUAUUAAAAUGAUCUUAAGAAGAC.......................   26       6
...........................UUAUUAAAAUGAUCUUAAGAAGA........................   23       5
............................UAUUAAAAUGAUCUUAAGAAGAC.......................   23       5
.............................................GAAGACCAUACAACCGACUGGCU......   23       21
..............................................AAGACCAUACAACCGACUGGCU......   22       7
..............................................AAGACCAUACAACCGACUGGCUUU....   24       5
..............................................AAGACCAUACAACCGACUGGCUUUC...   25       6
..................................................CCAUACAACCGACUGGCUUUC...   21       5
```

```
...................................................CCAUACAACCGACUGGCUUUCCC.   23   11
..................................................CCAUACAACCGACUGGCUUUCCCA   24   39
...................................................CAUACAACCGACUGGCUU.....   18   22
...................................................CAUACAACCGACUGGCUUU....   19   74
...................................................CAUACAACCGACUGGCUUUC...   20   731
...................................................CAUACAACCGACUGGCUUUCC..   21   1397
...................................................CAUACAACCGACUGGCUUUCCC.   22   1857
...................................................CAUACAACCGACUGGCUUUCCCA   23   8932
....................................................AUACAACCGACUGGCUUU....   18   22
....................................................AUACAACCGACUGGCUUUC...   19   192
....................................................AUACAACCGACUGGCUUUCC..   20   258
....................................................AUACAACCGACUGGCUUUCCC.   21   768
....................................................AUACAACCGACUGGCUUUCCCA   22   2566
.....................................................UACAACCGACUGGCUUUCC..   19   8
.....................................................UACAACCGACUGGCUUUCCC.   20   13
.....................................................UACAACCGACUGGCUUUCCCA   21   63
......................................................ACAACCGACUGGCUUUCCCA   20   52
antisense match
```

```
#sja-let-7b
GTGAATAACTGGAAAGAGGTAGTGATTCATATGACTACTGTGTTACTCCTTGTGTGTGTGTATGTATAGAAAATGTAAAAAATATGTTGTAACAACATACTAAACATTGAAAAAATTAATCTTTTTTCAATGAAATTTTCTTTTGTATACACACATATAAGGTGGTTACATTTTAATGGAAGTCATATGTCTCACTAGCTTCATACTGGGATTCAC
Total sRNA: 703318      shared 5' end with miRNA: 46238     shared 3' end with miRNA: 714
miRNA
..............AGAGGUAGUGAUUCAUAUGACU....................................................................................................................................................................................   22   655165
Shared 5' Terminus
..............AGAGGUAGUGAUUCAUAU........................................................................................................................................................................................   18   542
..............AGAGGUAGUGAUUCAUAUG.......................................................................................................................................................................................   19   1256
..............AGAGGUAGUGAUUCAUAUGA......................................................................................................................................................................................   20   5968
..............AGAGGUAGUGAUUCAUAUGAC.....................................................................................................................................................................................   21   38234
..............AGAGGUAGUGAUUCAUAUGACUA...................................................................................................................................................................................   23   235
Shared 3' Terminus
................AGGUAGUGAUUCAUAUGACU....................................................................................................................................................................................   20   235
...............GAGGUAGUGAUUCAUAUGACU....................................................................................................................................................................................   21   233
.............AAGAGGUAGUGAUUCAUAUGACU....................................................................................................................................................................................   23   244
Subsequence
...............GAGGUAGUGAUUCAUAUGAC.....................................................................................................................................................................................   20   39
................AGGUAGUGAUUCAUAUGAC.....................................................................................................................................................................................   19   34
Others
.............AAGAGGUAGUGAUUCAUAU........................................................................................................................................................................................   19   9
.............AAGAGGUAGUGAUUCAUAUG.......................................................................................................................................................................................   20   6
.............AAGAGGUAGUGAUUCAUAUGA......................................................................................................................................................................................   21   62
.............AAGAGGUAGUGAUUCAUAUGAC.....................................................................................................................................................................................   22   446
................AGGUAGUGAUUCAUAUGACUA...................................................................................................................................................................................   21   5
......................................................................................................................................................................................UCAUAUGUCUCACUAGCU................   18   5
......................................................................................................................................................................................UCAUAUGUCUCACUAGCUU...............   19   5
......................................................................................................................................................................................UCAUAUGUCUCACUAGCUUC..............   20   28
......................................................................................................................................................................................UCAUAUGUCUCACUAGCUUCA.............   21   93
......................................................................................................................................................................................UCAUAUGUCUCACUAGCUUCAU............   22   299
......................................................................................................................................................................................UCAUAUGUCUCACUAGCUUCAUA...........   23   130
.......................................................................................................................................................................................CAUAUGUCUCACUAGCUUCAUA...........   22   15
........................................................................................................................................................................................AUAUGUCUCACUAGCUUCAUA...........   21   7
antisense match
```

```
#sja-let-7s
TCAGTATCAGTGAGGTAGTTAGATGTACGACTGTTGTGAATTGAGGTCGTCCAGTCGACTATCTAATGTGTTCAGAATCTCATTCACTGATGTATCAGTTAATGTGATTGTTTTGTAATTGTGAATAATGTGAGTTGATATATGTAGGTGAAGTGAGTTATTCTGACATATTGGTTAGTTGTACTGGTTGACAGCCGTACACCTTACTACCATGCTGGTGATAC
Total sRNA: 322698      shared 5' end with miRNA: 153285    shared 3' end with miRNA: 35777
miRNA
...........GAGGUAGUUAGAUGUACGACU................................................................................................................................................................................................   21   111993
Shared 5' Terminus
...........GAGGUAGUUAGAUGUACG...................................................................................................................................................................................................   18   290
...........GAGGUAGUUAGAUGUACGA..................................................................................................................................................................................................   19   4044
...........GAGGUAGUUAGAUGUACGAC.................................................................................................................................................................................................   20   8671
...........GAGGUAGUUAGAUGUACGACUG...............................................................................................................................................................................................   22   78507
...........GAGGUAGUUAGAUGUACGACUGU..............................................................................................................................................................................................   23   56913
...........GAGGUAGUUAGAUGUACGACUGUU.............................................................................................................................................................................................   24   4844
...........GAGGUAGUUAGAUGUACGACUGUUG............................................................................................................................................................................................   25   12
Shared 3' Terminus
.............GGUAGUUAGAUGUACGACU................................................................................................................................................................................................   19   52
............AGGUAGUUAGAUGUACGACU................................................................................................................................................................................................   20   136
..........UGAGGUAGUUAGAUGUACGACU................................................................................................................................................................................................   22   35586
Subsequence
............AGGUAGUUAGAUGUACGAC.................................................................................................................................................................................................   19   9
.............GGUAGUUAGAUGUACGAC.................................................................................................................................................................................................   18   10
Others
..........UGAGGUAGUUAGAUGUAC....................................................................................................................................................................................................   18   22
..........UGAGGUAGUUAGAUGUACG...................................................................................................................................................................................................   19   86
..........UGAGGUAGUUAGAUGUACGA..................................................................................................................................................................................................   20   2454
..........UGAGGUAGUUAGAUGUACGAC.................................................................................................................................................................................................   21   3677
..........UGAGGUAGUUAGAUGUACGACUG...............................................................................................................................................................................................   23   10045
..........UGAGGUAGUUAGAUGUACGACUGU..............................................................................................................................................................................................   24   930
..........UGAGGUAGUUAGAUGUACGACUGUU.............................................................................................................................................................................................   25   39
............AGGUAGUUAGAUGUACGACUG...............................................................................................................................................................................................   21   195
............AGGUAGUUAGAUGUACGACUGU..............................................................................................................................................................................................   22   273
............AGGUAGUUAGAUGUACGACUGUU.............................................................................................................................................................................................   23   58
.............GGUAGUUAGAUGUACGACUG...............................................................................................................................................................................................   20   314
.............GGUAGUUAGAUGUACGACUGU..............................................................................................................................................................................................   21   141
.............GGUAGUUAGAUGUACGACUGUU.............................................................................................................................................................................................   22   67
```

..............GUAGUUAGAUGUACGACUG... 19 6
..............GUAGUUAGAUGUACGACUGU.. 20 6
....UGACAUAUUGGUUAGUUGUACU....................................... 22 7
....UGACAUAUUGGUUAGUUGUACUG...................................... 23 65
....AGCCGUACACCUUACUACCAU........... 21 9
....AGCCGUACACCUUACUACCAUG.......... 22 17
....GCCGUACACCUUACUACCA............ 19 6
....GCCGUACACCUUACUACCAU........... 20 81
....GCCGUACACCUUACUACCAUG.......... 21 250
....GCCGUACACCUUACUACCAUGC......... 22 2712
....GCCGUACACCUUACUACCAUGCU........ 23 53
....CCGUACACCUUACUACCAUGC......... 21 33
....CCGUACACCUUACUACCAUGCU........ 22 22
....CGUACACCUUACUACCAUGC......... 20 8
....CGUACACCUUACUACCAUGCU........ 21 7
antisense match

#sja-lin-4
NNTCCCTGAGACCTTAGAGTTGTNN
Total sRNA: 24734 shared 5' end with miRNA: 959 shared 3' end with miRNA: 208
miRNA
..UCCCUGAGACCUUAGAGUUGU.. 21 23558
Shared 5' Terminus
..UCCCUGAGACCUUAGAGU..... 18 78
..UCCCUGAGACCUUAGAGUU.... 19 126
..UCCCUGAGACCUUAGAGUUG... 20 755
Shared 3' Terminus
....CCUGAGACCUUAGAGUUGU.. 19 205
Subsequence
....CCUGAGACCUUAGAGUUG... 18 9
Others
antisense match

#sja-mir-1
CAAAAGTAGTATTCATTATATGGAATGTGGCGAAGTATGGTCTACAGTTGCTAGAATCATCTTTATATGAAGAGTATTCACTTTT
Total sRNA: 9075799 shared 5' end with miRNA: 4455011 shared 3' end with miRNA: 20575
miRNA
....................UGGAAUGUGGCGAAGUAUGGUC... 22 4570978
Shared 5' Terminus
....................UGGAAUGUGGCGAAGUAU... 18 36283
....................UGGAAUGUGGCGAAGUAUG.. 19 65142
....................UGGAAUGUGGCGAAGUAUGG... 20 486032
....................UGGAAUGUGGCGAAGUAUGGU.. 21 2957888
....................UGGAAUGUGGCGAAGUAUGGUCU.. 23 905778
....................UGGAAUGUGGCGAAGUAUGGUCUA... 24 3877
....................UGGAAUGUGGCGAAGUAUGGUCUAC.. 25 11
Shared 3' Terminus
.......................AAUGUGGCGAAGUAUGGUC... 19 8
......................GAAUGUGGCGAAGUAUGGUC... 20 640
.....................GGAAUGUGGCGAAGUAUGGUC... 21 18090
...................AUGGAAUGUGGCGAAGUAUGGUC... 23 1766
..................UAUGGAAUGUGGCGAAGUAUGGUC... 24 50
................UAUAUGGAAUGUGGCGAAGUAUGGUC... 26 6
...............UUAUAUGGAAUGUGGCGAAGUAUGGUC... 27 13
Subsequence
.....................GGAAUGUGGCGAAGUAUG.. 18 97
.....................GGAAUGUGGCGAAGUAUGG... 19 1730
.....................GGAAUGUGGCGAAGUAUGGU.. 20 11082
......................GAAUGUGGCGAAGUAUGG... 18 41
......................GAAUGUGGCGAAGUAUGGU.. 19 307
Others
................UAUAUGGAAUGUGGCGAAGUAUGGU.. 25 7
..................UAUGGAAUGUGGCGAAGUA.. 19 15
..................UAUGGAAUGUGGCGAAGUAU... 20 37
..................UAUGGAAUGUGGCGAAGUAUG.. 21 46
..................UAUGGAAUGUGGCGAAGUAUGG... 22 618
..................UAUGGAAUGUGGCGAAGUAUGGU.. 23 1978
..................UAUGGAAUGUGGCGAAGUAUGGUCU.. 25 19
...................AUGGAAUGUGGCGAAGUA.. 18 36
...................AUGGAAUGUGGCGAAGUAU... 19 49
...................AUGGAAUGUGGCGAAGUAUG.. 20 106
...................AUGGAAUGUGGCGAAGUAUGG... 21 1077

```
...................AUGGAAUGUGGCGAAGUAUGGU............................................ 22 8293
...................AUGGAAUGUGGCGAAGUAUGGUCU.......................................... 24 307
.....................GGAAUGUGGCGAAGUAUGGUCU.......................................... 22 3222
.....................GGAAUGUGGCGAAGUAUGGUCUA......................................... 23 28
......................GAAUGUGGCGAAGUAUGGUCU.......................................... 21 111
.........................UGUGGCGAAGUAUGGUCU.......................................... 18 5
antisense match


#sja-mir-10
CCTCAGTATGAACCCTGTAGACCCGAGTTTGGATGCCGTTAGATGCAAATTCGAGTCTATAAGGAAAGATACTTTGGAA
Total sRNA: 90192shared 5' end with miRNA: 11435   shared 3' end with miRNA: 258
miRNA
..........AACCCUGUAGACCCGAGUUUGG...............................................  22  77153
Shared 5' Terminus
..........AACCCUGUAGACCCGAGU...................................................  18  282
..........AACCCUGUAGACCCGAGUU..................................................  19  109
..........AACCCUGUAGACCCGAGUUU.................................................  20  799
..........AACCCUGUAGACCCGAGUUUG................................................  21  4176
..........AACCCUGUAGACCCGAGUUUGGA..............................................  23  6022
..........AACCCUGUAGACCCGAGUUUGGAU.............................................  24  44
Shared 3' Terminus
.............CCUGUAGACCCGAGUUUGG...............................................  19  7
...........ACCCUGUAGACCCGAGUUUGG...............................................  21  188
.........GAACCCUGUAGACCCGAGUUUGG...............................................  23  62
Subsequence
...........ACCCUGUAGACCCGAGUUUG................................................  20  8
.............CCUGUAGACCCGAGUUUG................................................  18  5
Others
.........GAACCCUGUAGACCCGAGUUU.................................................  21  5
.........GAACCCUGUAGACCCGAGUUUG................................................  22  13
.........GAACCCUGUAGACCCGAGUUUGGA..............................................  24  9
...........ACCCUGUAGACCCGAGUUUGGA..............................................  22  20
..............................................AAAUUCGAGUCUAUAAGG...............  18  12
..............................................AAAUUCGAGUCUAUAAGGA..............  19  100
..............................................AAAUUCGAGUCUAUAAGGAA.............  20  184
..............................................AAAUUCGAGUCUAUAAGGAAA............  21  219
..............................................AAAUUCGAGUCUAUAAGGAAAG...........  22  152
..............................................AAAUUCGAGUCUAUAAGGAAAGA..........  23  499
...............................................AAUUCGAGUCUAUAAGGAA.............  19  13
...............................................AAUUCGAGUCUAUAAGGAAA............  20  11
...............................................AAUUCGAGUCUAUAAGGAAAG...........  21  7
...............................................AAUUCGAGUCUAUAAGGAAAGA..........  22  38
.................................................UUCGAGUCUAUAAGGAAAGA..........  20  19
antisense match


#sja-mir-1175
GATATGTACGGTTGGAGTTTTTGTTTCTGCACCAGCCACATATCAGTCTGATTGATACTCCTCACTGTAGGAGATTTTGTCAAATGAACTCTGGTGAGATTCAATTACTTCAACTGTTATATC
Total sRNA: 39868shared 5' end with miRNA: 18988   shared 3' end with miRNA: 59
miRNA
..............................................................................................UGAGAUUCAAUUACUUCAACUG.......  22  20657
Shared 5' Terminus
..............................................................................................UGAGAUUCAAUUACUUCA...........  18  26
..............................................................................................UGAGAUUCAAUUACUUCAA..........  19  154
..............................................................................................UGAGAUUCAAUUACUUCAAC.........  20  576
..............................................................................................UGAGAUUCAAUUACUUCAACU........  21  18150
..............................................................................................UGAGAUUCAAUUACUUCAACUGU......  23  80
Shared 3' Terminus
...............................................................................................GAGAUUCAAUUACUUCAACUG.......  21  50
.............................................................................................GUGAGAUUCAAUUACUUCAACUG.......  23  7
Subsequence
...............................................................................................GAGAUUCAAUUACUUCAACU........  20  46
................................................................................................AGAUUCAAUUACUUCAACU........  19  5
Others
..........GUUGGAGUUUUUGUUUCUGC.............................................................................................  20  6
..........GUUGGAGUUUUUGUUUCUGCACC..........................................................................................  23  22
............UGGAGUUUUUGUUUCUGCACC..........................................................................................  21  5
............UGGAGUUUUUGUUUCUGCACCAG........................................................................................  23  17
```

```
.............................................................................................GUGAGAUUCAAUUACUUCAACU........   22   18
antisense match


#sja-mir-124
TATATGTATGCCATTTTCCGCGATTGCCTTGATTTGTTAAAAGAAAATGATTCACAACAAAATATTAAGGCACGCGGTGAATGTCATCCACGGTGC
Total sRNA: 393245        shared 5' end with miRNA: 47347    shared 3' end with miRNA: 1360
miRNA
.................................................................UAAGGCACGCGGUGAAUGUCA..........   21   343967
Shared 5' Terminus
.................................................................UAAGGCACGCGGUGAAUG.............   18   3534
.................................................................UAAGGCACGCGGUGAAUGU............   19   14185
.................................................................UAAGGCACGCGGUGAAUGUC...........   20   21251
.................................................................UAAGGCACGCGGUGAAUGUCAU.........   22   8348
.................................................................UAAGGCACGCGGUGAAUGUCAUC........   23   27
Shared 3' Terminus
...................................................................AGGCACGCGGUGAAUGUCA..........   19   97
..................................................................AAGGCACGCGGUGAAUGUCA..........   20   1258
Subsequence
..................................................................AAGGCACGCGGUGAAUGU............   18   38
..................................................................AAGGCACGCGGUGAAUGUC...........   19   85
Others
..........CCAUUUUCCGCGAUUGCCUUGA................................................................   22   18
..........CCAUUUUCCGCGAUUGCCUUGAU...............................................................   23   55
..........CCAUUUUCCGCGAUUGCCUUGAUU..............................................................   24   9
..........CCAUUUUCCGCGAUUGCCUUGAUUU.............................................................   25   15
..................................................................AAGGCACGCGGUGAAUGUCAU.........   21   307
...................................................................AGGCACGCGGUGAAUGUCAU.........   20   18
antisense match


#sja-mir-125a
GCTCCCTGAGACCCTTTGATTGTCTCGTTAAACATAATTCTATATGTTAAATGTAATCAGGCAGCCAAAGTGACTCAGGTGTGC
Total sRNA: 19210shared 5' end with miRNA: 9825    shared 3' end with miRNA: 32
miRNA
..UCCCUGAGACCCUUUGAUUGUC............................................................   22   8825
Shared 5' Terminus
..UCCCUGAGACCCUUUGAU................................................................   18   11
..UCCCUGAGACCCUUUGAUU...............................................................   19   30
..UCCCUGAGACCCUUUGAUUG..............................................................   20   1689
..UCCCUGAGACCCUUUGAUUGU.............................................................   21   8081
..UCCCUGAGACCCUUUGAUUGUCU...........................................................   23   14
Shared 3' Terminus
......UGAGACCCUUUGAUUGUC............................................................   18   13
....CCUGAGACCCUUUGAUUGUC............................................................   20   16
Subsequence
....CCUGAGACCCUUUGAUUGU.............................................................   19   6
Others
.............................................................CAGCCAAAGUGACUCAGGUGU..   21   8
.............................................................CAGCCAAAGUGACUCAGGUGUG.   22   60
.............................................................CAGCCAAAGUGACUCAGGUGUGC   23   389
..............................................................AGCCAAAGUGACUCAGGUGUG.   21   5
..............................................................AGCCAAAGUGACUCAGGUGUGC   22   35
...............................................................GCCAAAGUGACUCAGGUGUGC   21   7
antisense match


#sja-mir-125b
ATCCCTGAGACTGATAATTGCTCTAGTTATTATATCATTAATGAGTTTACAATAAGGGCAATTATTATTCTCAGGTGT
Total sRNA: 1007585       shared 5' end with miRNA: 185419   shared 3' end with miRNA: 3526
miRNA
.UCCCUGAGACUGAUAAUUGCU........................................................   21   816687
Shared 5' Terminus
.UCCCUGAGACUGAUAAUU...........................................................   18   3281
.UCCCUGAGACUGAUAAUUG..........................................................   19   3447
.UCCCUGAGACUGAUAAUUGC.........................................................   20   49025
.UCCCUGAGACUGAUAAUUGCUC.......................................................   22   127999
.UCCCUGAGACUGAUAAUUGCUCU......................................................   23   1659
.UCCCUGAGACUGAUAAUUGCUCUA.....................................................   24   8
```

```
Shared 3' Terminus
....CUGAGACUGAUAAUUGCU........................................................        18      249
...CCUGAGACUGAUAAUUGCU........................................................        19      2293
..CCCUGAGACUGAUAAUUGCU........................................................        20      201
AUCCCUGAGACUGAUAAUUGCU........................................................        22      783
Subsequence
..CCCUGAGACUGAUAAUUGC.........................................................        19      11
...CCUGAGACUGAUAAUUGC.........................................................        18      103
Others
AUCCCUGAGACUGAUAAUU...........................................................        19      5
AUCCCUGAGACUGAUAAUUG..........................................................        20      5
AUCCCUGAGACUGAUAAUUGC.........................................................        21      61
AUCCCUGAGACUGAUAAUUGCUC.......................................................        23      109
AUCCCUGAGACUGAUAAUUGCUCU......................................................        24      8
..CCCUGAGACUGAUAAUUGCUC.......................................................        21      113
..CCCUGAGACUGAUAAUUGCUCU......................................................        22      15
...CCUGAGACUGAUAAUUGCUC.......................................................        20      948
...CCUGAGACUGAUAAUUGCUCU......................................................        21      13
....CUGAGACUGAUAAUUGCUC.......................................................        19      97
.....UGAGACUGAUAAUUGCUC.......................................................        18      125
.........................................................GCAAUUAUUAUUCUCAGG...        18      9
.........................................................GCAAUUAUUAUUCUCAGGU..        19      14
.........................................................GCAAUUAUUAUUCUCAGGUG.        20      24
.........................................................GCAAUUAUUAUUCUCAGGUGU        21      264
..........................................................CAAUUAUUAUUCUCAGGUGU        20      8
...........................................................AAUUAUUAUUCUCAGGUGU        19      8
antisense match


#sja-mir-190
CGACAGTAAACTGGTCGATGTGATATGTATGGGTTACTTGGTGTCAGCGGATAACATGTTTTATTCGCCATTAAGCAATACACCAGTGACCAGACATATCCCTATCGCTTAGTTTAGATCCTC
Total sRNA: 296653        shared 5' end with miRNA: 99577   shared 3' end with miRNA: 508
miRNA
....................UGAUAUGUAUGGGUUACUUGGU.................................................................................     22      183425
Shared 5' Terminus
....................UGAUAUGUAUGGGUUACU.....................................................................................     18      579
....................UGAUAUGUAUGGGUUACUU....................................................................................     19      1702
....................UGAUAUGUAUGGGUUACUUG...................................................................................     20      3007
....................UGAUAUGUAUGGGUUACUUGG..................................................................................     21      22892
....................UGAUAUGUAUGGGUUACUUGGUG................................................................................     23      65437
....................UGAUAUGUAUGGGUUACUUGGUGU...............................................................................     24      5881
....................UGAUAUGUAUGGGUUACUUGGUGUC..............................................................................     25      59
....................UGAUAUGUAUGGGUUACUUGGUGUCA.............................................................................     26      20
Shared 3' Terminus
........................AUGUAUGGGUUACUUGGU.................................................................................     18      11
.......................UAUGUAUGGGUUACUUGGU.................................................................................     19      5
......................AUAUGUAUGGGUUACUUGGU.................................................................................     20      13
.....................GAUAUGUAUGGGUUACUUGGU.................................................................................     21      466
...................GUGAUAUGUAUGGGUUACUUGGU.................................................................................     23      6
..................UGUGAUAUGUAUGGGUUACUUGGU.................................................................................     24      6
Subsequence
.....................GAUAUGUAUGGGUUACUUG...................................................................................     19      5
.....................GAUAUGUAUGGGUUACUUGG..................................................................................     20      56
......................AUAUGUAUGGGUUACUUGG..................................................................................     19      6
Others
.GACAGUAAACUGGUCGAUG.......................................................................................................     19      11
..ACAGUAAACUGGUCGAUG.......................................................................................................     18      5
.................AUGUGAUAUGUAUGGGUUACUUG...................................................................................     23      6
.................AUGUGAUAUGUAUGGGUUACUUGG..................................................................................     24      13
..................UGUGAUAUGUAUGGGUUACUUGG..................................................................................     23      14
.....................GAUAUGUAUGGGUUACUUGGUG................................................................................     22      115
.....................GAUAUGUAUGGGUUACUUGGUGU...............................................................................     23      6
......................AUAUGUAUGGGUUACUUGGUG................................................................................     21      18
..........................................GUCAGCGGAUAACAUGUUU..............................................................     19      6
..........................................GUCAGCGGAUAACAUGUUUU.............................................................     20      12
..........................................GUCAGCGGAUAACAUGUUUUA............................................................     21      23
..........................................GUCAGCGGAUAACAUGUUUUAUU..........................................................     23      6
...........................................UCAGCGGAUAACAUGUUU..............................................................     18      5
...........................................UCAGCGGAUAACAUGUUUU.............................................................     19      13
```

```
...........................................UCAGCGGAUAACAUGUUUUA............................................................      20      9
...........................................UCAGCGGAUAACAUGUUUUAU...........................................................      21      7
...........................................UCAGCGGAUAACAUGUUUUAUU..........................................................      22      7
.................................................................................ACCAGUGACCAGACAUAUCCCU....................      22      5
..................................................................................CCAGUGACCAGACAUAUC.......................      18      17
..................................................................................CCAGUGACCAGACAUAUCC......................      19      41
..................................................................................CCAGUGACCAGACAUAUCCC.....................      20      266
..................................................................................CCAGUGACCAGACAUAUCCCU....................      21      2226
..................................................................................CCAGUGACCAGACAUAUCCCUA...................      22      2359
..................................................................................CCAGUGACCAGACAUAUCCCUAU..................      23      24
...................................................................................CAGUGACCAGACAUAUCC......................      18      39
...................................................................................CAGUGACCAGACAUAUCCC.....................      19      129
...................................................................................CAGUGACCAGACAUAUCCCU....................      20      4268
...................................................................................CAGUGACCAGACAUAUCCCUA...................      21      2118
...................................................................................CAGUGACCAGACAUAUCCCUAU..................      22      385
....................................................................................AGUGACCAGACAUAUCCC.....................      18      29
....................................................................................AGUGACCAGACAUAUCCCU....................      19      701
....................................................................................AGUGACCAGACAUAUCCCUA...................      20      136
....................................................................................AGUGACCAGACAUAUCCCUAU..................      21      13
.....................................................................................GUGACCAGACAUAUCCCU....................      18      5
.....................................................................................GUGACCAGACAUAUCCCUA...................      19      5
antisense match


#sja-mir-1989
AGAGTTAAACGTAACTTCAGCTGTGTTCATGTCTTCGACAAAAAATAAACCAACAAGTGAATACGTAGGTATTCATGTTGATTAATATCGAAGAAATGAGCACAACTTAGATTGTTTACTCT
Total sRNA: 5787 shared 5' end with miRNA: 613     shared 3' end with miRNA: 364
miRNA
...............UCAGCUGUGUUCAUGUCUUCGA.....................................................................................      22      2812
Shared 5' Terminus
...............UCAGCUGUGUUCAUGUCU.........................................................................................      18      72
...............UCAGCUGUGUUCAUGUCUU........................................................................................      19      83
...............UCAGCUGUGUUCAUGUCUUC.......................................................................................      20      46
...............UCAGCUGUGUUCAUGUCUUCG......................................................................................      21      412
Shared 3' Terminus
.................AGCUGUGUUCAUGUCUUCGA.....................................................................................      20      219
................CAGCUGUGUUCAUGUCUUCGA.....................................................................................      21      116
..............UUCAGCUGUGUUCAUGUCUUCGA.....................................................................................      23      29
Subsequence
................CAGCUGUGUUCAUGUCUUCG......................................................................................      20      8
.................AGCUGUGUUCAUGUCUUCG......................................................................................      19      16
Others
..............UUCAGCUGUGUUCAUGUCUUCG......................................................................................      22      10
................CAGCUGUGUUCAUGUCUUCGAC....................................................................................      22      132
.................AGCUGUGUUCAUGUCUUCGAC....................................................................................      21      7
.......................................................................................UCGAAGAAAUGAGCACAACUUA.............      22      6
........................................................................................CGAAGAAAUGAGCACAACUUAG............      22      8
........................................................................................CGAAGAAAUGAGCACAACUUAGA...........      23      15
.........................................................................................GAAGAAAUGAGCACAACUU..............      19      5
.........................................................................................GAAGAAAUGAGCACAACUUA.............      20      16
.........................................................................................GAAGAAAUGAGCACAACUUAG............      21      43
.........................................................................................GAAGAAAUGAGCACAACUUAGA...........      22      1579
..........................................................................................AAGAAAUGAGCACAACUUA.............      19      12
..........................................................................................AAGAAAUGAGCACAACUUAG............      20      52
..........................................................................................AAGAAAUGAGCACAACUUAGA...........      21      60
antisense match


#sja-mir-1b
NNTGGAATGTTGTGAAGTATGTGCNN
Total sRNA: 1471286       shared 5' end with miRNA: 56390    shared 3' end with miRNA: 2520
miRNA
..UGGAAUGUUGUGAAGUAUGUGC..        22      1412190
Shared 5' Terminus
..UGGAAUGUUGUGAAGUAU......        18      643
..UGGAAUGUUGUGAAGUAUG.....        19      2545
..UGGAAUGUUGUGAAGUAUGU....        20      10501
..UGGAAUGUUGUGAAGUAUGUG...        21      42701
Shared 3' Terminus
```

```
.....AAUGUUGUGAAGUAUGUGC..        19    12
....GAAUGUUGUGAAGUAUGUGC..        20    328
...GGAAUGUUGUGAAGUAUGUGC..        21    2177
Subsequence
...GGAAUGUUGUGAAGUAUG.....        18    35
...GGAAUGUUGUGAAGUAUGU....        19    22
...GGAAUGUUGUGAAGUAUGUG...        20    89
....GAAUGUUGUGAAGUAUGUG...        19    32
.....AAUGUUGUGAAGUAUGUG...        18    5
Others
antisense match


#sja-mir-2162
CTAGGGGATGAGTGAGATTGTTGCATATTTACATTGCTGGTATGTATTATGCAACGTTTCACTCTACTCCGGGGA
Total sRNA: 278963        shared 5' end with miRNA: 77313    shared 3' end with miRNA: 733
miRNA
............................................UAUUAUGCAACGUUUCACUCU..........  21    125057
Shared 5' Terminus
............................................UAUUAUGCAACGUUUCAC.............  18    1297
............................................UAUUAUGCAACGUUUCACU............  19    41631
............................................UAUUAUGCAACGUUUCACUC...........  20    34206
............................................UAUUAUGCAACGUUUCACUCUA.........  22    178
Shared 3' Terminus
...............................................UAUGCAACGUUUCACUCU..........  18    43
..............................................UUAUGCAACGUUUCACUCU..........  19    75
.............................................AUUAUGCAACGUUUCACUCU..........  20    606
...........................................GUAUUAUGCAACGUUUCACUCU..........  22    6
Subsequence
.............................................AUUAUGCAACGUUUCACU............  18    116
.............................................AUUAUGCAACGUUUCACUC...........  19    99
..............................................UUAUGCAACGUUUCACUC...........  18    9
Others
..........AGUGAGAUUGUUGCAUAU...............................................  18    26
..........AGUGAGAUUGUUGCAUAUU..............................................  19    67
..........AGUGAGAUUGUUGCAUAUUU.............................................  20    5327
..........AGUGAGAUUGUUGCAUAUUUA............................................  21    27610
..........AGUGAGAUUGUUGCAUAUUUAC...........................................  22    3818
..........AGUGAGAUUGUUGCAUAUUUACA..........................................  23    31490
..........AGUGAGAUUGUUGCAUAUUUACAU.........................................  24    174
..........AGUGAGAUUGUUGCAUAUUUACAUU........................................  25    94
...........GUGAGAUUGUUGCAUAUUU.............................................  19    134
...........GUGAGAUUGUUGCAUAUUUA............................................  20    2473
...........GUGAGAUUGUUGCAUAUUUAC...........................................  21    2605
...........GUGAGAUUGUUGCAUAUUUACA..........................................  22    1658
...........GUGAGAUUGUUGCAUAUUUACAU.........................................  23    23
...........GUGAGAUUGUUGCAUAUUUACAUU........................................  24    19
............UGAGAUUGUUGCAUAUUUAC...........................................  20    7
............UGAGAUUGUUGCAUAUUUACA..........................................  21    34
.............GAGAUUGUUGCAUAUUUAC...........................................  19    5
.............GAGAUUGUUGCAUAUUUACA..........................................  20    34
.............GAGAUUGUUGCAUAUUUACAUU........................................  22    9
antisense match


#sja-mir-219
TCTGTGAACAATCGATTCACTGATTGTCCATTCGCATTTCTTGTTAGAAACATTTTCAATTATTATCATTATTCAATGATAATGATGATAATATTTAATTCACAAGAGGTGTTAATGGACATCATATGATCGATTATTAATCATCT
Total sRNA: 102776        shared 5' end with miRNA: 44799    shared 3' end with miRNA: 2507
miRNA
....................UGAUUGUCCAUUCGCAUUUCUU........................................................................................................        22    50730
Shared 5' Terminus
....................UGAUUGUCCAUUCGCAUU............................................................................................................        18    80
....................UGAUUGUCCAUUCGCAUUU...........................................................................................................        19    309
....................UGAUUGUCCAUUCGCAUUUC..........................................................................................................        20    411
....................UGAUUGUCCAUUCGCAUUUCU.........................................................................................................        21    7016
....................UGAUUGUCCAUUCGCAUUUCUUG.......................................................................................................        23    36501
....................UGAUUGUCCAUUCGCAUUUCUUGU......................................................................................................        24    464
....................UGAUUGUCCAUUCGCAUUUCUUGUU.....................................................................................................        25    18
Shared 3' Terminus
```

```
......................AUUGUCCAUUCGCAUUUCUU........................................................................................................    20    55
.....................GAUUGUCCAUUCGCAUUUCUU........................................................................................................    21    2441
...................CUGAUUGUCCAUUCGCAUUUCUU........................................................................................................    23    8
Subsequence
.....................GAUUGUCCAUUCGCAUUU...........................................................................................................    18    7
.....................GAUUGUCCAUUCGCAUUUC..........................................................................................................    19    13
.....................GAUUGUCCAUUCGCAUUUCU.........................................................................................................    20    203
......................AUUGUCCAUUCGCAUUUCU.........................................................................................................    19    6
Others
...................CUGAUUGUCCAUUCGCAUUUCUUG.......................................................................................................    24    11
.....................GAUUGUCCAUUCGCAUUUCUUG.......................................................................................................    22    3038
.....................GAUUGUCCAUUCGCAUUUCUUGU......................................................................................................    23    55
......................AUUGUCCAUUCGCAUUUCUUG.......................................................................................................    21    165
......................AUUGUCCAUUCGCAUUUCUUGU......................................................................................................    22    115
.......................UUGUCCAUUCGCAUUUCUUG.......................................................................................................    20    16
.......................UUGUCCAUUCGCAUUUCUUGU......................................................................................................    21    102
.......................UUGUCCAUUCGCAUUUCUUGUU.....................................................................................................    22    18
.........................GUCCAUUCGCAUUUCUUG.......................................................................................................    18    12
.......................................................................................................AAGAGGUGUUAAUGGACAU........................    19    5
.......................................................................................................AAGAGGUGUUAAUGGACAUCAU.....................    22    40
........................................................................................................AGAGGUGUUAAUGGACAUC.......................    19    5
........................................................................................................AGAGGUGUUAAUGGACAUCA......................    20    9
........................................................................................................AGAGGUGUUAAUGGACAUCAU.....................    21    318
........................................................................................................AGAGGUGUUAAUGGACAUCAUA....................    22    573
antisense match


#sja-mir-277
ATAANNNNNNNNNNATATTGTAAATGCATTTTCTGGCCCGTAACTAAGACACAATGAACATCCTTATCTTTGATAGTTTTTAAAAGTTAAGTTTTGTTTTGTCTTGTCAATGTAATAGATGTCCTTTCTTTTCAGTGATGGG
Total sRNA: 85658shared 5' end with miRNA: 54014   shared 3' end with miRNA: 396
miRNA
....................UAAAUGCAUUUUCUGGCCCGU.....................................................................................................   21    28896
Shared 5' Terminus
....................UAAAUGCAUUUUCUGGCC........................................................................................................   18    965
....................UAAAUGCAUUUUCUGGCCC.......................................................................................................   19    2242
....................UAAAUGCAUUUUCUGGCCCG......................................................................................................   20    2116
....................UAAAUGCAUUUUCUGGCCCGUA....................................................................................................   22    24811
....................UAAAUGCAUUUUCUGGCCCGUAA...................................................................................................   23    23471
....................UAAAUGCAUUUUCUGGCCCGUAAC..................................................................................................   24    330
....................UAAAUGCAUUUUCUGGCCCGUAACU.................................................................................................   25    75
Shared 3' Terminus
.......................AUGCAUUUUCUGGCCCGU.....................................................................................................   18    7
......................AAUGCAUUUUCUGGCCCGU.....................................................................................................   19    109
.....................AAAUGCAUUUUCUGGCCCGU.....................................................................................................   20    279
Subsequence
.....................AAAUGCAUUUUCUGGCCC.......................................................................................................   18    16
.....................AAAUGCAUUUUCUGGCCCG......................................................................................................   19    18
......................AAUGCAUUUUCUGGCCCG......................................................................................................   18    13
Others
.....................AAAUGCAUUUUCUGGCCCGUA....................................................................................................   21    326
.....................AAAUGCAUUUUCUGGCCCGUAA...................................................................................................   22    535
.....................AAAUGCAUUUUCUGGCCCGUAAC..................................................................................................   23    37
.....................AAAUGCAUUUUCUGGCCCGUAACU.................................................................................................   24    32
......................AAUGCAUUUUCUGGCCCGUA....................................................................................................   20    213
......................AAUGCAUUUUCUGGCCCGUAA...................................................................................................   21    434
......................AAUGCAUUUUCUGGCCCGUAAC..................................................................................................   22    428
......................AAUGCAUUUUCUGGCCCGUAACU.................................................................................................   23    263
.......................AUGCAUUUUCUGGCCCGUA....................................................................................................   19    14
.......................AUGCAUUUUCUGGCCCGUAA...................................................................................................   20    9
.........................GCAUUUUCUGGCCCGUAA...................................................................................................   18    10
antisense match


#sja-mir-277b
NNAAAATGCATCATCTACCCTAGANN
Total sRNA: 328649       shared 5' end with miRNA: 119667  shared 3' end with miRNA: 1349
miRNA
..AAAAUGCAUCAUCUACCCUAGA..        22      207027
Shared 5' Terminus
```

```
..AAAAUGCAUCAUCUACCC......        18      576
..AAAAUGCAUCAUCUACCCU.....        19      3094
..AAAAUGCAUCAUCUACCCUA....        20      15059
..AAAAUGCAUCAUCUACCCUAG...        21      100938
Shared 3' Terminus
......UGCAUCAUCUACCCUAGA..        18      20
.....AUGCAUCAUCUACCCUAGA..        19      75
....AAUGCAUCAUCUACCCUAGA..        20      112
...AAAUGCAUCAUCUACCCUAGA..        21      1142
Subsequence
...AAAUGCAUCAUCUACCCU.....        18      10
...AAAUGCAUCAUCUACCCUA....        19      60
...AAAUGCAUCAUCUACCCUAG...        20      460
....AAUGCAUCAUCUACCCUA....        18      6
....AAUGCAUCAUCUACCCUAG...        19      43
.....AUGCAUCAUCUACCCUAG...        18      27
Others
antisense match


#sja-mir-2a
ACCTTTTGCACCGCAGTCAATATTGGCTGATGGCATTGTTTTATTGTCACAGCCAGTATTGATGAACGGGGTAATAGG
Total sRNA: 932498        shared 5' end with miRNA: 72273   shared 3' end with miRNA: 9480
miRNA
..............................................UCACAGCCAGUAUUGAUGAACG..........        22      850364
Shared 5' Terminus
..............................................UCACAGCCAGUAUUGAUG..............        18      2359
..............................................UCACAGCCAGUAUUGAUGA.............        19      6905
..............................................UCACAGCCAGUAUUGAUGAA............        20      24093
..............................................UCACAGCCAGUAUUGAUGAAC...........        21      38862
..............................................UCACAGCCAGUAUUGAUGAACGG.........        23      40
..............................................UCACAGCCAGUAUUGAUGAACGGGG.......        25      8
Shared 3' Terminus
..................................................AGCCAGUAUUGAUGAACG..........        18      20
.................................................CAGCCAGUAUUGAUGAACG..........        19      20
................................................ACAGCCAGUAUUGAUGAACG..........        20      7479
...............................................CACAGCCAGUAUUGAUGAACG..........        21      1960
Subsequence
...............................................CACAGCCAGUAUUGAUGA.............        18      15
...............................................CACAGCCAGUAUUGAUGAA............        19      64
...............................................CACAGCCAGUAUUGAUGAAC...........        20      66
................................................ACAGCCAGUAUUGAUGAA............        18      44
................................................ACAGCCAGUAUUGAUGAAC...........        19      105
Others
.............CAGUCAAUAUUGGCUGAUGGCA...........................................        22      44
.............CAGUCAAUAUUGGCUGAUGGCAU..........................................        23      5
..............AGUCAAUAUUGGCUGAUGGCA...........................................        21      10
..............AGUCAAUAUUGGCUGAUGGCAU..........................................        22      7
.............................................GUCACAGCCAGUAUUGAUGAAC...........        22      7
antisense match


#sja-mir-2b
AGGTGGTGCGCGTCTCAAAGGACTGTGAGCCAACGTAATTACTGTATCACAGCCCTGCTTGGGACACAGCCTACCTGC
Total sRNA: 16860shared 5' end with miRNA: 10920   shared 3' end with miRNA: 50
miRNA
............................................UAUCACAGCCCUGCUUGGGACAC...........        23      5629
Shared 5' Terminus
............................................UAUCACAGCCCUGCUUGG................        18      102
............................................UAUCACAGCCCUGCUUGGG...............        19      209
............................................UAUCACAGCCCUGCUUGGGA..............        20      448
............................................UAUCACAGCCCUGCUUGGGAC.............        21      1043
............................................UAUCACAGCCCUGCUUGGGACA............        22      5295
............................................UAUCACAGCCCUGCUUGGGACACA..........        24      3822
Shared 3' Terminus
................................................ACAGCCCUGCUUGGGACAC...........        19      30
.............................................AUCACAGCCCUGCUUGGGACAC...........        22      16
Subsequence
.............................................AUCACAGCCCUGCUUGGGACA............        21      8
```

```
................................................ACAGCCCUGCUUGGGACA............        18      11
Others
..........CGUCUCAAAGGACUGUGAG.................................................        19      6
..........CGUCUCAAAGGACUGUGAGC................................................        20      9
..........CGUCUCAAAGGACUGUGAGCC...............................................        21      19
..........CGUCUCAAAGGACUGUGAGCCA..............................................        22      92
...........GUCUCAAAGGACUGUGAGCC...............................................        20      10
...........GUCUCAAAGGACUGUGAGCCA..............................................        21      20
...........GUCUCAAAGGACUGUGAGCCAAC............................................        23      5
.............................................AUCACAGCCCUGCUUGGGACACA..........        23      21
................................................ACAGCCCUGCUUGGGACACA..........        20      41
antisense match


#sja-mir-2c
TTCAGGCGCAACCCTTGTTCGACTGTGATGTGATTGAAATTAGCCCATATCACAGCCGTGCTTAAGGGCTTCGTCGAAA
Total sRNA: 6498 shared 5' end with miRNA: 944     shared 3' end with miRNA: 23
miRNA
..........ACCCUUGUUCGACUGUGAUGUG...............................................       22      4523
Shared 5' Terminus
..........ACCCUUGUUCGACUGUGAU..................................................       19      6
..........ACCCUUGUUCGACUGUGAUG.................................................       20      23
..........ACCCUUGUUCGACUGUGAUGU................................................       21      899
..........ACCCUUGUUCGACUGUGAUGUGA..............................................       23      11
Shared 3' Terminus
............CCUUGUUCGACUGUGAUGUG...............................................       20      6
...........CCCUUGUUCGACUGUGAUGUG...............................................       21      14
Subsequence
Others
...............................................UAUCACAGCCGUGCUUAAG.............       19      24
...............................................UAUCACAGCCGUGCUUAAGG............       20      175
...............................................UAUCACAGCCGUGCUUAAGGG...........       21      126
...............................................UAUCACAGCCGUGCUUAAGGGC..........       22      204
...............................................UAUCACAGCCGUGCUUAAGGGCU.........       23      240
...............................................UAUCACAGCCGUGCUUAAGGGCUU........       24      206
................................................AUCACAGCCGUGCUUAAGGGCUU........       23      6
antisense match


#sja-mir-2d
CGACCAGTTCGTCATCCATGGATTGTGATTTTCGGACTCCATGTTTCATTAAGTGAATATCACAGTCCTGCTTAGGTGACGAATTTCGT
Total sRNA: 45601shared 5' end with miRNA: 20296   shared 3' end with miRNA: 221
miRNA
.........................................................UAUCACAGUCCUGCUUAGGUGACG........      24      24427
Shared 5' Terminus
.........................................................UAUCACAGUCCUGCUUAG..............      18      10
.........................................................UAUCACAGUCCUGCUUAGG.............      19      50
.........................................................UAUCACAGUCCUGCUUAGGU............      20      147
.........................................................UAUCACAGUCCUGCUUAGGUG...........      21      141
.........................................................UAUCACAGUCCUGCUUAGGUGA..........      22      3505
.........................................................UAUCACAGUCCUGCUUAGGUGAC.........      23      5292
.........................................................UAUCACAGUCCUGCUUAGGUGACGA.......      25      10898
.........................................................UAUCACAGUCCUGCUUAGGUGACGAA......      26      243
.........................................................UAUCACAGUCCUGCUUAGGUGACGAAU.....      27      8
Shared 3' Terminus
.............................................................ACAGUCCUGCUUAGGUGACG........      20      19
...........................................................UCACAGUCCUGCUUAGGUGACG........      22      7
..........................................................AUCACAGUCCUGCUUAGGUGACG........      23      42
........................................................AUAUCACAGUCCUGCUUAGGUGACG........      25      153
Subsequence
..........................................................AUCACAGUCCUGCUUAGGUGA..........      21      6
..........................................................AUCACAGUCCUGCUUAGGUGAC.........      22      10
Others
..........GUCAUCCAUGGAUUGUGAUUU..........................................................      21      17
..........GUCAUCCAUGGAUUGUGAUUUU.........................................................      22      287
............CAUCCAUGGAUUGUGAUUUU.........................................................      20      9
................................CGGACUCCAUGUUUCAUUAA.....................................      20      5
................................CGGACUCCAUGUUUCAUUAAG....................................      21      6
................................CGGACUCCAUGUUUCAUUAAGU...................................      22      33
```

```
................................CGGACUCCAUGUUUCAUUAAGUG..................................   23   7
................................CGGACUCCAUGUUUCAUUAAGUGA.................................   24   33
................................CGGACUCCAUGUUUCAUUAAGUGAA................................   25   34
........................................................AUAUCACAGUCCUGCUUAGGU............   21   10
........................................................AUAUCACAGUCCUGCUUAGGUG...........   22   10
........................................................AUAUCACAGUCCUGCUUAGGUGA..........   23   40
........................................................AUAUCACAGUCCUGCUUAGGUGAC.........   24   42
........................................................AUAUCACAGUCCUGCUUAGGUGACGA.......   26   47
..........................................................AUCACAGUCCUGCUUAGGUGACGA.......   24   23
.............................................................ACAGUCCUGCUUAGGUGACGA.......   21   9
antisense match
```

```
#sja-mir-2e
CCACCGCTCTTACCAACTTAGACTGAGTTATACTGCTACTGTAAGCTTTGTATATCACAGTCCAAGCTTTGGTAAGTTTTGAT
Total sRNA: 16624shared 5' end with miRNA: 5958    shared 3' end with miRNA: 22
miRNA
....................................................UAUCACAGUCCAAGCUUUGGU..........   21   5987
Shared 5' Terminus
....................................................UAUCACAGUCCAAGCUUU.............   18   720
....................................................UAUCACAGUCCAAGCUUUG............   19   182
....................................................UAUCACAGUCCAAGCUUUGG...........   20   1001
....................................................UAUCACAGUCCAAGCUUUGGUA.........   22   786
....................................................UAUCACAGUCCAAGCUUUGGUAA........   23   2686
....................................................UAUCACAGUCCAAGCUUUGGUAAG.......   24   560
....................................................UAUCACAGUCCAAGCUUUGGUAAGU......   25   23
Shared 3' Terminus
.....................................................AUCACAGUCCAAGCUUUGGU..........   20   17
Subsequence
Others
..........UACCAACUUAGACUGAGUUA.....................................................   20   56
..........UACCAACUUAGACUGAGUUAU....................................................   21   3524
..........UACCAACUUAGACUGAGUUAUA...................................................   22   596
...........ACCAACUUAGACUGAGUUA.....................................................   19   11
...........ACCAACUUAGACUGAGUUAU....................................................   20   53
...........ACCAACUUAGACUGAGUUAUA...................................................   21   85
............CCAACUUAGACUGAGUUAU....................................................   19   5
................................CUGCUACUGUAAGCUUUGUA...............................   20   10
..................................................AUAUCACAGUCCAAGCUUUGGUAA.........   24   5
.....................................................AUCACAGUCCAAGCUUUGGUA.........   21   49
.....................................................AUCACAGUCCAAGCUUUGGUAA........   22   144
.....................................................AUCACAGUCCAAGCUUUGGUAAG.......   23   19
......................................................UCACAGUCCAAGCUUUGGUAA........   21   12
......................................................UCACAGUCCAAGCUUUGGUAAGU......   23   9
........................................................ACAGUCCAAGCUUUGGUA.........   18   7
........................................................ACAGUCCAAGCUUUGGUAA........   19   16
antisense match
```

```
#sja-mir-307
AGAAACTCCTCATAAAGAAGGTTGTTTGATGTAATATTCAATAATTAATAATAATTATTATTATTATTATTATCATTTCGATGAAATAATAATATAAAGAAGATGAATATAAAATAACTTACATCACAACCTACTTGATTGAGGGGCACTTCG
Total sRNA: 2800 shared 5' end with miRNA: 221     shared 3' end with miRNA: 64
miRNA
.......CCUCAUAAAGAAGGUUGUUUGAUG.......................................................................................................................... 24   757
Shared 5' Terminus
.......CCUCAUAAAGAAGGUUGUUUG............................................................................................................................. 21   7
.......CCUCAUAAAGAAGGUUGUUUGA............................................................................................................................ 22   43
.......CCUCAUAAAGAAGGUUGUUUGAU........................................................................................................................... 23   40
.......CCUCAUAAAGAAGGUUGUUUGAUGU......................................................................................................................... 25   119
.......CCUCAUAAAGAAGGUUGUUUGAUGUA........................................................................................................................ 26   10
Shared 3' Terminus
...........AUAAAGAAGGUUGUUUGAUG.......................................................................................................................... 20   10
.........UCAUAAAGAAGGUUGUUUGAUG.......................................................................................................................... 22   15
........CUCAUAAAGAAGGUUGUUUGAUG.......................................................................................................................... 23   27
......UCCUCAUAAAGAAGGUUGUUUGAUG.......................................................................................................................... 25   8
Subsequence
........CUCAUAAAGAAGGUUGUUUGA............................................................................................................................ 21   13
........CUCAUAAAGAAGGUUGUUUGAU........................................................................................................................... 22   11
.........UCAUAAAGAAGGUUGUUUGAU........................................................................................................................... 21   6
```

```
Others
........CUCAUAAAGAAGGUUGUUUGAUGU........................................................................................................................  24      9
..........................................................................................................................AUCACAACCUACUUGAUUGA..........  20      8
..........................................................................................................................AUCACAACCUACUUGAUUGAG.........  21      11
..........................................................................................................................AUCACAACCUACUUGAUUGAGGGG......  24      12
...........................................................................................................................UCACAACCUACUUGAUUG...........  18      9
...........................................................................................................................UCACAACCUACUUGAUUGA..........  19      212
...........................................................................................................................UCACAACCUACUUGAUUGAG.........  20      273
...........................................................................................................................UCACAACCUACUUGAUUGAGG........  21      189
...........................................................................................................................UCACAACCUACUUGAUUGAGGG.......  22      214
...........................................................................................................................UCACAACCUACUUGAUUGAGGGG......  23      616
............................................................................................................................CACAACCUACUUGAUUGAGGGG......  22      6
.............................................................................................................................ACAACCUACUUGAUUGAG.........  18      11
.............................................................................................................................ACAACCUACUUGAUUGAGG........  19      17
.............................................................................................................................ACAACCUACUUGAUUGAGGG.......  20      15
.............................................................................................................................ACAACCUACUUGAUUGAGGGG......  21      89
antisense match


#sja-mir-31
TATTTGTTGTTGGCAAGATTACGGCGAAGCTGAATCAAATTTAAAATGTTTCAGCTTCACTTTATTTTGCCAATAAAGGTAGC
Total sRNA: 2133 shared 5' end with miRNA: 1104    shared 3' end with miRNA: 9
miRNA
..........UGGCAAGAUUACGGCGAAGCU....................................................   21      993
Shared 5' Terminus
..........UGGCAAGAUUACGGCGAAG......................................................   19      129
..........UGGCAAGAUUACGGCGAAGC.....................................................   20      130
..........UGGCAAGAUUACGGCGAAGCUG...................................................   22      172
..........UGGCAAGAUUACGGCGAAGCUGA..................................................   23      618
..........UGGCAAGAUUACGGCGAAGCUGAA.................................................   24      48
Shared 3' Terminus
...........GGCAAGAUUACGGCGAAGCU....................................................   20      7
Subsequence
Others
...........GGCAAGAUUACGGCGAAGCUGAA.................................................   23      11
....................................................AGCUUCACUUUAUUUUGCCAAU.........   22      6
antisense match


#sja-mir-3479
CATGTTTGTGAGGTGGATGTTAGTGCAATTTAGTGACGTAAATATATTGCACTTACCTTCGCCTTGCAAATATAGG
Total sRNA: 47531shared 5' end with miRNA: 7136    shared 3' end with miRNA: 187
miRNA
............................................UAUUGCACUUACCUUCGCCUUG.......... 22      32829
Shared 5' Terminus
............................................UAUUGCACUUACCUUCGC.............. 18      216
............................................UAUUGCACUUACCUUCGCC............. 19      870
............................................UAUUGCACUUACCUUCGCCU............ 20      1196
............................................UAUUGCACUUACCUUCGCCUU........... 21      4787
............................................UAUUGCACUUACCUUCGCCUUGC......... 23      67
Shared 3' Terminus
..............................................UUGCACUUACCUUCGCCUUG.......... 20      41
.............................................AUUGCACUUACCUUCGCCUUG.......... 21      104
...........................................AUAUUGCACUUACCUUCGCCUUG.......... 23      33
Subsequence
.............................................AUUGCACUUACCUUCGCC............. 18      5
.............................................AUUGCACUUACCUUCGCCUU........... 20      25
..............................................UUGCACUUACCUUCGCCUU........... 19      7
Others
.....UUGUGAGGUGGAUGUUAGUGCA................................................. 22      10
........UGAGGUGGAUGUUAGUGCAAUU.............................................. 22      97
........UGAGGUGGAUGUUAGUGCAAUUU............................................. 23      8
.........GAGGUGGAUGUUAGUGCAAU............................................... 20      11
.........GAGGUGGAUGUUAGUGCAAUU.............................................. 21      70
.........GAGGUGGAUGUUAGUGCAAUUU............................................. 22      369
..........AGGUGGAUGUUAGUGCAA................................................ 18      9
..........AGGUGGAUGUUAGUGCAAU............................................... 19      22
..........AGGUGGAUGUUAGUGCAAUU.............................................. 20      267
..........AGGUGGAUGUUAGUGCAAUUU............................................. 21      1336
```

```
..........AGGUGGAUGUUAGUGCAAUUUA............................................ 22      4674
...........GGUGGAUGUUAGUGCAAUUU............................................. 20      15
...........GGUGGAUGUUAGUGCAAUUUA............................................ 21      214
............GUGGAUGUUAGUGCAAUUUA............................................ 20      98
.............................................AUUGCACUUACCUUCGCCUUGC......... 22      80
..............................................UUGCACUUACCUUCGCCUUGC......... 21      9
...............................................UGCACUUACCUUCGCCUUGC......... 20      5
antisense match


#sja-mir-3488
TGGTGAATGACGATTGATGATTATAATGGCGCGCACGTGCTCCGGTAGCTTAGTTGGTTAGAGCGCCG
Total sRNA: 2104 shared 5' end with miRNA: 1142    shared 3' end with miRNA: 29
miRNA
......................................GCUCCGGUAGCUUAGUUGGU.......... 20      653
Shared 5' Terminus
......................................GCUCCGGUAGCUUAGUUG............ 18      73
......................................GCUCCGGUAGCUUAGUUGG........... 19      186
......................................GCUCCGGUAGCUUAGUUGGUU......... 21      279
......................................GCUCCGGUAGCUUAGUUGGUUA........ 22      358
......................................GCUCCGGUAGCUUAGUUGGUUAG....... 23      173
......................................GCUCCGGUAGCUUAGUUGGUUAGA...... 24      41
......................................GCUCCGGUAGCUUAGUUGGUUAGAG..... 25      25
......................................GCUCCGGUAGCUUAGUUGGUUAGAGC.... 26      6
Shared 3' Terminus
........................................UCCGGUAGCUUAGUUGGU.......... 18      5
....................................GUGCUCCGGUAGCUUAGUUGGU.......... 22      22
Subsequence
Others
....................................GUGCUCCGGUAGCUUAGU.............. 18      7
....................................GUGCUCCGGUAGCUUAGUUG............ 20      17
....................................GUGCUCCGGUAGCUUAGUUGG........... 21      49
....................................GUGCUCCGGUAGCUUAGUUGGUU......... 23      39
....................................GUGCUCCGGUAGCUUAGUUGGUUA........ 24      42
....................................GUGCUCCGGUAGCUUAGUUGGUUAG....... 25      10
.....................................UGCUCCGGUAGCUUAGUUGGUU......... 22      5
........................................UCCGGUAGCUUAGUUGGUU......... 19      36
........................................UCCGGUAGCUUAGUUGGUUA........ 20      12
........................................UCCGGUAGCUUAGUUGGUUAG....... 21      14
.........................................CCGGUAGCUUAGUUGGUUAG....... 20      5
..........................................CGGUAGCUUAGUUGGUUAG....... 19      5
antisense match


#sja-mir-3505
TGCGCAAGTGTGACTGTCTGGACACAGTAGTTTGGCGGTAAACGCTGTGGCGTTTGACACGAAACAGACTGAGTTCCAACCGTAG
Total sRNA: 4354 shared 5' end with miRNA: 2132    shared 3' end with miRNA: 127
miRNA
..........UGACUGUCUGGACACAGUAGUUU.................................................... 23      1903
Shared 5' Terminus
..........UGACUGUCUGGACACAGUAG....................................................... 20      906
..........UGACUGUCUGGACACAGUAGU...................................................... 21      350
..........UGACUGUCUGGACACAGUAGUU..................................................... 22      873
Shared 3' Terminus
...........GACUGUCUGGACACAGUAGUUU.................................................... 22      40
.........GUGACUGUCUGGACACAGUAGUUU.................................................... 24      87
Subsequence
...........GACUGUCUGGACACAGUAG....................................................... 19      72
...........GACUGUCUGGACACAGUAGU...................................................... 20      13
...........GACUGUCUGGACACAGUAGUU..................................................... 21      5
Others
....CAAGUGUGACUGUCUGGACA............................................................. 20      15
.....AAGUGUGACUGUCUGGACAC............................................................ 20      8
......AGUGUGACUGUCUGGACACA........................................................... 20      11
.......GUGUGACUGUCUGGACACAG.......................................................... 20      12
........UGUGACUGUCUGGACACAGUAG....................................................... 22      7
.........GUGACUGUCUGGACACAGUA........................................................ 20      11
.........GUGACUGUCUGGACACAGUAG....................................................... 21      12
.........GUGACUGUCUGGACACAGUAGUU..................................................... 23      9
```

```
antisense match


#sja-mir-36
TATAGCTTGCGAAGAATGCCGATCCGGTCAGCCATTCAACAATCTATCAGTAATAAACGGCCACCGGGTAGACATTCATTCGCAAGATATTAT
Total sRNA: 115809        shared 5' end with miRNA: 34241    shared 3' end with miRNA: 976
miRNA
............................................................CCACCGGGUAGACAUUCAUUCGC.......... 23      79783
Shared 5' Terminus
............................................................CCACCGGGUAGACAUUCA............... 18      114
............................................................CCACCGGGUAGACAUUCAU.............. 19      1331
............................................................CCACCGGGUAGACAUUCAUU............. 20      1280
............................................................CCACCGGGUAGACAUUCAUUC............ 21      5497
............................................................CCACCGGGUAGACAUUCAUUCG........... 22      15942
............................................................CCACCGGGUAGACAUUCAUUCGCA......... 24      9479
............................................................CCACCGGGUAGACAUUCAUUCGCAA........ 25      596
Shared 3' Terminus
.................................................................GGGUAGACAUUCAUUCGC.......... 18      108
...............................................................CCGGGUAGACAUUCAUUCGC.......... 20      10
..............................................................ACCGGGUAGACAUUCAUUCGC.......... 21      108
.............................................................CACCGGGUAGACAUUCAUUCGC.......... 22      748
Subsequence
.............................................................CACCGGGUAGACAUUCAU.............. 18      9
.............................................................CACCGGGUAGACAUUCAUUC............ 20      33
.............................................................CACCGGGUAGACAUUCAUUCG........... 21      148
..............................................................ACCGGGUAGACAUUCAUUCG........... 20      26
Others
..........GAAGAAUGCCGAUCCGGUCA............................................................... 20      8
..........GAAGAAUGCCGAUCCGGUCAG.............................................................. 21      5
..........GAAGAAUGCCGAUCCGGUCAGC............................................................. 22      25
..........GAAGAAUGCCGAUCCGGUCAGCC............................................................ 23      275
...........AAGAAUGCCGAUCCGGUCAGCC............................................................ 22      19
.................................AUUCAACAAUCUAUCAGUAAUAAACGG................................. 27      13
.....................................AACAAUCUAUCAGUAAUAAACGG................................. 23      6
.............................................................CACCGGGUAGACAUUCAUUCGCA......... 23      160
.............................................................CACCGGGUAGACAUUCAUUCGCAA........ 24      18
..............................................................ACCGGGUAGACAUUCAUUCGCA......... 22      15
.................................................................GGGUAGACAUUCAUUCGCA......... 19      6
antisense match


#sja-mir-61
CATTCTAACTTTACATCACATGACTAGAAAGTGCACTCACTTCCATCTCAGTACAGAGCATCATTTAGAGAAATGTGTCCACAGTAAAAATTCCCTATGTTACGTAAATTTGCGTAAGTTCAATGTGTATTGTAGATTTATTA
Total sRNA: 47544shared 5' end with miRNA: 21747    shared 3' end with miRNA: 147
miRNA
....................UGACUAGAAAGUGCACUCACUUC....................................................................................................    23      25522
Shared 5' Terminus
....................UGACUAGAAAGUGCACUC.........................................................................................................    18      30
....................UGACUAGAAAGUGCACUCA........................................................................................................    19      99
....................UGACUAGAAAGUGCACUCAC.......................................................................................................    20      270
....................UGACUAGAAAGUGCACUCACU......................................................................................................    21      4638
....................UGACUAGAAAGUGCACUCACUU.....................................................................................................    22      16667
....................UGACUAGAAAGUGCACUCACUUCC...................................................................................................    24      43
Shared 3' Terminus
......................ACUAGAAAGUGCACUCACUUC....................................................................................................    21      18
.....................GACUAGAAAGUGCACUCACUUC....................................................................................................    22      115
...................AUGACUAGAAAGUGCACUCACUUC....................................................................................................    24      6
Subsequence
.....................GACUAGAAAGUGCACUCACU......................................................................................................    20      13
.....................GACUAGAAAGUGCACUCACUU.....................................................................................................    21      43
......................ACUAGAAAGUGCACUCACUU.....................................................................................................    20      12
Others
...............UCACAUGACUAGAAAGUGCACU..........................................................................................................    22      36
...............UCACAUGACUAGAAAGUGCACUC.........................................................................................................    23      9
antisense match


#sja-mir-7
ATGTTGCCGTTGCTTAATCATGGAAGACTGGTGATATGTTGTTGATTGATGAAAGTTTTAAGTATTTCATAATTTAAAGCTTCATCAAAACAATGATATTACGAACAACTTATCGCATTCTTCCATGTATGCAATAGGTTGAACTTC
```

```
Total sRNA: 1449744       shared 5' end with miRNA: 170273  shared 3' end with miRNA: 5639
miRNA
....................UGGAAGACUGGUGAUAUGUUGUU........................................................................................................      23      1271095
Shared 5' Terminus
....................UGGAAGACUGGUGAUAUG.............................................................................................................      18      677
....................UGGAAGACUGGUGAUAUGU............................................................................................................      19      2134
....................UGGAAGACUGGUGAUAUGUU...........................................................................................................      20      5361
....................UGGAAGACUGGUGAUAUGUUG..........................................................................................................      21      9842
....................UGGAAGACUGGUGAUAUGUUGU.........................................................................................................      22      152128
....................UGGAAGACUGGUGAUAUGUUGUUG.......................................................................................................      24      130
Shared 3' Terminus
........................AGACUGGUGAUAUGUUGUU........................................................................................................      19      8
.......................AAGACUGGUGAUAUGUUGUU........................................................................................................      20      45
......................GAAGACUGGUGAUAUGUUGUU........................................................................................................      21      453
.....................GGAAGACUGGUGAUAUGUUGUU........................................................................................................      22      5044
...................AUGGAAGACUGGUGAUAUGUUGUU........................................................................................................      24      79
..................CAUGGAAGACUGGUGAUAUGUUGUU........................................................................................................      25      5
.................UCAUGGAAGACUGGUGAUAUGUUGUU........................................................................................................      26      5
Subsequence
.....................GGAAGACUGGUGAUAUGU............................................................................................................      18      9
.....................GGAAGACUGGUGAUAUGUU...........................................................................................................      19      13
.....................GGAAGACUGGUGAUAUGUUG..........................................................................................................      20      24
.....................GGAAGACUGGUGAUAUGUUGU.........................................................................................................      21      587
......................GAAGACUGGUGAUAUGUUGU.........................................................................................................      20      24
Others
.................UCAUGGAAGACUGGUGAUAUGU............................................................................................................      22      24
.................UCAUGGAAGACUGGUGAUAUGUU...........................................................................................................      23      134
.................UCAUGGAAGACUGGUGAUAUGUUG..........................................................................................................      24      7
.................UCAUGGAAGACUGGUGAUAUGUUGU.........................................................................................................      25      12
..................CAUGGAAGACUGGUGAUAUGUU...........................................................................................................      22      5
..................CAUGGAAGACUGGUGAUAUGUUG..........................................................................................................      23      5
..................CAUGGAAGACUGGUGAUAUGUUGU.........................................................................................................      24      7
...................AUGGAAGACUGGUGAUAUGUU...........................................................................................................      21      18
...................AUGGAAGACUGGUGAUAUGUUG..........................................................................................................      22      46
...................AUGGAAGACUGGUGAUAUGUUGU.........................................................................................................      23      221
.........................................................................................................CAACUUAUCGCAUUCUUCCA......................      20      40
.........................................................................................................CAACUUAUCGCAUUCUUCCAU.....................      21      153
.........................................................................................................CAACUUAUCGCAUUCUUCCAUG....................      22      795
.........................................................................................................CAACUUAUCGCAUUCUUCCAUGU...................      23      20
..........................................................................................................AACUUAUCGCAUUCUUCCA......................      19      13
..........................................................................................................AACUUAUCGCAUUCUUCCAU.....................      20      130
..........................................................................................................AACUUAUCGCAUUCUUCCAUG....................      21      354
..........................................................................................................AACUUAUCGCAUUCUUCCAUGU...................      22      56
...........................................................................................................ACUUAUCGCAUUCUUCCAUG....................      20      6
...........................................................................................................ACUUAUCGCAUUCUUCCAUGU...................      21      6
antisense match


#sja-mir-71a
GTGATACTAGTGCTGTGAAAGACGATGGTAGTGAGATGCCAGTTGCATCTCGCTTCCCCGCCTTTCCCGGTAAGAATCAC
Total sRNA: 7459724       shared 5' end with miRNA: 3604013 shared 3' end with miRNA: 9664
miRNA
...............UGAAAGACGAUGGUAGUGAGAUG..........................................      23      3835922
Shared 5' Terminus
...............UGAAAGACGAUGGUAGUG...............................................      18      9314
...............UGAAAGACGAUGGUAGUGA..............................................      19      71230
...............UGAAAGACGAUGGUAGUGAG.............................................      20      368417
...............UGAAAGACGAUGGUAGUGAGA............................................      21      615498
...............UGAAAGACGAUGGUAGUGAGAU...........................................      22      2525837
...............UGAAAGACGAUGGUAGUGAGAUGC.........................................      24      13700
...............UGAAAGACGAUGGUAGUGAGAUGCC........................................      25      16
Shared 3' Terminus
....................GACGAUGGUAGUGAGAUG..........................................      18      72
...................AGACGAUGGUAGUGAGAUG..........................................      19      124
..................AAGACGAUGGUAGUGAGAUG..........................................      20      332
.................AAAGACGAUGGUAGUGAGAUG..........................................      21      2148
................GAAAGACGAUGGUAGUGAGAUG..........................................      22      6986
Subsequence
................GAAAGACGAUGGUAGUGA..............................................      18      97
```

```
................GAAAGACGAUGGUAGUGAG.............................................   19   503
................GAAAGACGAUGGUAGUGAGA............................................   20   1295
................GAAAGACGAUGGUAGUGAGAU...........................................   21   6124
.................AAAGACGAUGGUAGUGAG.............................................   18   54
.................AAAGACGAUGGUAGUGAGA............................................   19   217
.................AAAGACGAUGGUAGUGAGAU...........................................   20   953
..................AAGACGAUGGUAGUGAGA............................................   18   20
..................AAGACGAUGGUAGUGAGAU...........................................   19   85
...................AGACGAUGGUAGUGAGAU...........................................   18   45
Others
.............UGUGAAAGACGAUGGUAGUGA..............................................   21   10
.............UGUGAAAGACGAUGGUAGUGAG.............................................   22   40
.............UGUGAAAGACGAUGGUAGUGAGA............................................   23   165
..............GUGAAAGACGAUGGUAGUGA..............................................   20   36
..............GUGAAAGACGAUGGUAGUGAG.............................................   21   36
..............GUGAAAGACGAUGGUAGUGAGA............................................   22   10
..............GUGAAAGACGAUGGUAGUGAGAU...........................................   23   362
................GAAAGACGAUGGUAGUGAGAUGC.........................................   23   16
.................AAAGACGAUGGUAGUGAGAUGC.........................................   22   12
..................AAGACGAUGGUAGUGAGAUGC.........................................   21   17
...............................................UCUCGCUUCCCCGCCUUUCCCG...........   22   10
antisense match


#sja-mir-71b
CCGTTTGCTGTGAAAGACTTGAGTAGTGAGACGCTTATAGCGTTGTTGTCTGCGGTTCGGCGCCTCATACTAAGTCTTTCCCGGCTCACTGGC
Total sRNA: 3652004       shared 5' end with miRNA: 451969   shared 3' end with miRNA: 22975
miRNA
..........UGAAAGACUUGAGUAGUGAGACG............................................................ 23   3063247
Shared 5' Terminus
..........UGAAAGACUUGAGUAGUG................................................................. 18   66
..........UGAAAGACUUGAGUAGUGA................................................................ 19   1312
..........UGAAAGACUUGAGUAGUGAG............................................................... 20   24113
..........UGAAAGACUUGAGUAGUGAGA.............................................................. 21   86355
..........UGAAAGACUUGAGUAGUGAGAC............................................................. 22   134083
..........UGAAAGACUUGAGUAGUGAGACGC........................................................... 24   161464
..........UGAAAGACUUGAGUAGUGAGACGCU.......................................................... 25   40777
..........UGAAAGACUUGAGUAGUGAGACGCUU......................................................... 26   3743
..........UGAAAGACUUGAGUAGUGAGACGCUUA........................................................ 27   47
..........UGAAAGACUUGAGUAGUGAGACGCUUAU....................................................... 28   8
Shared 3' Terminus
..............AGACUUGAGUAGUGAGACG............................................................ 19   23
.............AAGACUUGAGUAGUGAGACG............................................................ 20   429
............AAAGACUUGAGUAGUGAGACG............................................................ 21   12161
...........GAAAGACUUGAGUAGUGAGACG............................................................ 22   10338
.........GUGAAAGACUUGAGUAGUGAGACG............................................................ 24   21
Subsequence
...........GAAAGACUUGAGUAGUGAG............................................................... 19   183
...........GAAAGACUUGAGUAGUGAGA.............................................................. 20   99
...........GAAAGACUUGAGUAGUGAGAC............................................................. 21   176
............AAAGACUUGAGUAGUGAG............................................................... 18   108
............AAAGACUUGAGUAGUGAGA.............................................................. 19   196
............AAAGACUUGAGUAGUGAGAC............................................................. 20   630
.............AAGACUUGAGUAGUGAGA.............................................................. 18   5
.............AAGACUUGAGUAGUGAGAC............................................................. 19   22
Others
.........GUGAAAGACUUGAGUAGUGAGAC............................................................. 23   29
...........GAAAGACUUGAGUAGUGAGACGC........................................................... 23   613
...........GAAAGACUUGAGUAGUGAGACGCU.......................................................... 24   160
...........GAAAGACUUGAGUAGUGAGACGCUU......................................................... 25   22
............AAAGACUUGAGUAGUGAGACGC........................................................... 22   22027
............AAAGACUUGAGUAGUGAGACGCU.......................................................... 23   76622
............AAAGACUUGAGUAGUGAGACGCUU......................................................... 24   9304
............AAAGACUUGAGUAGUGAGACGCUUA........................................................ 25   99
............AAAGACUUGAGUAGUGAGACGCUUAU....................................................... 26   55
.............AAGACUUGAGUAGUGAGACGC........................................................... 21   621
.............AAGACUUGAGUAGUGAGACGCU.......................................................... 22   1581
.............AAGACUUGAGUAGUGAGACGCUU......................................................... 23   355
.............AAGACUUGAGUAGUGAGACGCUUA........................................................ 24   8
```

..............AGACUUGAGUAGUGAGACGCU.. 21 8
....................................AUAGCGUUGUUGUCUGCGGUUCGGCG............................... 26 6
.....................................UAGCGUUGUUGUCUGCGGUUCGGCG............................... 25 5
......................................AGCGUUGUUGUCUGCGGUUCGGCG............................... 24 6
....GCGUUGUUGUCUGCGGUUCGGCG............................... 23 5
....CGCCUCAUACUAAGUCUUUCC............ 21 10
....GCCUCAUACUAAGUCUUUCC............ 20 81
....GCCUCAUACUAAGUCUUUCCCG.......... 22 5
....CCUCAUACUAAGUCUUUC............. 18 10
....CCUCAUACUAAGUCUUUCC............ 19 104
....CCUCAUACUAAGUCUUUCCC........... 20 38
....CCUCAUACUAAGUCUUUCCCG.......... 21 479
....CUCAUACUAAGUCUUUCC............ 18 6
....CUCAUACUAAGUCUUUCCCG.......... 20 91
antisense match

#sja-mir-750
AAACCAGGTGTGTAGAAGTTGGAAGGCCAGATTTAGCGTACTGTCATATTTAAAATCCTTTTAATAACTTGTTCAAAATGAAAAACATACATTTTATGCAATTCTCACAAGAACAAATCATTTCGATTCAGTGAAACAAGTTATTTAACTAGTGAATAGTAGGATTTTACCGCCAGATCTGTCGCTTCCAACTCTATAAATTCTGGCCTAACAAC
Total sRNA: 410991 shared 5' end with miRNA: 99581 shared 3' end with miRNA: 17269
miRNA
....CCAGAUCUGUCGCUUCCAACU........................... 21 281357
Shared 5' Terminus
....CCAGAUCUGUCGCUUCCA.............................. 18 544
....CCAGAUCUGUCGCUUCCAA............................. 19 5141
....CCAGAUCUGUCGCUUCCAAC............................ 20 83067
....CCAGAUCUGUCGCUUCCAACUC.......................... 22 5124
....CCAGAUCUGUCGCUUCCAACUCU......................... 23 5657
....CCAGAUCUGUCGCUUCCAACUCUA........................ 24 42
....CCAGAUCUGUCGCUUCCAACUCUAU....................... 25 5
Shared 3' Terminus
....GAUCUGUCGCUUCCAACU........................... 18 13
....AGAUCUGUCGCUUCCAACU........................... 19 645
....CAGAUCUGUCGCUUCCAACU........................... 20 16594
....GCCAGAUCUGUCGCUUCCAACU........................... 22 11
Subsequence
....CAGAUCUGUCGCUUCCAA............................. 18 134
....CAGAUCUGUCGCUUCCAAC............................ 19 2375
....AGAUCUGUCGCUUCCAAC............................ 18 57
Others
...............AAGUUGGAAGGCCAGAUUU.. 19 27
...............AAGUUGGAAGGCCAGAUUUA... 20 85
...............AAGUUGGAAGGCCAGAUUUAG.. 21 101
...............AAGUUGGAAGGCCAGAUUUAGC... 22 250
...............AAGUUGGAAGGCCAGAUUUAGCG.. 23 7590
...............AAGUUGGAAGGCCAGAUUUAGCGU... 24 260
...............AAGUUGGAAGGCCAGAUUUAGCGUA.. 25 10
................AGUUGGAAGGCCAGAUUUAGCG.. 22 10
....CAGAUCUGUCGCUUCCAACUC.......................... 21 483
....CAGAUCUGUCGCUUCCAACUCU......................... 22 919
....CAGAUCUGUCGCUUCCAACUCUA........................ 23 8
....AGAUCUGUCGCUUCCAACUC.......................... 20 48
....AGAUCUGUCGCUUCCAACUCU......................... 21 399
antisense match

#sja-mir-7b
CCAGAGAAACTTGGAAGACTTGTGATTTAGTTGTTTAATGTCGAAATACAAGTAAAACATTGTCATATCTAAATGAACAACAATCACAAATCTCCATGTGAAATCTGGT
Total sRNA: 5928 shared 5' end with miRNA: 1212 shared 3' end with miRNA: 62
miRNA
..........UGGAAGACUUGUGAUUUAGUUGUU... 24 4493
Shared 5' Terminus
..........UGGAAGACUUGUGAUUUA... 18 12
..........UGGAAGACUUGUGAUUUAG.. 19 108
..........UGGAAGACUUGUGAUUUAGU... 20 110
..........UGGAAGACUUGUGAUUUAGUU.. 21 40
..........UGGAAGACUUGUGAUUUAGUUG... 22 102
..........UGGAAGACUUGUGAUUUAGUUGU.. 23 824
..........UGGAAGACUUGUGAUUUAGUUGUUU.. 25 15
Shared 3' Terminus
............GAAGACUUGUGAUUUAGUUGUU... 22 6
...........GGAAGACUUGUGAUUUAGUUGUU... 23 52
Subsequence
...........GGAAGACUUGUGAUUUAGUUGU.. 22 13
Others
...................................UAAUGUCGAAAUACAAGUAA.. 20 9
...................................UAAUGUCGAAAUACAAGUAAAACAU... 25 7
...................................UAAUGUCGAAAUACAAGUAAAACAUU.. 26 5
....ACAACAAUCACAAAUCUCCAUG........... 22 5
....CAACAAUCACAAAUCUCCAU............ 20 17

```
.............................................................................CAACAAUCACAAAUCUCCAUG...........  21       45
.............................................................................CAACAAUCACAAAUCUCCAUGU..........  22       9
..............................................................................AACAAUCACAAAUCUCCAUG...........  20       24
..............................................................................AACAAUCACAAAUCUCCAUGU..........  21       8
antisense match


#sja-mir-8
ATGAGTATGGCATCTTACTAATAGTATTTGATAAAGAATTTTCTAATACTGTTAGGTAAAGATGCCAGCTCACTTC
Total sRNA: 6350 shared 5' end with miRNA: 2014    shared 3' end with miRNA: 28
miRNA
...........................................UAAUACUGUUAGGUAAAGAUGCC.......... 23       4289
Shared 5' Terminus
...........................................UAAUACUGUUAGGUAAAG............... 18       8
...........................................UAAUACUGUUAGGUAAAGA.............. 19       29
...........................................UAAUACUGUUAGGUAAAGAU............. 20       180
...........................................UAAUACUGUUAGGUAAAGAUG............ 21       138
...........................................UAAUACUGUUAGGUAAAGAUGC........... 22       1005
...........................................UAAUACUGUUAGGUAAAGAUGCCA......... 24       654
Shared 3' Terminus
.............................................AUACUGUUAGGUAAAGAUGCC.......... 21       7
............................................AAUACUGUUAGGUAAAGAUGCC.......... 22       20
Subsequence
............................................AAUACUGUUAGGUAAAGAUGC........... 21       6
Others
antisense match


#sja-mir-new1
AATTATTTCTGATAGAGAGAGCACTTTTATGACGGAGACGAATATTATCTTCGAATTTTATTTTGAAGTTCCATAATGTCATGGAGTTGCTCTCTATATCAGGTTAATTG
Total sRNA: 1527 shared 5' end with miRNA: 73      shared 3' end with miRNA: 4
miRNA
..............GAGAGAGCACUUUUAUGACGGA..........................................................................  22       1192
Shared 5' Terminus
..............GAGAGAGCACUUUUAUGACG............................................................................  20       22
..............GAGAGAGCACUUUUAUGACGG...........................................................................  21       38
..............GAGAGAGCACUUUUAUGACGGAG.........................................................................  23       8
Shared 3' Terminus
Subsequence
Others
............UAGAGAGAGCACUUUUAUGACG............................................................................  22       30
.............AGAGAGAGCACUUUUAUGACG............................................................................  21       187
............................................................................UGUCAUGGAGUUGCUCUCUAU.............  21       9
............................................................................UGUCAUGGAGUUGCUCUCUAUA............  22       12
antisense match


#sja-mir-new2
GTATTGGTTTAGCTAAATAGGTTAGTTTGACTGTCTGTTTTCAAAATCGGGAGGTGGTTACGTATGATGTAGTCTTTAGTTGGATTTAGG
Total sRNA: 21014shared 5' end with miRNA: 3253    shared 3' end with miRNA: 1329
miRNA
..........AGCUAAAUAGGUUAGUUUGACUGUC.......................................................    25       15436
Shared 5' Terminus
..........AGCUAAAUAGGUUAGUUUG.............................................................    19       28
..........AGCUAAAUAGGUUAGUUUGA............................................................    20       70
..........AGCUAAAUAGGUUAGUUUGAC...........................................................    21       33
..........AGCUAAAUAGGUUAGUUUGACU..........................................................    22       103
..........AGCUAAAUAGGUUAGUUUGACUG.........................................................    23       1318
..........AGCUAAAUAGGUUAGUUUGACUGU........................................................    24       1359
..........AGCUAAAUAGGUUAGUUUGACUGUCU......................................................    26       339
Shared 3' Terminus
.................UAGGUUAGUUUGACUGUC.......................................................    18       342
................AUAGGUUAGUUUGACUGUC.......................................................    19       66
...............AAUAGGUUAGUUUGACUGUC.......................................................    20       31
..............AAAUAGGUUAGUUUGACUGUC.......................................................    21       379
.............UAAAUAGGUUAGUUUGACUGUC.......................................................    22       14
............CUAAAUAGGUUAGUUUGACUGUC.......................................................    23       6
...........GCUAAAUAGGUUAGUUUGACUGUC.......................................................    24       491
Subsequence
```

```
...........GCUAAAUAGGUUAGUUUGACUG.........................................................    22    29
...........GCUAAAUAGGUUAGUUUGACUGU........................................................    23    36
..............AAAUAGGUUAGUUUGACU..........................................................    18    6
..............AAAUAGGUUAGUUUGACUG.........................................................    19    15
..............AAAUAGGUUAGUUUGACUGU........................................................    20    7
................AUAGGUUAGUUUGACUGU........................................................    18    5
Others
..............AAAUAGGUUAGUUUGACUGUCU......................................................    22    7
..............AAAUAGGUUAGUUUGACUGUCUGUUU..................................................    26    5
................AUAGGUUAGUUUGACUGUCU......................................................    20    5
................AUAGGUUAGUUUGACUGUCUGUU...................................................    23    7
................AUAGGUUAGUUUGACUGUCUGUUU..................................................    24    7
...............................................CGGGAGGUGGUUACGUAUG........................    19    11
...............................................CGGGAGGUGGUUACGUAUGAUGUA...................    24    14
................................................GGGAGGUGGUUACGUAUGAU......................    20    13
...................................................AGGUGGUUACGUAUGAUGUA...................    20    9
.......................................................GGUUACGUAUGAUGUAGU.................    18    131
.......................................................GGUUACGUAUGAUGUAGUC................    19    53
.......................................................GGUUACGUAUGAUGUAGUCU...............    20    285
.......................................................GGUUACGUAUGAUGUAGUCUU..............    21    111
.......................................................GGUUACGUAUGAUGUAGUCUUU.............    22    43
.......................................................GGUUACGUAUGAUGUAGUCUUUA............    23    34
.......................................................GGUUACGUAUGAUGUAGUCUUUAG...........    24    7
.......................................................GGUUACGUAUGAUGUAGUCUUUAGUUG........    27    16
...............................................................AUGAUGUAGUCUUUAGUU.........    18    5
antisense match
```
