# Supplementary material for: MicroRNAs Are Involved in the Regulation of Ovary Development in the Pathogenic Blood Fluke Schistosoma japonicum
Source: PLoS Pathog. 2016 Feb 12;12(2):e1005423. doi: 10.1371/journal.ppat.1005423 (PMC4752461; doi:10.1371/journal.ppat.1005423)
Supplement: S1 Table — (PDF) [file ppat.1005423.s014.pdf]

S1 Table. Schistosome small RNA sequences

	16F	16M	22F	22M	28F	28M	F	M
Total reads	14,338,843	16,011,457	14,639,621	13,394,192	14,884,947	14,280,870	14,945,055	14,363,457
High quality	14,277,073 (100%)	15,944,619 (100%)	14,579,258 (100%)	13,337,718 (100%)	14,820,290 (100%)	14,221,100 (100%)	12,531,556 (100%)	12,194,511 (100%)
3' adapter null	2,838 (0.02%)	2,710 (0.02%)	3,287 (0.02%)	3,239 (0.02%)	3,762 (0.03%)	2,674 (0.02%)	2,368 (0.02%)	2,133 (0.02%)
Insert null	1,044 (0.01%)	1,003 (0.01%)	1,157 (0.01%)	1,012 (0.01%)	2,144 (0.01%)	1,570 (0.01%)	16,583 (0.13%)	13,445 (0.11%)
5'adapter contaminants	26,684 (0.19%)	23,385 (0.15%)	33,290 (0.23%)	29,933 (0.22%)	39,343 (0.27%)	36,595 (0.26%)	173,180 (1.38%)	99,028 (0.81%)
Smaller than 18nt	93,928 (0.66%)	192,886 (1.21%)	158,346 (1.09%)	58,546 (0.44%)	725,630 (4.9%)	301,643 (2.12%)	215,632 (1.72%)	89,823 (0.74%)
PolyA	283 (0.00%)	671 (0.00)	673 (0.00%)	856 (0.01%)	357 (0.00%)	600 (0.00%)	484 (0.00%)	535 (0.00%)
Clean reads	14,152,296 (99.13%)	15,723,964 (98.62%)	14,382,505 (98.65%)	13,244,132 (99.30%	14,049,054 (94.8%)	13,878,018 (97.59%)	12,123,309 (96.74%)	11,989,547 (98.32%)
Sequences matched to the genome	7,964,991 (56.28%)	8,947,298 (56.90%)	6,693,114 (46.54%)	6,541,610 (46.54%)	8,895,031 (63.31%)	7,043,390 (50.75%)	6,715,016 (55.39%)	6,303,431 (52.57%)
