# Supplementary material for: MicroRNAs Are Involved in the Regulation of Ovary Development in the Pathogenic Blood Fluke Schistosoma japonicum
Source: PLoS Pathog. 2016 Feb 12;12(2):e1005423. doi: 10.1371/journal.ppat.1005423 (PMC4752461; doi:10.1371/journal.ppat.1005423)
Supplement: S3 Table — (PDF) [file ppat.1005423.s016.pdf]

S3 Table. Oligonucleotides used as probes for Northern blots

Names	Sequences
sja-let-7b	5`/DigN/AGTCATATGAATCACTACCTCT3`
sja-let-7s*	5`/DigN/AGTCGTACATCTAACTACCTC3`
sja-miR-750	5`/DigN/AGTTGGAAGCGACAGATCTGG3`
sja-miR-1175	5`/DigN/CAGTTGAAGTAATTGAATCTCA3`
sja-miR-1989	5`/DigN/TCGAAGACATGAACACAGCTGA3`
sja-miR-new1	5`/DigN/TCCGTCATAAAAGTGCTCTCTC3`
sja-miR-new2	5`/DigN/GACAGTCAAACTAACCTATTTAGCT3`
sja-lin-4	5`/DigN/ACAACTCTAAGGTCTCAGGGA3`
sja-miR-1b	5`/DigN/GCACATACTTCACAACATTCCA3`
sja-miR-277b	5`/DigN/TCTAGGGTAGATGATGCATTTT3`
