# Supplementary material for: MicroRNAs Are Involved in the Regulation of Ovary Development in the Pathogenic Blood Fluke Schistosoma japonicum
Source: PLoS Pathog. 2016 Feb 12;12(2):e1005423. doi: 10.1371/journal.ppat.1005423 (PMC4752461; doi:10.1371/journal.ppat.1005423)
Supplement: S4 Table — (PDF) [file ppat.1005423.s017.pdf]

S4 Table. Primers for stem-loop RT-PCR analyses

Names	Sequences	
sja-miR-277b	5`GTCGTATCCAGTGCAGGGTCCGAGGTATTCGCACTGGATACGAC*TCTAGG3*`	Reverse transcript
sja-miR-1175	5`GTCGTATCCAGTGCAGGGTCCGAGGTATTCGCACTGGATACGAC*CAGTTG3*`	Reverse transcript
sja-miR-1989	5`GTCGTATCCAGTGCAGGGTCCGAGGTATTCGCACTGGATACGAC*TCGAAG3*`	Reverse transcript
sja-lin-4	5`GTCGTATCCAGTGCAGGGTCCGAGGTATTCGCACTGGATACGAC*ACAACT3*`	Reverse transcript
sja-miR-new1	5`GTCGTATCCAGTGCAGGGTCCGAGGTATTCGCACTGGATACGAC*TCCGTC3*`	Reverse transcript
sja-miR-new2	5`GTCGTATCCAGTGCAGGGTCCGAGGTATTCGCACTGGATACGACGACAGT3`	Reverse transcript
sja-let-7	5`GTCGTATCCAGTGCAGGGTCCGAGGTATTCGCACTGGATACGAC*ACCACA3*`	Reverse transcript
sja-let-7b	5`GTCGTATCCAGTGCAGGGTCCGAGGTATTCGCACTGGATACGAC*AGTCAT3*`	Reverse transcript
sja-let-7s*	5`GTCGTATCCAGTGCAGGGTCCGAGGTATTCGCACTGGATACGAC*AGTCGT3*`	Reverse transcript
sja-mir-1	5`GTCGTATCCAGTGCAGGGTCCGAGGTATTCGCACTGGATACGAC*GACCAT3*`	Reverse transcript
sja-mir-2a	5`GTCGTATCCAGTGCAGGGTCCGAGGTATTCGCACTGGATACGAC*CGTTCA3*`	Reverse transcript
sja-mir-7b	5`GTCGTATCCAGTGCAGGGTCCGAGGTATTCGCACTGGATACGAC*AACAAC3*`	Reverse transcript
sja-mir-10	5`GTCGTATCCAGTGCAGGGTCCGAGGTATTCGCACTGGATACGAC*CCAAAC3*`	Reverse transcript
sja-mir-36	5`GTCGTATCCAGTGCAGGGTCCGAGGTATTCGCACTGGATACGAC*GCGAAT3*`	Reverse transcript
sja-mir-71a	5`GTCGTATCCAGTGCAGGGTCCGAGGTATTCGCACTGGATACGAC*CATCTC3*`	Reverse transcript
sja-mir-71b	5`GTCGTATCCAGTGCAGGGTCCGAGGTATTCGCACTGGATACGAC*CGTCTC3*`	Reverse transcript
sja-mir-125a	5`GTCGTATCCAGTGCAGGGTCCGAGGTATTCGCACTGGATACGAC*GACAATC3*`	Reverse transcript
sja-mir-125b	5`GTCGTATCCAGTGCAGGGTCCGAGGTATTCGCACTGGATACGAC*AGCAAT3*`	Reverse transcript
sja-mir-190	5`GTCGTATCCAGTGCAGGGTCCGAGGTATTCGCACTGGATACGAC*ACCAAG3*`	Reverse transcript
sja-mir-307	5`GTCGTATCCAGTGCAGGGTCCGAGGTATTCGCACTGGATACGAC*CATCAA3*`	Reverse transcript
sja-mir-750	5`GTCGTATCCAGTGCAGGGTCCGAGGTATTCGCACTGGATACGAC*AGTTGG3*`	Reverse

		transcript
sja-mir-1b	5`GTCGTATCCAGTGCAGGGTCCGAGGTATTCGCACTGGATACGAC*GCACAT3*`	Reverse transcript
sja-mir-3505	5`GTCGTATCCAGTGCAGGGTCCGAGGTATTCGCACTGGATACGAC*TTTCTA3*`	Reverse transcript
sja-miR-277b_F	5`ATCGTACGTGGG*AAAATGCAT3*`	PCR
sja-miR-1175_F	5`ATCGTACGTGGG*TGAGATTCA3*`	PCR
sja-miR-1989_F	5`ATCGTACGTGGG*TCAGCTGTG3*`	PCR
sja-lin-4_F	5`ATCGTACGTGGG*TCCCTGAGA3*`	PCR
sja-miR-new1_F	5`ATCGTACGTGGG*GAGAGAGCA3*`	PCR
sja-miR-new2_F	5`ATCGTACGTGGGAGCTAAAT3`	PCR
sja-let-7_F	5`ATCGTACGTGGG*GGAGGTAGT3*`	PCR
sja-let-7b_F	5`ATCGTACGTGGG*AGAGGTAG3*`	PCR
sja-let-7s*_F	5`ATCGTACGTGGG*GAGGTAGTT3*`	PCR
sja-mir-1_F	5`ATCGTACGTGGG*TGGAATGTG3*`	PCR
sja-mir-2a_F	5`ATCGTACGTGGG*TCACAGCCA3*`	PCR
sja-mir-7b_F	5`ATCGTACGTGGG*TGGAAGACT3*`	PCR
sja-mir-10_F	5`ATCGTACGTGGG*AACCCTGTA3*`	PCR
sja-mir-36_F	5`ATCGTACGTGGG*CCACCGGGT3*`	PCR
sja-mir-71a_F	5`ATCGTACGTGGG*TGAAAGACG3*`	PCR
sja-mir-71b_F	5`ATCGTACGTGGG*TGAAAGACT3*`	PCR
sja-mir-125a_F	5`ATCGTACGTGGG*TCCCTGAGACCCTTT3*`	PCR
sja-mir-125b_F	5`ATCGTACGTGGG*TCCCTGAGACTGA3*`	PCR
sja-mir-190_F	5`ATCGTACGTGGG*TGATATGTA3*`	PCR
sja-mir-307_F	5`ATCGTACGTGGG*CCTCATAAA3*`	PCR
sja-mir-750_F	5`ATCGTACGTGGG*CCAGATCTG3*`	PCR
sja-mir-1b_F	5`ATCGTACGTGGG*TGGAATGTT3*`	PCR
sja-mir-3505_F	5`ATCGTACGTGGG*TGACTGTC3*`	PCR
Universal primer	5` GCAGGGTCCGAGGTATTC3`	PCR
