# Supplementary material for: MicroRNAs Are Involved in the Regulation of Ovary Development in the Pathogenic Blood Fluke Schistosoma japonicum
Source: PLoS Pathog. 2016 Feb 12;12(2):e1005423. doi: 10.1371/journal.ppat.1005423 (PMC4752461; doi:10.1371/journal.ppat.1005423)
Supplement: S5 Table — (PDF) [file ppat.1005423.s018.pdf]

S5 Table. Primers used for miRNA target identification

Primer names	Sequences
let-7	5`CCACACAACGAACTACCTC3`
let-7b	5`GTCATATGAATCACTACCTC3`
let-7s	5`AGTCGTACATCTAACTACCT3`
miR-1	5`ACCATACTTCGCCACATTCC3`
miR-1b	5`CACATACTTCACAACATTCC3`
miR-2d	5`GTCACCTAAGCAGGACTGTGAT3`
miR-2b	5`GTGTCCCAAGCAGGGCTGTGAT3`
miR-2a	5`GTTCATCAATACTGGCTGTG3`
miR-7	5`ACAACATATCACCAGTCTTCC3`
miR-7b	5`ACAACTAAATCACAAGTCTTCC3`
miR-8	5`GCATCTTTACCTAACAGTATT3`
miR-10	5`CAAACTCGGGTCTACAGGGT3`
miR-31	5`CAGCTTCGCCGTAATCTTGCC3`
miR-36	5`CGAATGAATGTCTACCCGGTG3`
miR-2162	5`GAGTGAAACGTTGCATAAT3`
miR-61	5`AAGTGAGTGCACTTTCTAGTC3`
miR-71a	5`ATCTCACTACCATCGTCTTTC3`
miR-71b	5`GTCTCACTACTCAAGTCTTTC3`
miR-124	5`GACATTCACCGCGTGCCTT3`
miR-125a	5`CAATCAAAGGGTCTCAGGG3`
miR-125b	5`GCAATTATCAGTCTCAGGG3`
miR-190	5`CCAAGTAACCCATACATATC3`
miR-219	5`AGAAATGCGAATGGACAATC3`
miR-3479	5`AAGGCGAAGGTAAGTGCAAT3`
miR-277	5`ACGGGCCAGAAAATGCATTT3`
miR-277b	5`CTAGGGTAGATGATGCATTT3`
miR-750	5`GAGTTGGAAGCGACAGATCTG3`
miR-1175	5`AGTTGAAGTAATTGAATCTC3`
miR-2c	5`ACATCACAGTCGAACAAGGG3`
miR-2e	5`TAACTCAGTCTAAGTTGGT3`
Universal down primer	5`GCTGTCAACGATACGCTACGTAACG3`
Bantam	5`CCAGCTTTAATCGCGATCTC3`
miR-1989	5`CGAAGACATGAACACAGCTG3`
miR-new1	5`CCGTCATAAAAGTGCTCTCT3`
lin-4	5`CAACTCTAAGGTCTCAGGG3`
