# Supplementary material for: MicroRNAs Are Involved in the Regulation of Ovary Development in the Pathogenic Blood Fluke Schistosoma japonicum
Source: PLoS Pathog. 2016 Feb 12;12(2):e1005423. doi: 10.1371/journal.ppat.1005423 (PMC4752461; doi:10.1371/journal.ppat.1005423)
Supplement: S6 Table — (PDF) [file ppat.1005423.s019.pdf]

S6 Table. List of the potential target genes for *S. japonicum* miRNAs

miRNAs	Target gene IDs	Name of target genes	MiRNA:mRNA duplex*	Mfe (kcal/mol)
miR-new1	AY812714.1	Outer dense fiber of sperm tails 2	target 5' A UUUCUUGU A 3'<br>UUGUC AGUGCUCUCU<br>GGCAG UCACGAGAGA<br>miRNA 3' A UAUUU G 5'	-22.6
	AY811515.1	T-complex	target 5' G A UUA G 3'<br>CUGUC AUGAA GCUCU<br>GGCAG UAUUU CGAGA<br>miRNA 3' A UCA GAG 5'	-21.1
Let-7	FN315235.1	Hypothetical protein	target 5' UG U UUGUU G 3'<br>U A CAAC ACUACCUC<br>G U GUUG UGAUGGAG<br>miRNA 3' UG UG CU G 5'	-21.1
	FN327005.1	Hypothetical protein	target 5' A UG U A G 3'<br>U A CAAC GAACUACCUC<br>G U GUUG CUUGAUGGAG<br>miRNA 3' UG UG G 5'	-26.4
	AY814216.1	Hypothetical protein	target 5' A UG U A G 3'<br>U A CAAC GAACUACCUC<br>G U GUUG CUUGAUGGAG<br>miRNA 3' UG UG G 5'	-26.4
Let-7b	AY810070.1	Lim domain	target 5' U G 3'<br>GUAUGAAU CAUUACCUC<br>UAUACUUA GUGAUGGAG<br>miRNA 3' UCAG A 5'	-25.4
	AY814216.1	Hypothetical protein	target 5' A GAUCAACAGA G 3'<br>UAUGUGAAUU ACUACCUC<br>GUAUACUUAG UGAUGGAG<br>miRNA 3' UCA A 5'	-26.0
Let-7s	FN317371.1	Hypothetical protein	target 5' G UUUAUCUUUC U G 3'<br>UGUA CAUCUA CUACCU<br>GCAU GUAGAU GAUGGA<br>miRNA 3' UCA U G 5'	-21.0
miR-2a	AY811143.1	CDS of Lipid synthesized	target 5' A AG AAU C 3'<br>GU AU CUGGCUGUG<br>CA UA GACCGACAC<br>miRNA 3' G AG GUUAU U 5'	-22.8
	AY336493.1	RNA polymerase B transcription factor 3	target 5' U CCUU U A C 3'<br>CGUUC UUA UACUG CUGUG<br>GCAAG AGU AUGAC GACAC<br>miRNA 3' U U C U 5'	-22.5
	AY223360.1	Hypothetical protein	target 5' A UUU AU C 3'<br>UGUUCGUCA CUG CUGUGG<br>GCAAGUAGU GAC GACACU<br>miRNA 3' UAU C 5'	-25.4
miR-1	FN318139.1	Putative signal peptide peptidase-like 2A	target 5' A 3'<br>CAUA CCACAUUCC<br>GUAU GGUGUAAGG<br>miRNA 3' CUG GAAGC U 5'	-19.7
	AY915410.1	PKC	target 5' C C C 3'<br>ACCA UCGCCAC UCC<br>UGGU AGCGGUG AGG<br>miRNA 3' C AUGA UA U 5'	-23.4
Lin-4	FN324931.1	Ploy A binding protein	target 5' A C A C U 3'<br>AC ACUU AA GUCUCA GG<br>UG UGAG UU CAGAGU CC<br>miRNA 3' U A C C U 5'	-19.0

miR-2e	AY815588.1	Poly A binding protein	target 5' A AAACUUACAGGAAU UCU UG A 3'<br>GCCAGA GCU UGG UG GAUA<br>UGGUUU CGA ACC AC CUAU<br>miRNA 3' UG A 5'	-23.6
miR-219	AY816105.1	Hypothetical protein	target 5' A ACAGC ACAA C 3'<br>GAGAAAUGC UGGA AAUC<br>UUCUUUACG ACCU UUAG<br>miRNA 3' CUU G U 5'	-22.0
miR-124	AY815898.1	26S proteasome regulatory subunit S3	target 5' U UG A UA C 3'<br>UG A UU GCGUGCCUU<br>AC U AG CGCACGGAA<br>miRNA 3' UG A UGG U 5'	-24.5
	AY815673.1	COP9 signalosome complex	target 5' U UG A UA C 3'<br>UG A UU GCGUGCCUU<br>AC U AG CGCACGGAA<br>miRNA 3' UG A UGG U 5'	-24.5
miR-277	AY815194.1#	SJCHGC05742 protein	target 5' G UUACAAUAC A 3'<br>GGGUCG GUAUUUA<br>CCCGGU CGUAAAU<br>miRNA 3' UG CUUUUA 5'	-19.0

* miRNA:mRNA pair analysis was performed using RNAhybrid (http://bibiserv.techfak.uni-bielefeld.de/rnahybrid/)

# indicates that the target mRNA was not successfully verified by luciferase assay.
