# Supplementary material for: MicroRNAs Are Involved in the Regulation of Ovary Development in the Pathogenic Blood Fluke Schistosoma japonicum
Source: PLoS Pathog. 2016 Feb 12;12(2):e1005423. doi: 10.1371/journal.ppat.1005423 (PMC4752461; doi:10.1371/journal.ppat.1005423)
Supplement: S7 Table — (PDF) [file ppat.1005423.s020.pdf]

S7 Table. Primer pairs used to generate miRNA target regions for pGLU-CMV luciferase vector constructs

GenBank IDs	miRNAs	Sequences	Enzyme sites
FN321618.1	miR-2	ATAAGAATGCGGCCGCTTGTTGAAGAAAGGCAAAC	*Not* Ⅰ
		CCGCTCGAGCAATCGTGGAACTGTGAC	*Xho* Ⅰ
FN314191.1	let-7a	ATAAGAATGCGGCCGCCAGTGGATACTACTTCGGAT	*Not* Ⅰ
		CCGCTCGAGTTAAACAGCATAGATTTGG	*Xho* Ⅰ
DQ643829	miR-8	GCCTCGAGAAGAAAGATTTCATGAAG	*Xho* Ⅰ
		CCTCTAGAATATCTTGGATCATGATGAC	*Xba* Ⅰ
FN313640	miR-8	GCCTCGAGCAATCTCTCAAGCAACGAAC	*Xho* Ⅰ
		CCTCTAGAACACCGGAATATGTAGTAAA	*Xba* Ⅰ
EU370927	miR-31	CCGGCGGCCGCCAGCATTAGAGAGACCTGAG	*Not* Ⅰ
		CGCTCGAGAACAAGGGAACAGAACCAGG	*Xho* Ⅰ
FN317226	miR-1989	GCCTCGAGCACTATTTAATCGCACCAAC	*Xho* Ⅰ
		CCTCTAGATAAGTGTTATTTGTATCAGG	*Xba* Ⅰ
FJ753578.1	miR-3479	GCCTCGAGCAGTTGTGTTGTACTTACAG	*Xho* Ⅰ
		CCTCTAGACATGCTTCACATTGTGCGTGCT	*Xba* Ⅰ
FN323394.1	Bantam	GCCTCGAGCACGCAGCGACCGTAGAGAA	*Xho* Ⅰ
		CCTCTAGAAAAAAACCGGAACGAGATCGTGA	*Xba* Ⅰ
AY815194.1	miR-277	ATAAGAATGCGGCCGCAGAAAAACAATTTCTTTACTTC	*Not* Ⅰ
		CCGCTCGAGAGATGAAGATTACATATAT	*Xho* Ⅰ
