# Supplementary material for: MicroRNAs Are Involved in the Regulation of Ovary Development in the Pathogenic Blood Fluke Schistosoma japonicum
Source: PLoS Pathog. 2016 Feb 12;12(2):e1005423. doi: 10.1371/journal.ppat.1005423 (PMC4752461; doi:10.1371/journal.ppat.1005423)
Supplement: S8 Table — (PDF) [file ppat.1005423.s021.pdf]

**S8 Table. miRNA mimics used for cell transfection and worm electroporation**

| Names | Sequences | Modifications |
|---|---|---|
| let-7a | GGAGGUAGUUCGUUGUGUGGU | 2`-Ome |
| Antisense-let-7a | ACCACACAACGAACUACCUCC | 2`-Ome |
| Scrambled miRNA | CCUCCAUCAACGUUGUGUGGU | 2`-Ome |
| | | |
| miR-2 | UCACAGCCAGUAUUGAUGAACG | 2`-Ome |
| Antisense-miR-2 | CGUUCAUCAAUACUGGCUGUGA | 2`-Ome |
| Scrambled miRNA | GGUGCAGAGCUAUUGAUGAACG | 2`-Ome |
| | | |
| miR-8 mimics | UAAUACUGUUAGGUAAAGAUGCC | 2`-Ome,PS |
| Antisense-miR-8 | GGCAUCUUUACCUAACAGUAUUA | 2`-Ome,PS |
| Scrambled miR-8 | GUAUUGCAAGACAUAAAGAUGCC | 2`-Ome,PS |
| | | |
| miR-31 mimics | UGGCAAGAUUACGGCGAAGCU | 2`-Ome,PS |
| Antisense-miR-31 | AGCUUCGCCGUAAUCUUGCCA | 2`-Ome,PS |
| Scrambled miR-31 | GCGCAUACGGUCGGCGAAGCU | 2`-Ome,PS |
| | | |
| miR-3479 mimics | UAUUGCACUUACCUUCGCCUUG | 2`-Ome,PS |
| Antisense-miR-3479 | CAAGGCGAAGGUAAGUGCAAUA | 2`-Ome,PS |
| Scrambled miR-3479 | GUAACGUGAAACCAUCGCGUUG | 2`-Ome,PS |
| | | |
| Bantam mimics | UGAGAUCGCGAUUAAAGCU | 2`-Ome,PS |
| Antisense-bantam | AGCUUUAAUCGCGAUCUCA | 2`-Ome,PS |
| Scrambled bantam | GCUCUGGCGGAUUAAAGCU | 2`-Ome,PS |
| | | |
| miR-277 mimics | UAAAUGCAUUUUCUGGCCCGU | 2`-Ome,PS |
| Antisense-miR-277 | ACGGGCCAGAAAAUGCAUUUA | 2`-Ome,PS |
| Scrambled miR-277 | UCUGCCGACCAUCUGGCCCGU | 2`-Ome,PS |
