# Supplementary material for: MicroRNAs Are Involved in the Regulation of Ovary Development in the Pathogenic Blood Fluke Schistosoma japonicum
Source: PLoS Pathog. 2016 Feb 12;12(2):e1005423. doi: 10.1371/journal.ppat.1005423 (PMC4752461; doi:10.1371/journal.ppat.1005423)
Supplement: S9 Table — (PDF) [file ppat.1005423.s022.pdf]

S9 Table. Primers used for miRNA suppression studies on *in vivo* cultured schistosomes

miRNAs	GenBank IDs	Primer sequences
Bantam	AY223092.1	5`ACGCGACGAACCCCATCGTG3`
		5` ACCGCGATCTGGTGGGGGAT3`
Bantam	FN323394.1	5` ACGCGACGAACCCCATCGTG3`
		5` GACCGCGATCTGGTGGGGGA3`
Bantam	AY815078.1	5` TTGGCGGTGAGGTGTATG3`
		5` TGAACAACCTGGTGGAAT3`
miR-31	FN319623.1	5` CGCGCAGCGACTTTGCTTGG3`
		5` CGCGAAGGGCGGAAGGAAGG3`
