# Supplementary material for: MicroRNAs Are Involved in the Regulation of Ovary Development in the Pathogenic Blood Fluke Schistosoma japonicum
Source: PLoS Pathog. 2016 Feb 12;12(2):e1005423. doi: 10.1371/journal.ppat.1005423 (PMC4752461; doi:10.1371/journal.ppat.1005423)
Supplement: S10 Table — (PDF) [file ppat.1005423.s023.pdf]

S10 Table. LNAs used for miRNA in situ hybridization and primers used for probe preparation

miR-31	/5DigN/AGCTTCGCCGTAATCTTGCCA/3Dig_N/	size
Bantam	/5DigN/ACCAGCTTTAATCGCGATCTCA/3Dig_N/	
Scrambled Sj miRNA	/5DigN/GTGTAACACGTCTATACGCCCA/3Dig_N/	
Frizz7<br>(EU370927)	Forward primer: 5’TGTTGGCGAAGCATTCAACG3`<br>Reverse primer: 5`<br>ATCGAAATTAATACGACTCACTATAGGGCCGACTGAGAAGGTGAGCGAA3`	661nt
Smad1<br>(AY815078.1)	Forward primer: 5`TGGGGCATGCAACTCAAGAT3`<br>Reverse primer: 5` ATCGAAATTAATACGACTCACTATAGGGC<br>CTGAGAGACTGTGGTGCTGG3`	336nt
