# Supplementary material for: MicroRNAs Are Involved in the Regulation of Ovary Development in the Pathogenic Blood Fluke Schistosoma japonicum
Source: PLoS Pathog. 2016 Feb 12;12(2):e1005423. doi: 10.1371/journal.ppat.1005423 (PMC4752461; doi:10.1371/journal.ppat.1005423)
Supplement: S11 Table — (PDF) [file ppat.1005423.s024.pdf]

S11 Table. Putative miRNAs identified in the sequencing that are not supported by our additional miRNA criteria

miRNA names	Reasons	Sequences	Read #
sja-mir-3489	across & both strands	GCCACAACAGUUCGAGGACG	3,909
sja-mir-3490	across & both strands	UCCUUUGGGUUGUGGGGAUA	1,041
sja-mir-3491	across & both strands	UGAGCGAUUACUGGAGUUCA	936
sja-mir-3492	across & both strands	AUCCGUGCUGAGAUUUCGUCU	2,455
sja-mir-3493	across & both strands	UGGAUUUCAGUAGCAUCCACU	1,826
sja-mir-3494	across & both strands	GACAGAAGACUGCCGCUCAU	667
sja-mir-3495	across & both strands	UCAAAAUCGUUGGCAAUGGCU	1,397
sja-mir-3496	across & both strands	CGGCGUCUGGACGUUUGGUUUU	811
sja-mir-3497	across & both strands	CGCAAGGGACUACCAUGGCA	389
sja-mir-3498	across & both strands	UAGACGGUUUGAAACAUGGA	957
sja-mir-3499	across & both strands	UCUGAAUCCGUGCUGAGAUUUU	729
sja-mir-3500	across & both strands	AGGAGAUCGGUGGUAGAUUGU	612
sja-mir-3501	across & both strands	AACCUUGUAGUUUCGUUGUG	462
sja-mir-3502	across & both strands	GUGACGAUCGUACAUGUCUU	1,378
sja-mir-310	across & both strands & low reads #	UCAAGAUGACGCGACUCUCG	726
sja-mir-3480	across & both strands & low reads #	AAACAGACAUACCAAUGCAG	58
sja-mir-3481	across & both strands & low reads #	GGUGAUCUUUGUAUGGACAA	101
sja-mir-3483	across & both strands & low reads #	UAUGCCUGACCACCGUCUACU	25
sja-mir-3484	across & both strands & low reads #	UUAGGUUUCGUUGUUUGUAUUU	6
sja-mir-3485	across & both strands & low reads #	ACUUGUGGUGUAGGCGAGAC	13
sja-mir-3486	across & both strands & low reads #	UCUGUGUUGAAUUUGAGGAU	122
sja-mir-3504	across & both strands & low reads #	GUGUGGUUGUCAGAAGGGGC	96
sja-mir-3506	across & both strands & low reads #	ACGAGGUUAGACGACGAGUG	65
sja-mir-3507	across & both strands & low reads #	AGAGAAUAGGGCAUGGGUACU	555
sja-mir-3482	very low reads #	GAUGCGCACUGCCGAGGAUUUC	47
sja-mir-133	conserved but not hairpin & low reads #	UUGGUCCCUAUCAACCAGCUGU	151
sja-mir-3503	no bulge hairpin & both strands & low reads #	AGCGGAAUCCAGGACACGCGUUU	172
