# Supplementary material for: MicroRNAs Are Involved in the Regulation of Ovary Development in the Pathogenic Blood Fluke Schistosoma japonicum
Source: PLoS Pathog. 2016 Feb 12;12(2):e1005423. doi: 10.1371/journal.ppat.1005423 (PMC4752461; doi:10.1371/journal.ppat.1005423)
Supplement: S1 File — (PDF) [file ppat.1005423.s025.pdf]

Supplemental Materials and methods

Small RNA libraries, sequencing, and bioinformatics

Small RNA libraries were constructed as described previously [1]. Briefly, RNAs between 15 and 30 nucleotides (nt) in length were excised from 15% TBE urea polyacrylamide gels (PAGE). The library was prepared using the Illumina Small RNA Library Kit and DNA sequencing was carried out using Illumina Genome Analyzer (Hseq2000) at the Beijing Genomics Institute (BGI, Shenzhen, China).

Raw sequencing data were processed as follows: clean reads were obtained after removing the low quality reads and adapter sequences. These reads were collapsed into a non-redundant dataset and mapped onto the *S. japonicum* genome of SGST (http://lifecenter.sgst.cn) using bowtie [2]. Reads that mapped to rRNA, tRNA, snRNA (small nuclear RNA), and snoRNA (small nucleolar RNA) were filtered out before miRNA identification.

miRNAs were identified as described previously [3]. Briefly, genomic regions with small RNA read clusters were extracted and manually inspected for miRNA hairpins. Due to some very large miRNA hairpins in this species (see S13 Fig and S2 Table), the cutoff for the length of the hairpin sequences was extended to 500 bp. We identified 35 miRNA hairpin sequences and their mature miRNA sequences (S13 Fig). In addition, 3 miRNAs (lin-4, miR-1b, and miR-277b) without genomic hairpin sequences but with high expression and sequence conservation in Metazoa were also identified. Thus, a total of 38 high-confidence miRNAs were identified (S13 Fig and S2 Table). Overall, we identified 10 new schistosome miRNAs that are not described in the miRBase and in previous studies [4-6], including 3 miRNAs (let-7b, let-7s, and miR-750) with large (~200 nt) hairpin sequences (S13 Fig).

We found that a small number of siRNAs (not defined by our criteria as miRNAs) reads in our data were mapped to previously described *S. japonicum* miRNAs (v19). On further examination of where these small RNA reads mapped onto predicted *S. japonicum* hairpin sequences, we found that these sequences do not map onto the hairpins in a manner typical of most miRNAs. Many of these hairpins do not represent a true miRNA hairpin after looking at the distribution of the small RNA reads across the whole hairpin. These ambiguous miRNAs from miRBase usually have 2 or more of the following features: (1). Reads mapped across the whole hairpin sequence; (2). Reads mapped to both sense and antisense of the hairpin sequence; (3). Very low number of reads mapped and there's no dominant species; and/or (4). Precursor sequence doesn't form a miRNA hairpin structure. Of the 55 miRNAs from miRBase (v19), 28 were confirmed in our study and the other 27 are likely not miRNAs but perhaps other small RNAs (siRNAs) (S11 Table).

To compare the expression levels, we normalized the miRNA reads to reads per million genome match reads (RPM) based on the sequencing depth of each library. The normalized miRNAs were grouped by using Cluster 3.0 [7] (http://bonsai.ims.u-tokyo.ac.jp/~mdehoon/software/cluster/software.htm) after log2 transforming the fold change value to the average expression across developmental stages, followed by manual adjustment. The heatmap was illustrated by using TreeView program (http://jtreeview.sourceforge.net/) (Fig. 3A).

Real time qRT-PCR analyses of miRNA expression in different stages/sexes/regions of schistosomes

Schistosomes were collected from the rabbits infected with *S. japonicum* at days 16, 22, and 28 post-infection. Total RNA from different stages and sexes of schistosomes was isolated using Trizol (Invitrogen) following the manufacturer's instruction. Then, real-time qRT-PCR was performed as described previously [8] for determining the expression of sex enriched miRNAs. Briefly, total RNA was added to a reverse transcription reaction containing miScript Reverse Transcriptase Mix and miScript Nucleics Mix (Qiagen) incubated at 37℃ for 60 min and 94℃

for 5 min. Real-time PCR was performed by using miScript SYBR Green PCR Kit (Qiagen). Each PCR reaction contained 1 µl diluted cDNA (1:10 dilution) in a final volume of 20 µl containing 10 µl 2× QuantiTect SYBR Green PCR Master Mix, 2 µl 10x miScript Universal Primer and 2 µl miScript miRNA primers and 5µl H2O. qPCR was performed in Mastercycler ep realplex (Eppendorf) using the following thermal cycling profile: 95 ℃ for 15 min, followed by 45 cycles of amplification (94 ℃ for 15 s, 57 ℃ for 30 s, and 70 ℃ for 30 s). The miScript Primers for each miRNA was ordered from Qiagen (Qiagen's proprietary).The expression of *S. japonicum* nicotinamide adenine dinucleotide dehydrogenase (*NADH*) (forward primer: CGA GGA CCT AAC AGC AGA GG; reverse primer: TCC GAA CGA ACT TTG AAT CC) was used as an internal control for normalization.

Stem loop based qRT-PCR was performed for determining the expression of miRNAs in different stages of schistosome development. A PrimeScript RT reagent Kit (Takara) was used to reverse-transcribe the RNA. The stem-loop RT primers were used to reverse-transcribe mature miRNAs (S4 Table). The 10 µL reverse transcription reactions contained approximately 200 ng (6 µL) of total RNA, 2 µL of 5 × PrimeScript Buffer (Takara), 0.5 µL PrimeScript RT Enzyme Mix I (Takara), 1 µL of RNAase inhibitor (Takara), and 0.5 µL of a specific RT primer (10 µM). Temperature was maintained at 42 ℃ for 30 min, 85 ℃ for 5s, and held at 4 ℃. Real-time PCR was performed in a 20 µL reaction mixture containing 2 µL of cDNA (1:5 dilution), 10 µL of 2×SYBR Primer Ex Tag Ⅱ (TaKaRa, China), 6 µL of H_2O, 1 µL of specific forward primers (S4 Table, 10 µM), and 1 µL of a common reverse primer (10 µM). The reactions were amplified using a Master cycler ep realplex (Eppendorf, Germany) real-time PCR detection system, using the thermal cycling profile as follows: 95 ℃ for 10 s, followed by 40 cycles of amplification at 95 ℃ for 5 s, annealing and amplification at 58 ℃ or 60 ℃ for 30s. In parallel, a random primer (Takara) was used to synthesize cDNA for determining the abundance of the mRNA *S. japonicum* nicotinamide adenine dinucleotide dehydrogenase (NADH) as the internal control.

To determine miRNA expression in different regions of female schistosomes, female worms were manually separated and the separated females were dissected into three parts including the anterior, ovary and vitellarium. Total RNAs were isolated from these three regions.. The reverse transcription and PCR reactions were performed using Qiagen's miScript system as described above. All reactions were run in at least triplicate. The $2^{-\Delta Ct}$ method was used to calculate relative miRNA expression.

In situ hybridization

Adult females (28 d) were fixed in 4 % paraformaldehyde (PFA) (Ted Pella) diluted in PBS with 0.3% Triton-X 100 (PBSTx) for 15 min. Worms were reduced in 50 mM DTT, 1% NP-40, and 0.1% SDS for 5-10 min at 37 ℃ and then were dehydrated in a methanol series (100% PBSTx, 50% methanol, and 100% methanol) for 5 min each. The worms were bleached in 6 % hydrogen peroxide diluted from a 30 % stock in methanol under light overnight. Worms were then rinsed twice with 100% methanol, incubated with 50% methanol for 10 min, and subsequently incubated for 10 min in PBSTx. The schistosomes were permeabilized with proteinase K (Invitrogen) at 1 µg/ml in PBS for 15-20 min. After permeabilization, worms were rinsed with PBSTx twice. Prehybridization began with incubation for 10 min in hybridization buffer (50% deionized formamide, 1.3× SSC (diluted from 20× SSC from Sigma, pH=5.5 with citric acid), 5 mM EDTA, 50 µg/µL torula yeast RNA, 0.2%Tween, 0.5% CHAPS, 10% Heparin) mixed with PBSTx at a 1:1 ratio for 10 min. Then, worms were further washed for 10 min with hybridization by incubating at 57 ℃ for 10 min in a hybridization oven (HBAID). Hybridization buffer was then removed and the worms were incubated in fresh hybridization buffer at least 2 h at 57 ℃. Then, the prewarmed LNA (5 µM) (S10 Table) was added into hybridization buffer and incubate overnight at 57 ℃. Then, washes were performed with solutions preheated to 56 ℃ in the following order: Prehybridization solution mixed with 2× SSC

at a 1:1 ratio, 2× SSC with 0.1% Triton-X 100, 0.2× SSC with 0.1% Triton-X 100 in the hybridization oven with agitation. All washes were carried out twice for 30 min each at 58 ℃. After stringency washes, schistosomes were cooled to room temperature and washed twice with maleic acid buffer (100 mM maleic acid, 150 mM NaCl, 0.1% Tween-20, pH 7.5). The worms were then incubated in 10% horse serum for 2 h followed by an overnight incubation at 4 ℃ with an anti-DIG-AP-conjugated antibody (Roche) diluted 1:2000 in blocking solution. Schistosomes were then washed for 2 h in maleic acid buffer (the buffer was changed every 20 min). After washing, worms were incubated in AP buffer (100 mM Tris, pH 9.5; 100 mM NaCl; 50 mM MgCl2; 0.1% Tween-20 in 10% polyvinylalcohol) for 10 min and then were incubated in NBT/BCIP solution for 3 h or overnight. The schistosomes were then washed by PBST and post-fixed in 4 %PFA. Finally, the worms were mounted in 80% glycerol under glass coverslips and imaged.

To determine the localizations of target mRNAs, fragments of Frizzle7 (EU370927.1) and Smad1(AY815078.1) were amplified from cDNA transcribed from total RNA isolated from adult worms (28d) using the primers shown in S10 Table. PCR products cloned into pMD20-T vector (Takara). The recombinant plasmids were used to generate digoxigenin-labeled riboprobes using MEGAscript Kit (Invitrogen) and digoxigenin-11-UTP (Roche). The hybridization was performed at 58℃.

References:

1. Ou M, Zhang X, Dai Y, Gao J, Zhu M, et al. (2014) Identification of potential microRNA-target pairs associated with osteopetrosis by deep sequencing, iTRAQ proteomics and bioinformatics. Eur J Hum Genet 22: 625-632.
2. Langmead B, Trapnell C, Pop M, Salzberg SL (2009) Ultrafast and memory-efficient alignment of short DNA sequences to the human genome. Genome Biol 10: R25.
3. Wang J, Czech B, Crunk A, Wallace A, Mitreva M, et al. (2011) Deep small RNA sequencing from the nematode Ascaris reveals conservation, functional diversification, and novel developmental profiles. Genome Res 21: 1462-1477.
4. Cai P, Hou N, Piao X, Liu S, Liu H, et al. (2011) Profiles of small non-coding RNAs in *Schistosoma japonicum* during development. PLoS Negl Trop Dis 5: e1256.
5. Cai P, Piao X, Hao L, Liu S, Hou N, et al. (2013) A deep analysis of the small non-coding RNA population in *Schistosoma japonicum* eggs. PLoS One 8: e64003.
6. Wang Z, Xue X, Sun J, Luo R, Xu X, et al. (2010) An "in-depth" description of the small non-coding RNA population of *Schistosoma japonicum* schistosomulum. PLoS Neglected Tropical Diseases 4: e596.
7. Eisen MB, Spellman PT, Brown PO, Botstein D (1998) Cluster analysis and display of genome-wide expression patterns. Proc Natl Acad Sci U S A 95: 14863-14868.
8. Cheng G, Luo R, Hu C, Cao J, Jin Y (2013) Deep sequencing-based identification of pathogen-specific microRNAs in the plasma of rabbits infected with *Schistosoma japonicum*. Parasitology 140: 1751-1761.
