# Supplementary material for: Correction: MicroRNAs Are Involved in the Regulation of Ovary Development in the Pathogenic Blood Fluke Schistosoma japonicum
Source: PLoS Pathog. 2016 Apr 19;12(4):e1005582. doi: 10.1371/journal.ppat.1005582 (PMC4836683; doi:10.1371/journal.ppat.1005582)
Supplement: S1 File — (DOCX) [file ppat.1005582.s001.docx]

**Table 2. Validated target genes for *S. japonicum* miRNAs**

| miRNAs | Target gene IDs | Name of target genes | MiRNA:mRNA duplex* | Mfe (kcal/mol) | Validated^#^ |
| --- | --- | --- | --- | --- | --- |
| Bantam | AY223092.1 | Serine-arginine repressor | Target 5` U CUUU A U 3`  GGCU AUC CGAUCUCG  UCGA UAG GCUAGAGU  miRNA 3` AAU C 5` | -22.5 | By miRNA suppression |
|  | FN323394.1 | FUS-interacting serine-arginine-rich protein 1 | Target 5` G UU A U 3`  GGUUUU AUC CGAUCUCG  UCGAAA UAG GCUAGAGU  miRNA 3` U C 5` | -22.6 | By luciferase assay/ miRNA suppression |
|  | AY815078.1 | Smad1 | Target 5` U UG C 3`  GGC UUGAUCGUG CUUA  UCG AAUUAGCGC GAGU  miRNA 3` A UA 5` | -19.9 | By miRNA suppression |
| Let-7 | FN314191.1 | Ribosomal protein S6 kinase 2 | Target 5` A C U A 3`  CAU CAAC GAACUACCUC  GUG GUUG CUUGAUGGAG  miRNA 3`UG U G5` | -28.6 | By luciferase assay |
| miR-2a | FN321618.1 | Plasminogen activator inhibitor 1 | Target 5` A AG CUGAAU C3`  GUUCA GA UUGGCUGUG  CAAGU UU GACCGACAC  miRNA 3`G AG AU U5` | -24.7 | By luciferase assay |
| miR-31 | EU370927 | Frizz7 | Target 5` G G G 3`  UCGUCGUGGUU UUGU  AGCGGCAUUAG AACG  miRNA 3` UCGA GU5` | -22.0 | By luciferase assay |
|  | FN319623.1 | O-glycosyltransferase | Target 5`U C 3`  GCUUU UUGUAAUCUUGCC  CGAAG GGCAUUAGAACGG  miRNA 3` U C U5` | -28 | By miRNA suppression |
| miR-1989 | FN317226 | Asparagine-rich protein | Target 5`A U AUA UA U 3`  C AAGA GUGA ACACAGUUGA  G UUCU UACU UGUGUCGACU  miRNA 3`A C G 5` | -26.4 | By luciferase assay |
| miR-8 | FN313640 | Integral membrane protein GPR177 | Target 5` U AC UUAUUU G3`  GCAUUUU UACCUA AUAGUA  CGUAGAA AUGGAU UGUCAU  miRNA 3`C AAU5` | -22.3 | By luciferase assay |
|  | DQ643829.2 | Wnt | Target 5`C A 3`  GGCAUC ACC GC GUAUUA  CCGUAG UGG UG CAUAAU  miRNA 3` AAA AU U 5` | -20.8 | By luciferase assay |
| miR-3479 | FJ753578.1 | Transforming growth factor receptor II | Target 5` A U ACUUA U3`  CGA GC UAAGUGCAAUA  GUU CG AUUCACGUUAU  miRNA 3` C CUUCC 5` | -20.3 | By luciferase assay |

*miRNA:mRNA pair analysis was performed using RNAhybrid (http://bibiserv.techfak.uni-bielefeld.de/rnahybrid/)

## ^#^luciferase assay = miRNA mimics down-regulate target mRNA sequences in mammalian cells; miRNA suppression = transfection of antisense miRNA sequences into schistosomes leads to increases in target mRNAs
